# FgNup2 regulates nuclear import of the histone H2B monoubiquitination enzyme by stabilizing the FgImpα-FgBre1 complex to mediate pathogenicity in *Fusarium graminearum*

**DOI:** 10.1371/journal.ppat.1014577

**Published:** 2026-09-15

**Authors:** Yaxuan Wang, Zhe Li, Xiaoyan Wang, Bin Xiong, Chengqi Zhang, Xiaozhen Zhao, Shentao Wang, Li Chen

**Affiliations:** 1 School of Plant Protection, Anhui Agricultural University, Hefei, China; 2 Key Laboratory of Agri‐Products Quality and Biosafety (Anhui Agricultural University), Ministry of Education, Hefei, China; University of Cambridge, UNITED STATES OF AMERICA

## Abstract

Fusarium head blight caused by *Fusarium graminearum* threatens global wheat production. The pathogenicity of this fungus depends on histone H2B monoubiquitination (H2Bub1), yet the nuclear import mechanism of its key enzyme, FgBre1, remains unclear. In this study, we identified 24 nucleoporins in *F. graminearum* and found that *FgNup2* is essential for fungal growth, toxin biosynthesis, and virulence. FgNup2 positively regulates H2Bub1 levels by facilitating the nuclear import of FgBre1, thereby influencing pathogenic development. The nuclear import of FgBre1 relies on the importin receptor FgImpα, and these two proteins interact. FgNup2, through a critical phenylalanine residue (F981) within its FG-repeat domain, promotes and stabilizes the FgImpα–FgBre1 complex, driving the assembly of the tripartite FgNup2–FgImpα–FgBre1 complex. This ultimately mediates the rapid nuclear translocation of FgBre1 under induction by the trichothecene biosynthesis-inducing medium. Our findings reveal a mechanism by which a nucleoporin regulates the nuclear entry of a histone-modifying enzyme via stabilizing the importin–cargo complex, advancing the understanding of non-canonical roles of the nuclear pore complex in pathogen adaptation.

## Author summary

Fusarium head blight, caused by the fungal pathogen *F. graminearum*, is a devastating disease that leads to significant wheat yield losses and contamination of grains with mycotoxins. The pathogenicity of this fungus is associated with the H2Bub1 modification, which is executed by the enzyme FgBre1. For FgBre1 to function, it must be imported into the nucleus. However, the regulatory mechanisms governing its nuclear import have remained unclear. Here, we identified FgNup2, a component of the nuclear pore complex, as a master regulator of fungal growth, toxin production, and the ability to infect wheat. Beyond serving as a passive gatekeeper, FgNup2 actively facilitates the nuclear entry of FgBre1. It acts as a molecular scaffold, stabilizing the interaction between FgBre1 and the nuclear transport shuttle protein FgImpα. Within FgNup2, the amino acid residue F981 was found to be critical for maintaining this stability. Our study reveals a mechanism by which a pathogen regulates key biological processes through modulating nuclear pore transport function, offering a novel target for developing intervention strategies aimed at curbing the virulence of this fungus.

## 1 Introduction

The pathogenicity of filamentous fungi relies on their ability to rapidly sense and adapt to the host microenvironment, a process mediated by complex signal transduction networks and transcriptional regulatory mechanisms [1,2]. The nuclear pore complex (NPC), a massive protein complex embedded in the nuclear envelope [3], serves as the exclusive bidirectional transport channel for macromolecules (including mRNAs, transcription factors, and ribosomal subunits) between the nucleus and cytoplasm [3–6]. Consequently, the NPC occupies a central position in regulating fungal adaptive gene expression. As the key gateway for nucleocytoplasmic transport, the NPC controls the expression of pathogenesis-related genes by modulating the shuttling of key transcription factors. For example, in the rice blast fungus *Magnaporthe oryzae*, MoSec13 localizes to multiple regions including the perinuclear area. It interacts with autophagy-related proteins MoAtg7/MoAtg8 and regulates the acetylation state of MoAtg8, thereby integrating the autophagy network to affect fungal growth and pathogenicity [7]. Furthermore, the *F. graminearum* nucleoporin FgNup2 directly interacts with the core transcription factor for toxin synthesis, FgTri6, to co-regulate the expression of trichothecene biosynthesis genes. Deletion of *FgNup2* significantly reduces fungal pathogenicity and deoxynivalenol (DON) production, indicating that loss of specific nucleoporins can disrupt the nucleocytoplasmic distribution and function of key virulence regulators [8]. These studies suggest that nucleoporins may act as critical nodes determining the pathogenicity and host adaptation of filamentous fungi by regulating macromolecular transport or their own assembly state.

The nucleus, as the center for genetic information storage and transcription, requires efficient material exchange between the NPC and nuclear transport receptors (NTRs) [3]. In filamentous fungi, NTRs are mainly divided into importins and exportins. Nuclear import is primarily mediated by the Importinα/β heterodimer: Importinα recognizes the classical nuclear localization signal (NLS) of cargo proteins, while Importinβ interacts with phenylalanine-glycine (FG) repeat-rich nucleoporins (FG-Nups) to drive the complex through the NPC [9,10]. Nuclear export is mediated by export receptors such as Crm1/Xpo1, which recognize cargo proteins containing a leucine-rich nuclear export signal (NES) [11–13]. The transport core lies in the transient, reversible interactions between NTRs and FG-Nups, allowing receptor-cargo complexes to rapidly traverse the NPC [13–15]. Notably, the composition, density, and distribution of FG repeats vary among different FG-Nups, determining their affinity for specific transport receptors and enabling selective cargo regulation [16,17]. For instance, replacing filamentous fungal FG repeats with yeast or mammalian homologs, or deleting part of the FG domain, can specifically interfere with receptor recognition, leading to phenotypes such as aberrant infection ability and increased sensitivity to plant defense compounds.

The cascade regulation of histone modifications is a core feature of epigenetic networks. The pathway centered on H2Bub1 is conserved across eukaryotes [18–20]. In this cascade, H2Bub1 is a prerequisite for subsequent H3K4 and H3K79 methylation, forming an ordered signaling chain for transcriptional activation [21]. H2Bub1 is directly regulated by the specific ubiquitin-conjugating enzyme (E2) Rad6 and the ubiquitin ligase (E3) Bre1 [22]. In filamentous fungi, knockout of the Bre1 homolog leads to H2Bub1 deficiency and impairs hyphal growth, proving its critical role. H2Bub1 is essential for the COMPASS complex to catalyze H3K4me3 and for the Dot1 enzyme to catalyze H3K79me2/3 [23–25]. During infection, *F. graminearum* rapidly responds to the plant stress-response substance putrescine. This response promotes the binding of the H2Bub1 regulatory component FgBre1 to chromatin regions of DON toxin synthesis-related genes (*FgTRIs*), thereby promoting H2Bub1 deposition. Furthermore, FgRad6 and FgBre1 recruit the H3K4 methyltransferase complex to the corresponding chromatin regions, activating the methyltransferase FgSet1 to promote H3K4me2/me3 deposition. This process activates *FgTRIs* gene transcription, enabling the fungus to synthesize large amounts of DON toxin to facilitate disease expansion [26].

*F. graminearum* is a globally significant pathogen of wheat and maize. The Fusarium head blight it causes leads to substantial yield losses [27,28], and poses a serious threat to food safety due to its production of type B trichothecene toxins, primarily DON [29,30]. The pathogenicity of *F. graminearum* is regulated at multiple, interconnected levels, involving environmental signal perception, nucleocytoplasmic transport, epigenetic modifications, and the precise expression of the trichothecene biosynthesis *TRIs* cluster. Although key components in these processes have been identified, the specific molecular bridges connecting different regulatory layers and their synergistic mechanisms remain unclear. In particular, whether key molecules exist to integrate environmental signals, NPC function, epigenetic modifications, and *TRI* gene expression lacks systematic investigation.

This study elucidates the molecular mechanism by which the nucleoporin FgNup2 regulates the nuclear import of the histone H2Bub1 enzyme FgBre1 in the plant pathogenic fungus *F. graminearum*. Our primary objective was to determine how FgNup2 acts upstream of FgBre1 to control H2Bub1 levels and, consequently, fungal pathogenicity. To dissect this upstream regulatory mechanism, we investigated the role of the classical nuclear import receptor FgImpα in mediating FgBre1 nuclear transport. We further evaluated the functional significance of the interactions among FgNup2, FgImpα, and FgBre1. A key focus was to clarify the role of specific residues within FgNup2 in stabilizing the FgImpα–FgBre1 complex and promoting the assembly of the FgNup2–FgImpα–FgBre1 ternary complex. The significance of this research lies in revealing a non-canonical, active role for a nucleoporin. We demonstrate that FgNup2 can directly stabilize the nuclear import receptor–cargo complex to ensure the efficient transport of a key histone modifier. This finding expands our understanding of NPC function beyond passive nucleocytoplasmic transport, uncovering a new regulatory layer in fungal adaptive responses. Ultimately, this work provides potential novel targets for strategies aimed at controlling this devastating crop disease.

## 2 Results

### 2.1 Systematic identification of the nucleoporin family in *F. graminearum* and expression analysis during wheat infection

A systematic identification of nucleoporins (FgNups) in the *F. graminearum* genome was performed. Using *Saccharomyces cerevisiae* and *Neurospora crassa* nucleoporins as a reference, NCBI BlastP analysis identified 24 non-redundant candidate FgNups (Table S1 and S2 in S1 Appendix). Conserved domain analysis revealed that these FgNups proteins lack a consistent common domain but share certain specific domains among some members (S1 Fig). To further elucidate their evolutionary relationships, a phylogenetic tree containing ScNups and FgNups was constructed. The results showed that this protein family can be divided into six distinct clades (Fig 1A). Total of 20 nucleoporins, cluster on the same evolutionary branch as their homologues in *S. cerevisiae*, indicating that their functions may be relatively conserved in fungi. FgNup188, FgNup2, FgNup159 and FgNup82 are distributed on different branches from their homologues in *S. cerevisiae*, suggesting that they may have undergone functional differentiation or possess species-specific traits during evolution. To explore the potential functions of *FgNups* during infection, their expression patterns were analyzed from 0 to 7 d post-inoculation of wheat. Transcriptome data revealed that 10 *FgNups* genes were specifically upregulated during the infection stages (Fig 1B and S2 Fig). Subsequently, knockout mutants for these 10 genes (S3 Fig) were constructed and preliminarily assessed for vegetative growth phenotype (Fig 1C, D) and pathogenicity on wheat spikes (Fig 1E-G). Phenotypic analysis showed that among the 10 mutants, only the *ΔFgNup2* mutant exhibited significant defects in hyphal growth and a marked reduction in pathogenicity.

**Fig 1:**
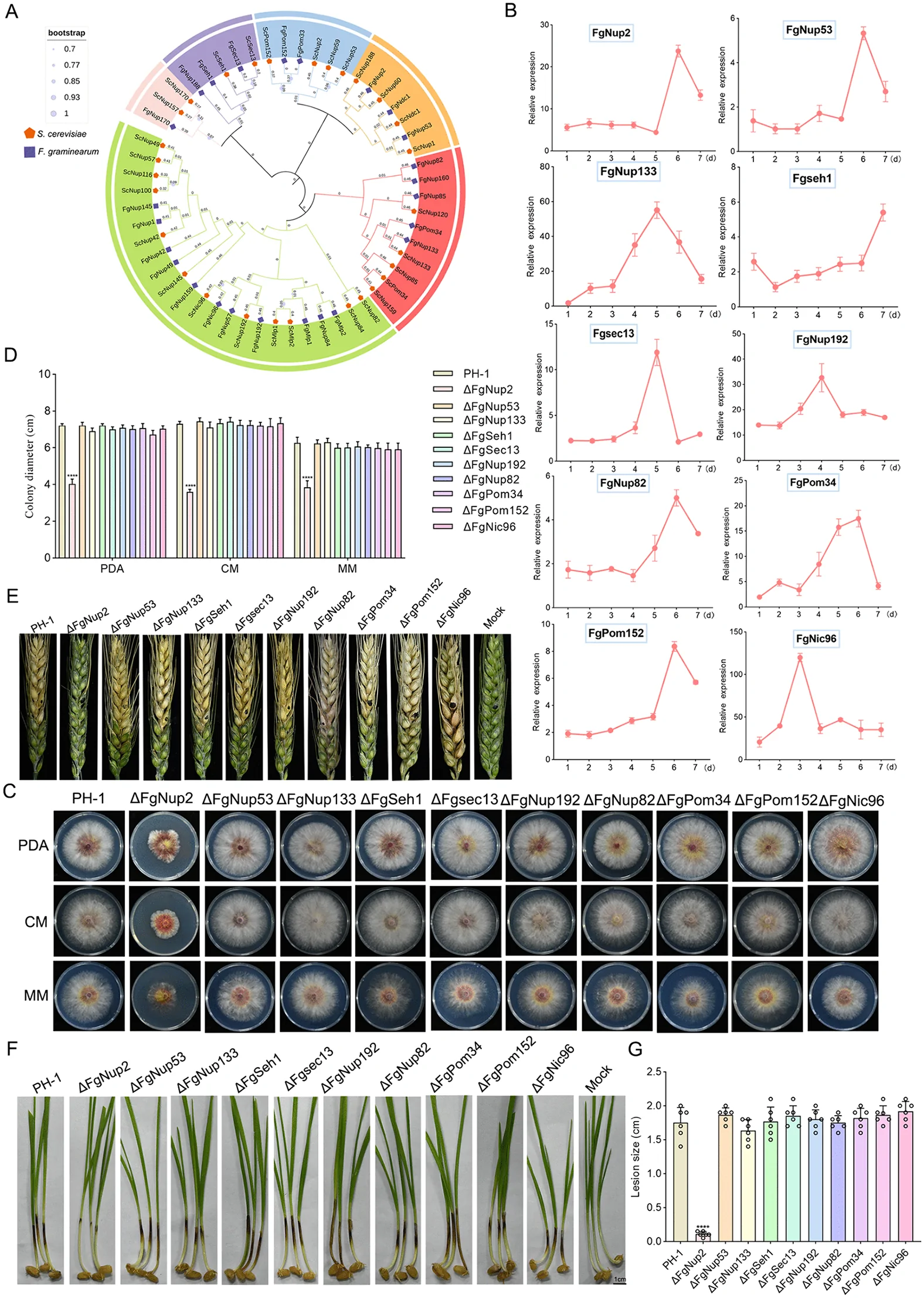
Systematic identification of the nucleoporin family in *F.graminearum* and analysis of their expression during wheat infection. **(A)** Phylogenetic analysis of *F. graminearum* and *S. cerevisiae* Nups. The phylogenetic tree was obtained using MEGA-X. *F. graminearum*: Fg, purple square, *S. cerevisiae*: Sc, orange pentagon. **(B)** Analysis of FgNups expression levels during wheat infection from 0-7 **d. (C)** Morphology of PH-1, *ΔFgNup2*, *ΔFgNup53*, *ΔFgNup133*, *ΔFgSeh1*, *ΔFgSec13*, *ΔFgNup192*, *ΔFgNup82*, *ΔFgPom34*, *ΔFgPom152*, and *ΔFgNic96* after 72 h of growth on PDA, CM, and MM media. **(D)** Colony diameters of the above strain on three different media after 3 d of growth, the data come from three independent repeated experiments, and significant differences are indicated by * (****P ≤ 0.0001). **(E)** Pathogenicity of the above strain on field wheat spikes. **(F)** Pathogenicity of the above strain on wheat coleoptiles. **(G)** Statistical analysis of lesion length on wheat coleoptiles infected by the above mutant strains, the data come from three independent repeated experiments, and significant differences are indicated by * (****P ≤ 0.0001).

### 2.2 *FgNup2* regulates development, stress response, pathogenicity, and deoxynivalenol (DON) biosynthesis in *F. graminearum*

The deletion of *FgNup2* resulted in significant hyphal growth defects, which were fully restored in the complemented strain (Fig 2A, B). Compared to the wild-type PH-1 and the complementation strain, the *ΔFgNup2* mutant produced conidia with significantly altered morphology, characterized by increased length and decreased diameter (Fig 2C). Furthermore, the number of conidia produced by the mutant was significantly reduced (Fig 2D). In the wild-type strain PH-1, each compartment of the conidia usually contains only one nucleus (occasionally two). However, in the *ΔFgNup2* mutant, we observed the abnormal presence of multiple nuclei in each compartment. Furthermore, nuclei in PH-1 hyphae are evenly distributed, while in the *ΔFgNup2* mutant, nuclei often cluster together, suggesting abnormal nuclear division in this mutant. These results indicate that FgNup2 is crucial for maintaining normal nuclear distribution and division(Fig 2E). The *ΔFgNup2* mutant exhibited enhanced tolerance to cell wall and cell membrane stressors (Congo Red and SDS) but increased sensitivity to osmotic and metal ion stress (Fig 2F, G). Pathogenicity assays were conducted on wheat spikes and coleoptiles using equal concentrations of conidial suspensions. Inoculation with the *ΔFgNup2* mutant failed to produce any disease symptoms compared to PH-1 and *ΔFgNup2-C* (Fig 2H, I). DON production was quantified. The amount of DON produced by the *ΔFgNup2* mutant was significantly lower than that of the wild-type strain (Fig 2J).

**Fig 2:**
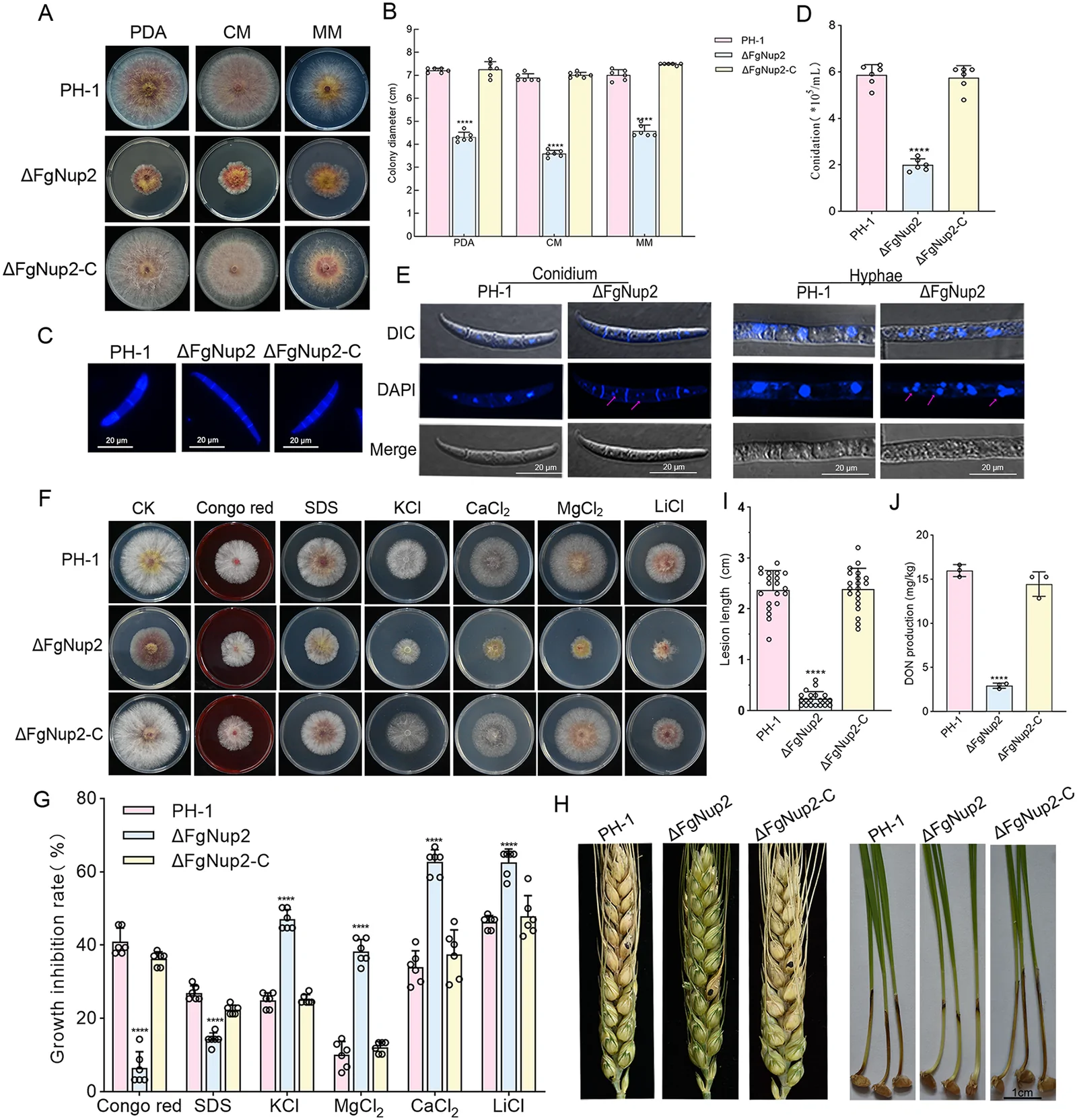
The nucleoporin *FgNup2* regulates development, stress response, pathogenicity, and DON biosynthesis in *F. graminearum.* **(A)** Colony morphology of PH-1, *ΔFgNup2* and *ΔFgNup2-C* grown on PDA, CM and MM media for 72 **h. (B)** Colony diameter statistics of PH-1, *ΔFgNup2* and *ΔFgNup2-C* grown on PDA, CM and MM media for 72 **h.** The data come from three independent repeated experiments, and significant differences are indicated by * (****P ≤ 0.0001) **(C)** *ΔFgNup2* affects conidia number. CFW staining, DIC morphology observed under a fluorescence microscope, Bar = 20 μm. **(D)** Conidia production of PH-1, *ΔFgNup2*, and *ΔFgNup2-C* after 4 d of cultivation on CMC medium. **(E)** The nucleoporin *FgNup2* is involved in regulating mitosis in *F. graminearum*. **(F)** The morphology of PH-1, *ΔFgNup2*, and *ΔFgNup2-C* colonies grown on PDA medium containing different stress agents. **(G)** Hyphal growth inhibition rates of PH-1, *ΔFgNup2*, and *ΔFgNup2-C* strains grown under stress conditions after 3 d, the data come from three independent repeated experiments, and significant differences are indicated by * (****P ≤ 0.0001). **(H)** Pathogenicity of PH-1, *ΔFgNup2*, and *ΔFgNup2-C* on wheat ears and wheat coleoptiles. **(I)** Statistics of wheat coleoptiles lesion length, the data come from three independent repeated experiments, and significant differences are indicated by * (****P ≤ 0.0001). **(J)** The amount of DON (per g dry weight) produced by the wild-type PH-1, *ΔFgNup2* and *ΔFgNup2-C* in trichothecene biosynthesis-inducing (TBI) medium after 48 h inoculation, the data come from three independent repeated experiments, and significant differences are indicated by * (****P ≤ 0.0001).

### 2.3 FgNup2 interacts with the ubiquitin ligase FgBre1

RNA sequencing was performed on the wild-type strain PH-1 and the *ΔFgNup2* mutant cultured for 48 h in trichothecene biosynthesis-inducing (TBI) medium, which contains arginine as the sole nitrogen source. This medium was previously used by Gardiner et al. to investigate the effect of humic acid on DON production [31]. Using a threshold of p-value<0.05 and |log2(fold change)| ≥ 2, we identified a total of 3,807 differentially expressed genes (DEGs) in the *ΔFgNup2* mutant compared to PH-1. Among these, 2,075 genes were significantly upregulated and 1,732 were downregulated (Fig 3A). A heatmap of RPKM-normalized transcripts depicting the 3,807 DEGs is shown in S4 Fig. Functional annotation of these DEGs indicated their involvement in various biological processes, including carbohydrate, lipid, nucleotide, and amino acid metabolism. Cell cycle regulation; DNA replication and transcription, post-translational modification. Intracellular transport and the biosynthesis of secondary metabolites, cell wall and cell membrane (Fig 3B and C). An affinity capture approach was employed to identify its interaction partners. The N-terminal region of FgNup2 was fused to GFP and introduced into the *ΔFgNup2* mutant. Proteins purified using GFP-trap agarose beads were analyzed by mass spectrometry (MS). By comparing the MS data with the *F. graminearum* PH-1 database, candidate FgNup2-interacting proteins were identified. Using a confidence threshold of false discovery rate (FDR)≤1% and more than 5 peptide-spectrum matches (PSMs), a list of 300 candidate interacting proteins was obtained. RNA-seq analysis revealed widespread dysregulation of transcription-related genes in *ΔFgNup2*. Among the 300 FgNup2-interacting candidates identified by affinity capture–mass spectrometry, we prioritized proteins with chromatin-regulatory functions, including FGSG_06769, which encodes the histone H2B ubiquitin ligase FgBre1 responsible for H2B monoubiquitination (H2Bub1) in *F. graminearum*, as the key candidate for further validation. The physical interaction between FgNup2 and FgBre1 was subsequently confirmed by multiple independent experiments, including yeast two-hybrid (Y2H) analysis, co-immunoprecipitation (Co-IP), GST-pull-down, and bimolecular fluorescence complementation (BiFC) assays (Fig 3D-G).

**Fig 3:**
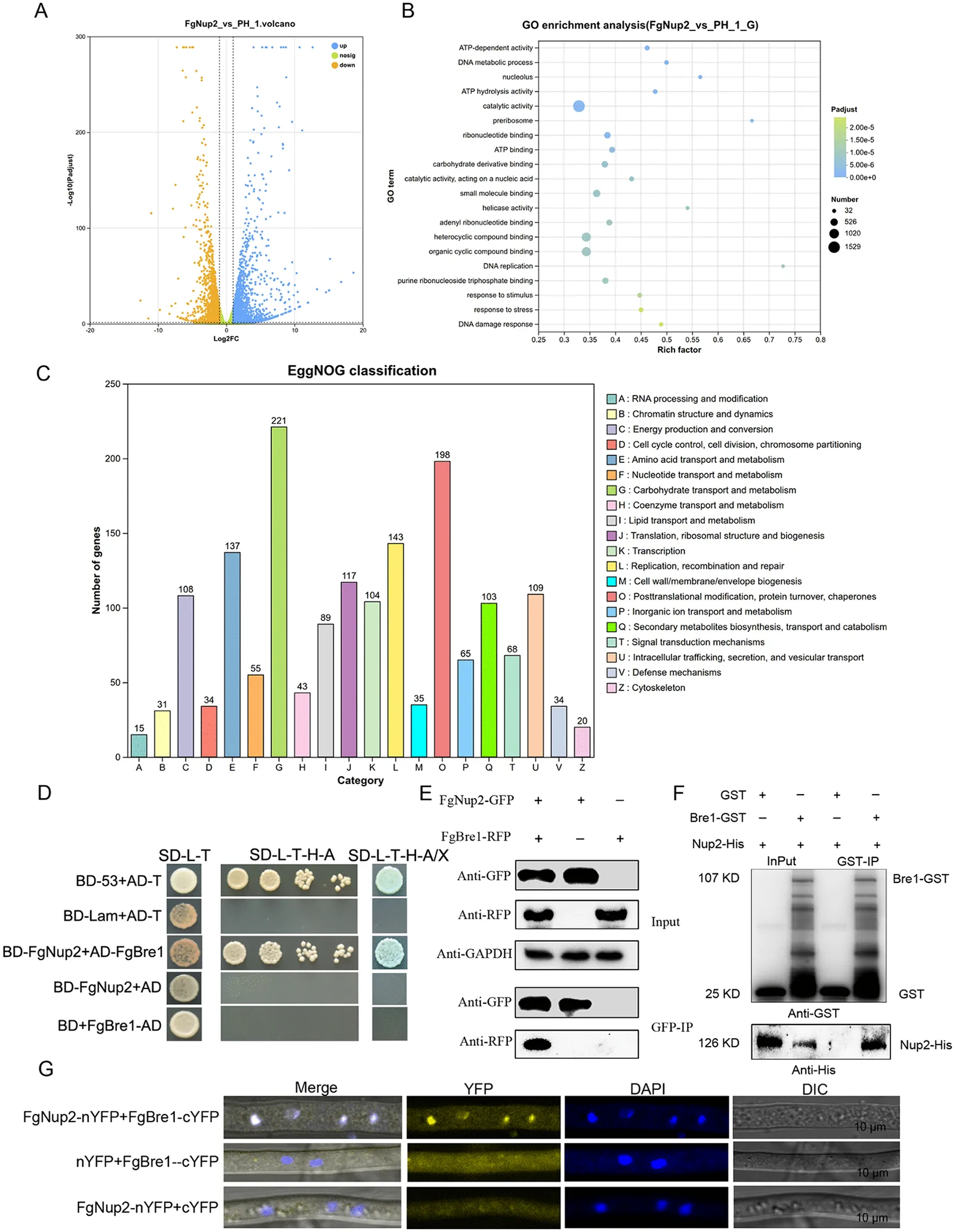
FgNup2 interacts with the ubiquitin ligase FgBre1. **(A)** Volcano plot showing the number of genes with more than twofold expression change in *ΔFgNup2*. **(B)** GO enrichment analysis of differential genes. **(C)** EggNOG functional classification of differentially expressed genes in *ΔFgNup2*. **(D)** Yeast two-hybrid (Y2H) experiment verifying the interaction between FgNup2 and FgBre1. **(E)** Co-immunoprecipitation (Co-IP) experiment verifying the interaction between. **(F)** GST pull-down experiment verifying the interaction between FgNup2 and FgBre1. **(G)** Bimolecular fluorescence complementation (BiFC) assays verifying the interaction between FgNup2 and FgBre1.

### 2.4 FgNup2 regulates FgBre1-mediated histone modification

Based on the direct interaction between FgNup2 and FgBre1, we hypothesized that FgNup2 may participate in regulating H2Bub1 modification levels. To test this hypothesis, we examined H2Bub1 abundance in the wild-type strain PH-1 and the *ΔFgNup2* mutant by Western blot. Under TBI conditions, H2Bub1 levels were markedly elevated in the wild type but significantly reduced in the *ΔFgNup2* mutant compared to PH-1(Fig 4A, B). Notably, FgNup2 deletion did not affect FgBre1 protein expression, as confirmed by Western blot and qRT-PCR analyses (S5 Fig), excluding the possibility that the observed decrease in H2Bub1 resulted from reduced FgBre1 abundance.

**Fig 4:**
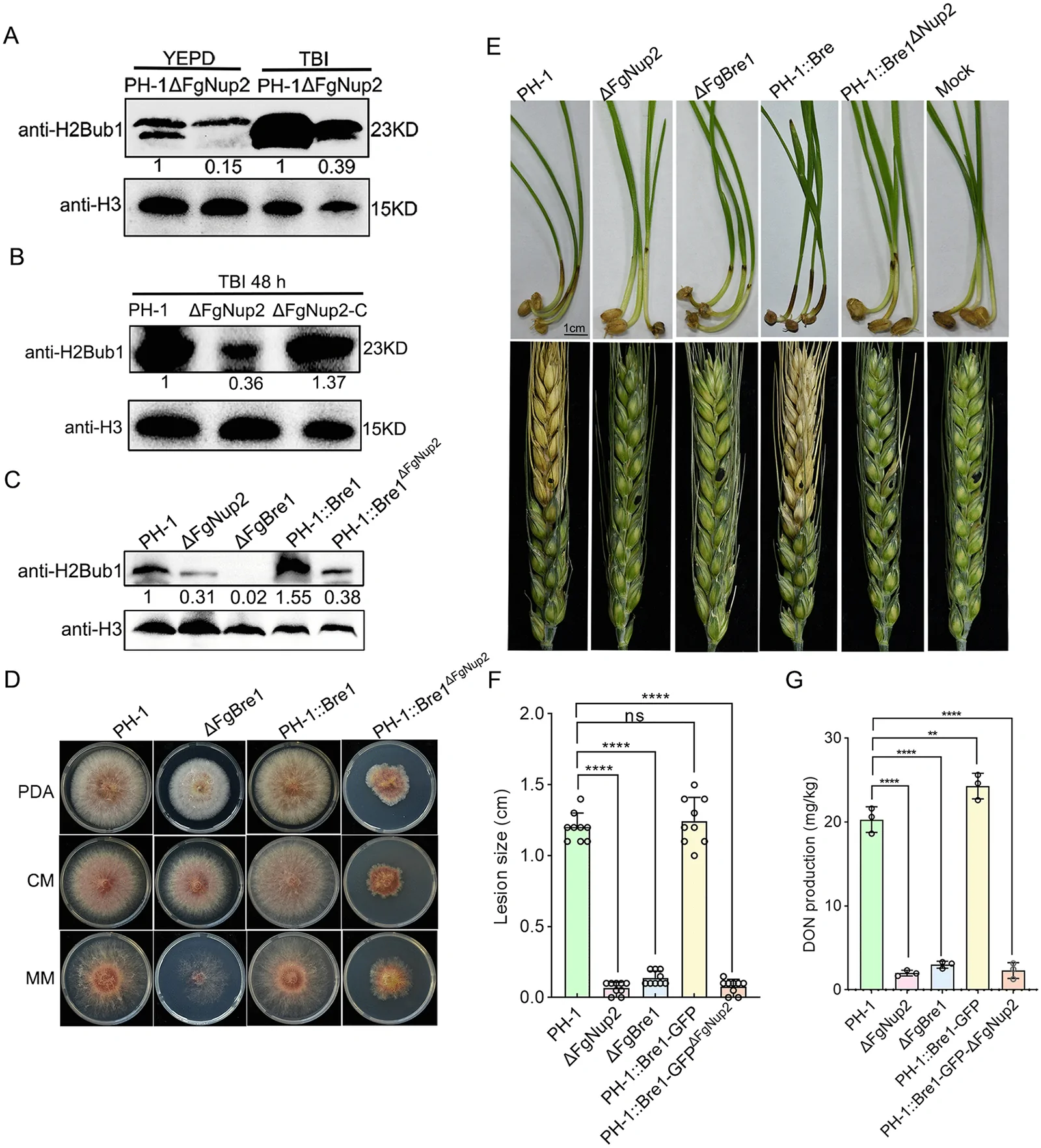
FgNup2 regulates FgBre1-mediated histone modification” would be more appropriate. **(A)** Western blot experiment to verify the levels of H2Bub1 modification in PH-1 and *ΔFgNup2* strains cultured under YEPD or TBI medium conditions. **(B)** Western blot experiment to verify the levels of H2Bub1 modification in PH-1, *ΔFgNup2* and *ΔFgNup2-C* strains 48 h after under toxin-inducing conditions. **(C)** FgNup2 promotes the occurrence of H2Bub1 in *F. graminearum* through FgBre1. H3 was used as the internal control, and the intensity of the Western blot bands was quantified using ImageJ software. The values below the bands indicate the grey value of the detected protein bands relative to the H3 bands. **(D)** Colony morphology of PH-1, *ΔFgBre1*, PH-1::Bre1, and PH-1::Bre1^ΔFgNup2^ after 72 h of growth on PDA, CM, and MM media. **(E)** Pathogenicity of PH-1, *ΔFgNup2*, *ΔFgBre1*, PH-1::Bre1 and PH-1::Bre1^ΔFgNup2^ on wheat heads and wheat coleoptiles. **(F)** Statistical analysis of lesion lengths on wheat coleoptiles infected by PH-1, *ΔFgNup2*, *ΔFgBre1*, PH-1::Bre1 and PH-1::Bre1^ΔFgNup2^, the data come from three independent repeated experiments, and significant differences are indicated by * (****P ≤ 0.0001). **(G)** Statistical analysis of DON toxin production induced in TBI medium for 48 h by PH-1, *ΔFgNup2*, *ΔFgBre1*, PH-1::Bre1 and PH-1::Bre1^ΔFgNup2^, the data come from three independent repeated experiments, and significant differences are indicated by * (****P ≤ 0.0001, **P ≤ 0.01, ns *P ≥ 0.05*).

To determine whether FgNup2 regulates H2Bub1 through FgBre1, we constructed and compared four strains: the FgBre1 deletion mutant *ΔFgBre1*, the FgBre1 overexpression strain PH-1::FgBre1, and FgBre1 overexpression in the *ΔFgNup2* background(PH-1::FgBre1^ΔFgNup2^). Western blot analysis of H2Bub1 levels in these strains (Fig 4C) revealed the following: *ΔFgBre1* showed virtually no detectable H2Bub1 signal, consistent with previous reports and confirming that FgBre1 is the essential for this modification. PH-1::FgBre1 exhibited significantly elevated H2Bub1 levels relative to the wild type, demonstrating that FgBre1 overexpression alone is sufficient to enhance H2Bub1, however, in PH-1::FgBre1^ΔFgNup2^, despite FgBre1 overexpression, H2Bub1 levels were drastically reduced and comparable to those in the *ΔFgNup2* single mutant. Furthermore, the PH-1::FgBre1^ΔFgNup2^ strain displayed significantly reduced growth rate, pathogenicity, and DON toxin production compared to both the wild type and the FgBre1 overexpression strain (Fig 4D-G). Collectively, these findings demonstrate that FgNup2 is required for FgBre1-mediated H2Bub1 modification.

### 2.5 FgNup2 spatiotemporally regulates the nuclear import of FgBre1

Sequence analysis revealed that the N-terminus of FgBre1 contains a conserved putative nuclear localization signal (S6A Fig), suggesting that its nuclear import depends on nuclear pore trafficking. To investigate whether FgNup2 regulates the nuclear transport of FgBre1, we observed the subcellular localization of FgBre1-GFP in the wild-type PH-1 and the *ΔFgNup2* mutant under TBI medium induction. In the wild-type strain, FgBre1 rapidly accumulated in the nucleus, with nearly complete nuclear import observed as early as 1 h toxin biosynthesis induction (Fig 5A). In stark contrast, the nuclear import of FgBre1 was significantly delayed in the *ΔFgNup2* mutant. At 6 h, FgBre1 was primarily retained in the perinuclear region and failed to efficiently enter the nucleoplasm. Partial nuclear import was detected only at 12 h, and significant nuclear accumulation appeared at 24 h (Fig 5A). This delayed import phenotype was consistently observed in protoplasts prepared from the PH-1::Bre1-GFP and PH-1::Bre1-GFP^ΔFgNup2^ strains, indicating it was not an artifact of hyphal morphology (Fig 5B). Subcellular fractionation followed by western blot analysis was performed to assess FgBre1 levels in nuclear and cytoplasmic extracts. The results were consistent with the fluorescence microscopy observations. At early induction stages, the mutant showed significantly reduced nuclear accumulation of FgBre1 compared to the wild-type, while its cytoplasmic levels remained relatively high (Fig 5C), We collected the hyphae of *F. graminearum* infecting the wheat leaves during the infection process, and observed the subcellular localization of FgBre1 through DAPI staining. The results showed that under natural infection conditions, FgBre1 was mainly located in the nucleus. While in the *ΔFgNup2* mutant background, FgBre1 significantly accumulated in the perinuclear region (S6B, S6C Fig). Based on these findings, a schematic diagram illustrating the regulation of FgBre1 nuclear import by FgNup2 was drawn (Fig 5D).

**Fig 5:**
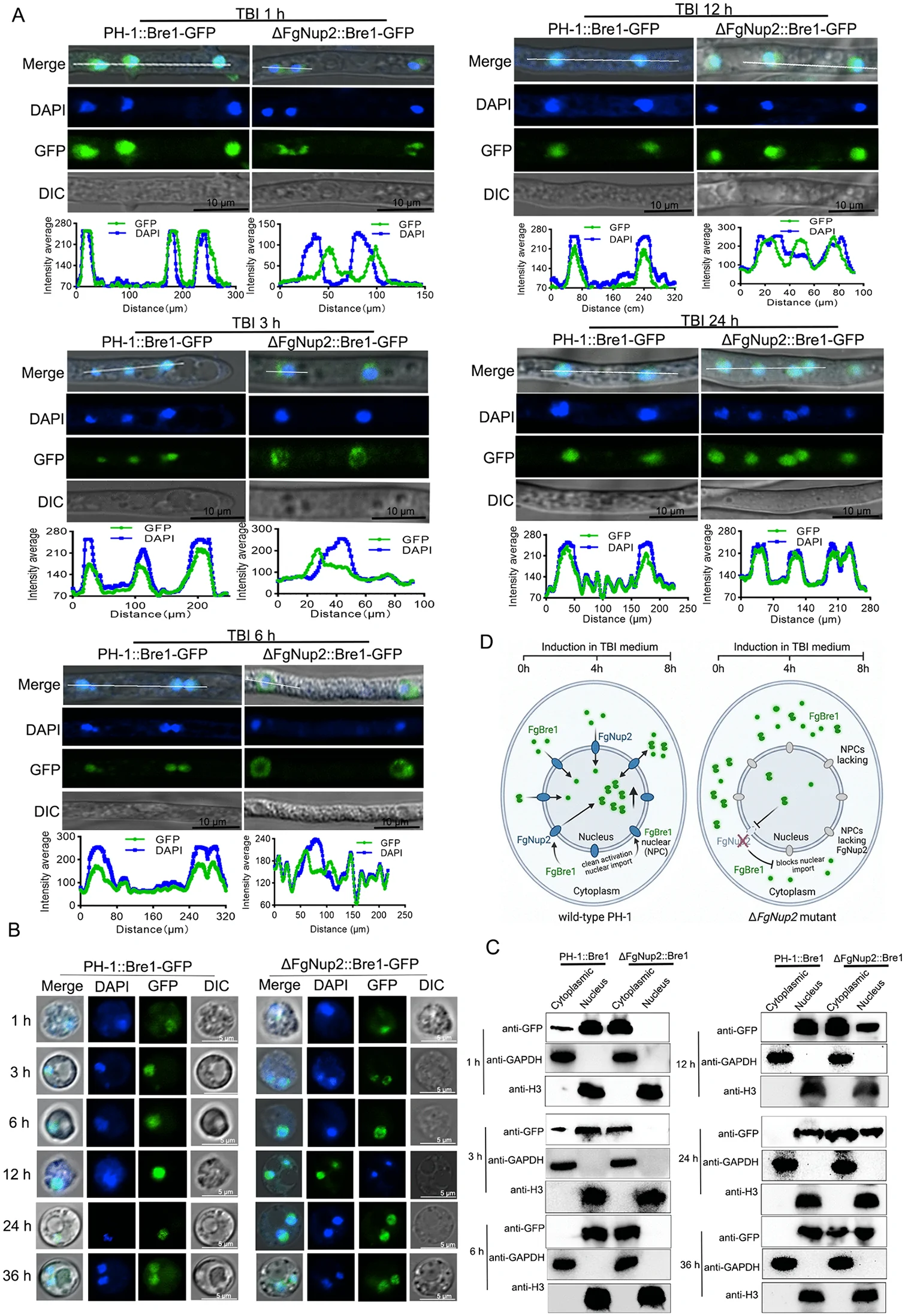
Spatiotemporal regulation of FgBre1 nuclear entry by FgNup2 under toxin-inducing conditions. **(A)** The localisation of FgBre1-GFP in the hyphae of PH-1 and *ΔFgNup2* strains induced in TBI medium for 1 h, 3 h, 6 h, 12 h, 24 h, Bar = 10 μm, fluorescence intensity analysed with ImageJ software. **(B)** The localisation of FgBre1-GFP in the protoplasts of PH-1 and *ΔFgNup2* strains induced in TBI medium for 1 h, 3 h, 6 h, 12 h, 24 h and 36 h, Bar = 10 μm. **(C)** Western blot experiment quantifying the content of FgBre1-GFP protein in nuclei and cytoplasm at 1 h, 3 h, 6 h, 12 h, 24 h and 36 h of induction in TBI medium. **(D)** Schematic diagram of the pattern of FgBre1 nuclear import regulated by FgNup2.

### 2.6 FgNup2 cooperates with FgImpα to regulate the nuclear localization of FgBre1

Fglmpa is a member of an importin protein family widely found in filamentous fungi, usually able to recognise nuclear localisation signals (NLS) and mediate the nuclear import of cargo proteins. Given that FgBre1 contains a predicted NLS, we speculate that Fglmpa might act as its nuclear import adaptor. we first performed a Co-IP assay. The results showed an interaction between FgBre1 and FgImpα under TBI medium conditions (Fig 6A, B). As a *ΔFgImpα* knockout mutant was unavailable, a competitive inhibition strategy was developed by overexpressing a 3 × Flag-tagged peptide containing a nuclear localization signal (NLS) and a nuclear export signal (NES). This NLS-NES construct acts as a high-affinity competitive cargo for FgImpα. Western blot analysis confirmed the successful expression of the Flag-tagged inhibitor (Fig 6C). In the PH-1/Impα-GFP strain, FgImpα-GFP was primarily localized to the nucleus after 36 h of toxin biosynthesis induction. However, in the presence of the NLS-NES competitor (PH-1/Impα-GFP + NLS), FgImpα-GFP became diffusely distributed throughout the cytoplasm (Fig 6D), indicating effective competition for Impα binding. Next, we examined the effect of this competition on FgBre1 localization. In the PH-1/Bre1-RFP Impα-GFP strain, FgBre1-RFP and FgImpα-GFP showed complete nuclear co-localization. In contrast, in the strain co-expressing the NLS-NES competitor (PH-1/Bre1-RFP+Impα-GFP + NLS), the co-localization of FgBre1 with FgImpα was significantly reduced (Fig 6B). Consistent with this, CO-IP assays showed that the interaction between FgBre1 and FgImpα was significantly weakened upon introduction of the NLS-NES competitor (Fig 6E). Together, these data indicate that the nuclear import of FgBre1 relies on functional FgImpα-mediated transport. Furthermore, fluorescence co-localization analysis revealed that the degree of co-localization between FgBre1 and FgImpα was significantly reduced in the *ΔFgNup2* background (Fig 6F). Co-IP assays showed that the binding strength between FgBre1 and FgImpα was significantly lower in the *ΔFgNup2* mutant compared to the wild-type strain (Fig 6G).

**Fig 6:**
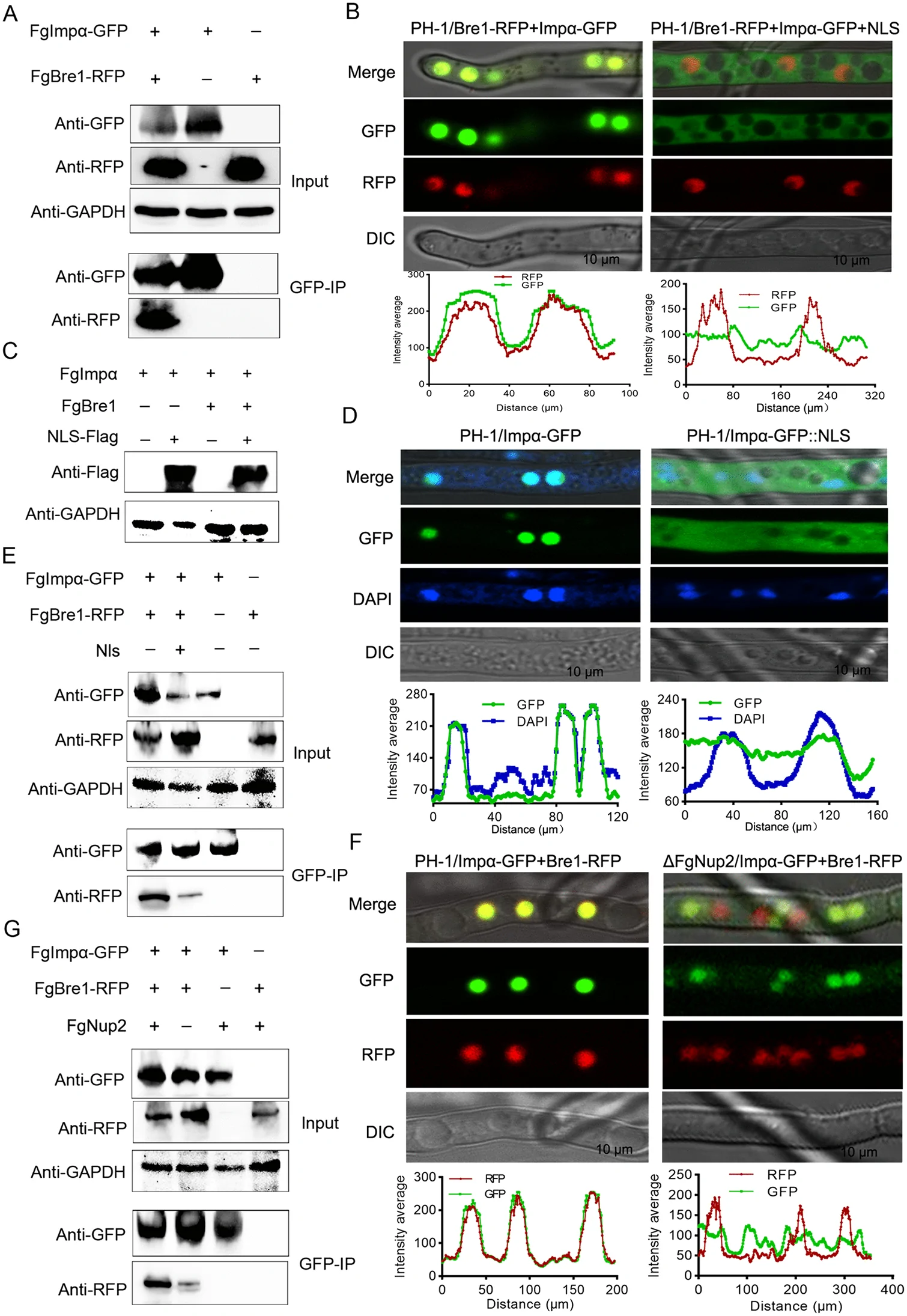
FgNup2 and FgImpα synergistically regulate the nuclear localisation of FgBre1. **(A)** Co-immunoprecipitation (Co-IP) experiment confirmed the interaction between FgBre1 and FgImpα. **(B)** Effect of competitive inhibitors on the co-localization of FgBre1 with FgImpα. In the PH-1/Bre1-RFP Impα-GFP strain, significant fluorescence co-localization signals of FgBre1 and FgImpα were observed in the nuclear region, whereas in the PH-1/Bre1-RFP Impα-GFP NLS strain, the co-localization of FgBre1 and FgImpα was significantly weakened. Bar = 10 μm. Co-localization and fluorescence intensity were analyzed using ImageJ software. **(C)** Using Western Blot with Flag antibody to detect the expression of the 3 × Flag-NLS-NES competitive inhibitor vector in the PH-1 background strain. **(D)** Confocal laser microscopy observation of the effect of the inhibitor on the subcellular localisation of FgImpα-GFP. **(E)** Co-immunoprecipitation (Co-IP) detection of the effect of the inhibitor on the interaction between FgBre1 and FgImpα. **(F)** Under under toxin-inducing conditions, laser confocal microscopy was used to observe the fluorescence localisation and colocalisation of FgBre1-RFP and FgImpα-GFP in PH-1 and *ΔFgNup2* strains, Bar = 10 μm. **(G)** Co-immunoprecipitation (Co-IP) experiments were used to detect the interaction between FgBre1 and FgImpα in PH-1 and *ΔFgNup2* strains.

### 2.7 *FgNup2* regulates development, stress response, and virulence in *F. graminearum* by promoting H2Bub1

To further investigate the impact of *FgNup2* on H2Bub modification, ChIP-seq experiments were performed on PH-1 and *ΔFgNup2* strains under TBI medium conditions. To ensure the reliability of H2Bub1 profiling, we performed comprehensive quality control for all ChIP-seq samples (S7 Fig). The sequencing depth was adequate across all ChIP samples (15.4–27.5 million reads). PCA analysis demonstrated tight clustering of biological replicates within each genotype and clear separation between PH-1 and *ΔFgNup2*, confirming high data reproducibility. RiP% values ranged from 12.3% to 30.5%, consistent with the expected enrichment efficiency for histone modifications. Peak distribution analysis revealed that H2Bub1 peaks were predominantly enriched in gene body regions, with characteristic depletion at transcription start sites (TSS) and transcription termination sites (TTS), matching the established distribution pattern of H2B monoubiquitination in actively transcribed genes [32]. Notably, this gene body enrichment pattern was markedly attenuated in *ΔFgNup2*, consistent with the global reduction of H2Bub1. Using Input as background and MACS2 software with broad parameters for peak calling on IP samples. ChIP-seq analysis revealed that, compared to the wild-type PH-1, a total of 677 genes with significantly downregulated H2Bub1 enrichment were identified in *ΔFgNup2*. Intersecting these genes with the 1,732 genes significantly downregulated in the *ΔFgNup2* mutant based on our RNA-seq data yielded 91 overlapping genes (Fig 7A, B). GO functional enrichment analysis of these genes revealed their involvement in multiple important pathways, such as the MAPK signaling pathway, cell cycle, lipid metabolism, cell wall synthesis, metal ion stress response, sporulation, DON synthesis, and nucleocytoplasmic transport (Fig 7C). To validate the reliability of the ChIP-seq results, ChIP-qPCR and RT-qPCR were performed on 14 genes encompassing the above functions. The results were consistent with the sequencing data, showing significantly downregulated H2Bub1 levels and transcriptional levels for these genes in the *ΔFgNup2* strain (Fig 7D, E and S8 Fig).

**Fig 7:**
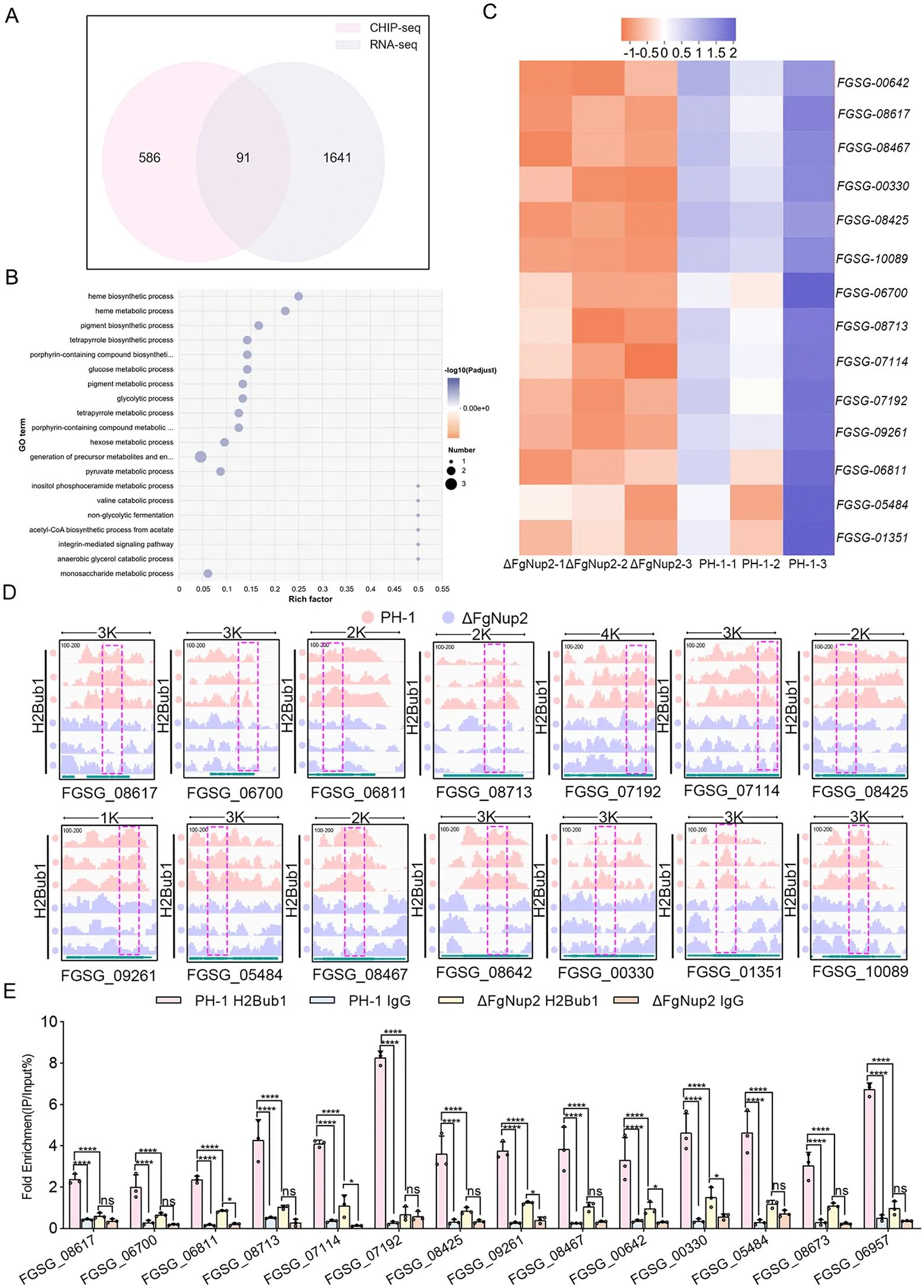
*FgNup2* regulates the growth, development, stress response and virulence of *F. graminearum* by promoting the occurrence of H2bub1. **(A)** Intersection analysis of genes with significantly downregulated H2Bub1 modifications in *ΔFgNup2* and genes significantly downregulated in the transcriptome (RNA-seq). **(B)** Heatmap of RPKM-normalised expression levels of intersection genes in PH-1 and *ΔFgNup2*. **(C)** GO functional enrichment analysis of genes intersecting ChIP-seq and RNA-seq. **(D)** Using the IGV genome browser to display H2Bub1 ChIP-seq signal profiles of representative key gene chromatin regions in wild-type PH-1 and *ΔFgNup2* strains under toxin-inducing conditions. the shown genes belong to different functional categories. **(E)** ChIP-qPCR validation of H2Bub1 modification levels at the gene loci shown in panel A in *ΔFgNup2* and PH-1, the data come from three independent repeated experiments, and significant differences are indicated by * (****P < 0.0001, *P ≤ 0.05, ns P ≥ 0.05).

### 2.8 The key Phenylalanine residue F981 in the FG-repeat region of FgNup2 regulates FgBre1 nuclear transport and virulence

FgNup2 has three functional domains: an N-terminal nucleoporin domain containing an Impα-binding domain, a middle region containing a phenylalanine-glycine (FG) repeat sequence, and a C-terminal Ran-binding domain (Fig 8A). To identify the functional sites within FgNup2 that regulate virulence in *F. graminearum*, we first fused the three domains to a GFP-tagged vector and introduced them individually into the wild-type strain PH-1/Bre1-RFP. Co-IP assays using GFP affinity magnetic beads indicated that only the FgNup2–2 (FG-repeat) domain interacted with FgBre1, suggesting the FG-repeat plays a crucial role (Fig 8B, C). Subsequently, we mutated every 8 consecutive amino acids within the 143-amino-acid FgNup2–2 segment, generating 18 mutant strains for growth and pathogenicity screening (S9 Fig). The results showed that mutation of residues 980–987 significantly reduced growth rate and pathogenicity. Further alanine-scanning mutagenesis of these 8 residues (S10 Fig) revealed that only the mutation of phenylalanine at position 981 to alanine (F981A) caused significant reductions in growth rate, pathogenicity, and DON production (Fig 8D and S11A–S11B Fig). Furthermore, we examined the nuclear import of FgBre1 in the FgNup2^F981A^ strain at 6 h post induction and found that FgBre1 exhibited a nuclear import delay phenotype consistent with that of the *ΔFgNup2* strain (Fig 8E and S11D Fig). The H2Bub1 modification level in the FgNup2^F981A^ strain was significantly lower than in the wild-type (S11E Fig). Concurrently, molecular docking simulations suggested an interaction between phenylalanine at position 981 of FgNup2 and FgBre1 (Fig 8F). Furthermore, we analyzed the sequence conservation of the F981 residue among different fungal species. The comparison results showed that F981 did not undergo substitution in a wide range of fungal groups from yeast to filamentous fungi(Fig 8G). To explore whether the F981 site is involved in regulating the interaction between FgNup2 and FgBre1, we performed Co-IP experiments in the wild-type PH-1 and the F981 point mutant strains. The results showed that in the F981 mutant strain, FgBre1 could still co-precipitate with FgNup2. However, compared with the wild type, the co-precipitation signal of FgBre1 in the mutant was significantly weakened (S11F Fig), indicating that the interaction between the two was not completely lost. The F981 site plays an important role in maintaining stable protein interactions between FgNup2 and FgBre1, but it is not an essential residue for this interaction.

**Fig 8:**
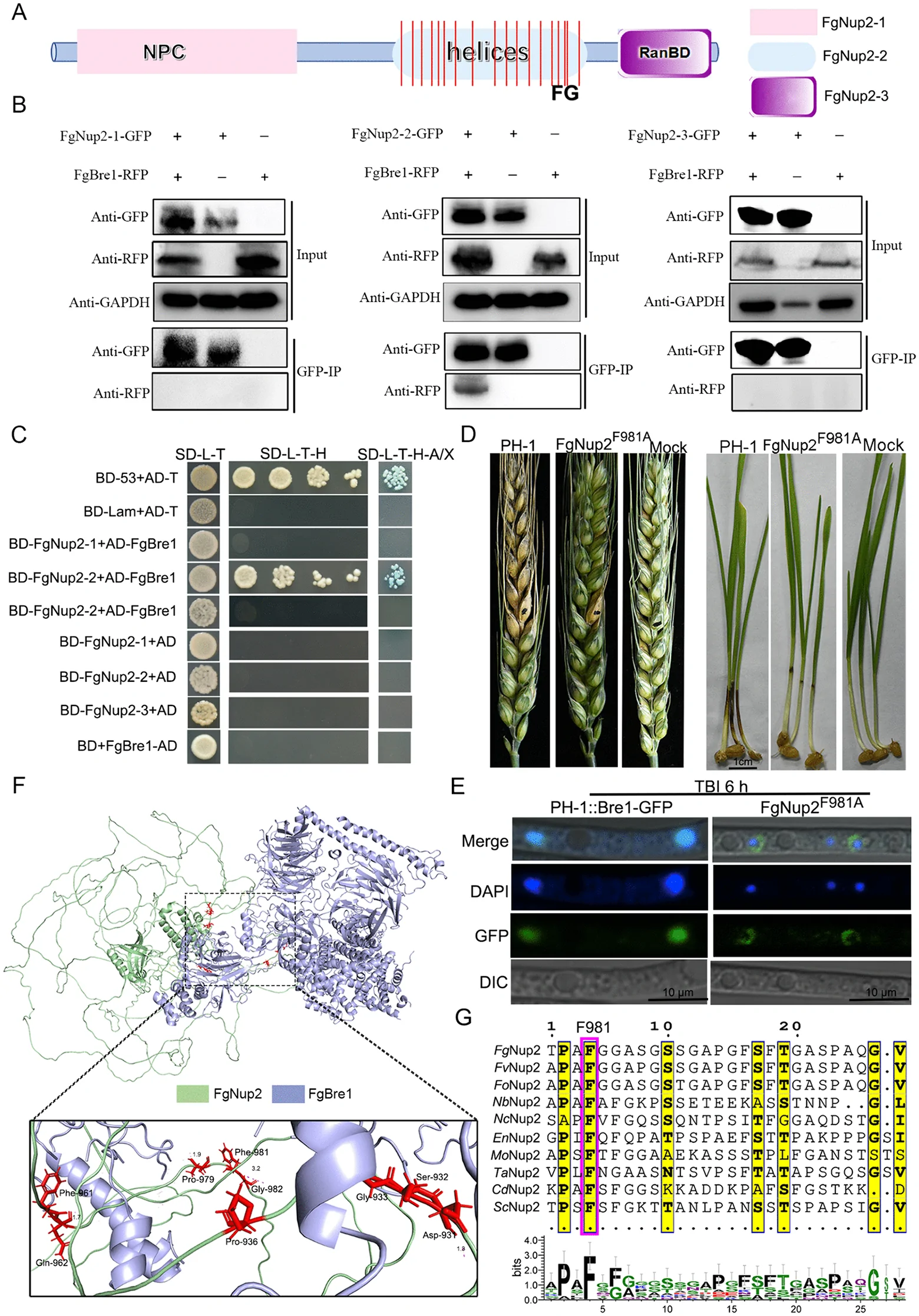
The key phenylalanine residue F981 in the FG repeat sequence of FgNup2 regulates FgBre1 nuclear transport and virulence. **(A)** Schematic diagram of the FgNup2 protein domains, showing the N-terminal Importinα binding domain, central phenylalanine-glycine repeat sequences (FG repeats), and C-terminal Ran binding domain. **(B)** Interaction detection between FgNup2-1, FgNup2-2, and FgNup2-3 domains and FgBre1 using co-immunoprecipitation (Co-IP) experiments. **(C)** Yeast two-hybrid (Y2H) experiments verification of interactions between the different domains of FgNup2 and FgBre1. **(D)** Colony morphology of wild-type PH-1 and FgNup2^F981A^ point mutant strains after 72 h of cultivation on PDA, CM, and MM media. **(E)** Comparison of disease symptoms between wild-type PH-1 and FgNup2^F981A^ strains in wheat coleoptile and spike inoculation tests. **(F)** After 6 h under toxin-inducing conditions, we used a laser confocal microscope to check the subcellular localisation of FgBre1 in the FgNup2^F981A^ point mutant. **(G)** The overall level of H2Bub1 modification in the FgNup2^F981A^ strain was detected by Western Blot. **(H)** Molecular docking simulation of the interaction between FgNup2 and FgBre1. Visualization of the molecular docking model of the FgNup2 and FgBre1 protein complex. The enlarged section of the figure shows the interaction interface between FgNup2 and FgBre1 and highlights the key residues of FgNup2.

## 3 Discussion

The pathogenic process of *F. graminearum* involves a complex gene expression regulatory network. Nucleocytoplasmic transport, a central step in this regulation, directly influences the pathogen’s ability to respond to environmental signals and host stimuli through its efficiency and selectivity [33,34]. The nuclear pore complex, the sole conduit for this transport, is modularly assembled from multiple nucleoporins [35,36]. However, the composition, classification, and specific functions of Nups in *F. graminearum* remain unknown, significantly hindering a deeper understanding of its pathogenicity mechanisms. In this study, we identified 24 FgNups in *F. graminearum*. While these proteins lack a unified conserved domain, they share specific domains within phylogenetic subgroups, consistent with their role as components of a modular supramolecular complex. This genomic analysis confirms the presence of a structurally complete and sophisticated nucleocytoplasmic transport foundation. Notably, during wheat infection, the expression of 10 FgNups, including FgNup2, was specifically upregulated, suggesting that the pathogen may remodel the NPC to support the activation of virulence-related gene programs. However, loss-of-function phenotypic analysis showed that only deletion of FgNup2 resulted in severe growth and pathogenicity defects, whereas single deletions of the other FgNup genes produced no obvious phenotypes. This prompted us to further consider the functional differentiation within the nucleoporin family. We propose that the prominent phenotype of FgNup2 reflects a division of labor among nucleoporins in terms of both structure and function. Scaffold nucleoporins that constitute the structural framework of the NPC generally participate in basal nucleocytoplasmic transport, and their deficiency often leads to global transport disruption or even lethality, making their individual contributions difficult to evaluate in phenotypic screens. In contrast, a subset of nucleoporins performs more specialized transport-regulatory functions by interacting with specific nuclear transport receptors or cargo proteins, thereby selectively modulating the nuclear import of signaling molecules or transcription factors. FgNup2 appears to belong to this latter category, and its cargoes may include regulators directly involved in conidiation and infection, resulting in pleiotropic effects on fungal development and pathogenicity. Meanwhile, functional redundancy among nucleoporins may also dilute the phenotypes of single-gene knockouts: if the function of a given member can be compensated by a paralog or another NPC component, the effect of its deletion may be masked. Our data indicate that FgNup2 participates in multiple independent pathways, including vegetative growth, conidiation, and pathogenicity, suggesting that it acts as a common regulatory node, whereas other nucleoporins may affect only specific processes or may function under particular conditions, such as developmental stages requiring large-scale gene expression reprogramming, or under environmental stresses such as oxidative stress, nutrient starvation, or exposure to different metal ions. Testing these hypotheses will require constructing multiple knockout mutants to eliminate potential redundant compensatory effects, or conducting more refined phenotypic analyses under specific stress conditions, developmental stages, or host environments.

Regulating macromolecular transport and subcellular localization is a core requirement for pathogenic microbes to adapt to their environment. In fungal pathogens, functional alterations in NPC or nuclear envelope-associated proteins often lead to defects in secondary metabolism, virulence factor secretion, and stress responses [37]. Our RNA-seq analysis revealed that the deletion of *FgNup2* in *F. graminearum* triggered extensive transcriptome reprogramming, affecting core biological processes including primary and secondary metabolism, cell cycle, and cell wall synthesis. Unlike the classic model in *S. cerevisiae*, where Nup2 is primarily involved in nuclear transport and chromatin binding [38,39]. This study discovered an interaction between FgNup2 and the histone ubiquitin ligase FgBre1. This interaction enables FgNup2 to recruit FgBre1 to target gene loci, coordinating H2B ub1 to synchronously activate transcription of relevant genes, thereby promoting rapid adaptation to the harsh environment of the infection site. The deletion of *FgNup2* likely disrupts the subcellular localization or enzymatic activity of FgBre1, altering the genome-wide H2Bub1 modification landscape and leading to widespread gene expression dysregulation. This provides a plausible explanation for the observed transcriptomic changes in the *ΔFgNup2* mutant. In plant pathogenic fungi, knockout of the E3 ligase gene responsible for H2Bub1 results in complete loss of H2Bub1, accompanied by significant reductions in hyphal growth, conidiation, and virulence [26,40]. Analysis of the PH-1::Bre1^ΔFgNup2^ strain revealed that, despite FgBre1 overexpression, the loss of FgNup2 led to phenotypic defects in vegetative growth, pathogenicity, and toxin production nearly identical to those of the *ΔFgNup2* single mutant, rather than restoring the phenotype expected from FgBre1 overexpression. It should be noted that the toxin production phenotype was evaluated in TBI medium. This medium, selected based on the systematic screening by Gardiner et al [31], is chemically defined and efficiently induces DON production, enabling the regulatory effect of a gene on DON biosynthesis to be assessed under homogeneous conditions. However, it lacks the physical and biological factors present in the host environment, such as cell wall structures and reactive oxygen species (ROS) bursts, which represents an inherent discrepancy from the actual infection condition—a limitation common to all in vitro culture systems. However, direct functional evidence is still lacking to demonstrate that forced nuclear localization of FgBre1 via an exogenous NLS can complement the *ΔFgNup2* phenotype. Therefore, it cannot be rigorously concluded that this transport mechanism is the sole or predominant pathway. Accordingly, we cautiously define FgNup2-mediated nuclear transport of FgBre1 as one of the key upstream events governing development and virulence in *F. graminearum*.

Nucleocytoplasmic transport is a critical node in gene expression regulation, with its precision directly affecting the spatiotemporal distribution and function of regulatory factors such as transcription factors and chromatin modifiers [4,5,41]. This study found that FgNup2 is a key facilitator for the rapid and efficient nuclear import of FgBre1. In the wild-type strain, trichothecene biosynthesis-inducing medium treatment triggered rapid nuclear accumulation of FgBre1 within 1 h. However, this process was significantly delayed in the *ΔFgNup2* mutant, taking up to 24 h to complete. This delayed, rather than completely blocked, phenotype suggests that FgNup2 is not the sole or absolutely essential mediator for FgBre1 nuclear import, but it is crucial for achieving rapid transcriptional reprogramming under stress conditions. Further mechanistic investigation showed that FgBre1 nuclear import depends on the classical nuclear import receptor FgImpα, with the two proteins interacting directly both in vivo and in vitro. This finding aligns with the classical nuclear import receptor-cargo recognition model. More importantly, the deletion of FgNup2 significantly weakened the interaction between FgBre1 and FgImpα, indicating that specific nucleoporins can actively participate in and stabilize the assembly or docking of nuclear transport receptor-cargo complexes. FgNup2 may act as a molecular scaffold, simultaneously binding both FgImpα and FgBre1 to promote or stabilize their interaction. Alternatively, the absence of FgNup2 might alter the local microenvironment or conformation at the cytoplasmic face or nuclear basket of the NPC, indirectly affecting the initial docking efficiency of the FgImpα-cargo complex at the pore and thus reducing the detectable steady-state interaction level. Both possibilities indicate an active regulatory role for FgNup2 in FgBre1 nuclear import, beyond merely serving as a passive channel component. Although our data demonstrate that FgNup2-mediated nuclear import of FgBre1 regulates development and pathogenicity, this pathway may not represent the sole mechanism. As the nuclear pore complex serves as the gateway for hundreds of cargo proteins, *ΔFgNup2* likely disrupts the nucleocytoplasmic trafficking of multiple transcription factors, epigenetic modifiers, and signaling components beyond FgBre1. This is consistent with the observation that *ΔFgNup2* exhibits more severe pleiotropic defects than *ΔFgBre1*, including aberrant lipid metabolism, altered stress responses, and defective sporulation, which cannot be fully attributed to impaired H2Bub1. Thus, FgNup2 may regulate fungal development and virulence through multiple coordinated pathways, with the FgBre1–H2Bub1 axis representing one major but not exclusive effector route. Systematic identification of its complete cargo spectrum will be a key direction for future research.

Crucially, this study further mapped the molecular function of FgNup2 to a key residue, F981, within its FG-repeat domain. The FgNup2^F981A^ fully recapitulated the delayed nuclear import phenotype of FgBre1 observed in the *ΔFgNup2* mutant. This directly demonstrates that the FG domain of FgNup2, particularly the F981 site, is the structural basis essential for its facilitatory function. To assess the evolutionary conservation of this residue, we performed sequence alignments of F981 across representative fungal species. The alignment revealed that F981 is invariant from yeasts to filamentous fungi, consistently maintaining a phenylalanine residue. Although FG repeat domains are generally enriched in phenylalanine–glycine dipeptides, the side-chain properties of individual residues vary considerably among species [42,43]. The invariant phenylalanine at F981 across such a broad fungal lineage suggests that its hydrophobic aromatic side chain is under strong selective constraint for Nup2 function, likely playing a direct role in nuclear transport receptor binding or the conformational maintenance of the nuclear pore complex. This conserved feature further supports the notion that F981-mediated hydrophobic interactions are a central determinant of FG network function, rather than a species-specific incidental observation. Research indicates that the function of FG-repeat domains relies on hydrophobic interactions mediated by phenylalanine residues to form a dynamic FG network, which serves as the docking and translocation platform for transport complexes [15,44]. The F981A mutation likely disrupts this key hydrophobic core, impairing the overall conformation and function of the FG domain and consequently affecting its ability to act as a scaffold or maintain the local microenvironment. While the F981A mutation highlights the importance of the FG-repeat domain, further structural and biophysical analyses are needed to determine whether FgNup2 functions as a direct scaffold or by modulating the NPC microenvironment.

In this study, we identified 24 FgNups in *F. graminearum*. The finding that 10 of them, including FgNup2, are upregulated during wheat infection suggests active remodeling of the NPC during pathogenesis. Although most individual *FgNup* deletions did not exhibit any obvious phenotype, FgNup2 is essential for growth and pathogenicity. Under stress conditions, the nucleoporin FgNup2 acts as a key driver for the rapid nuclear import of the histone ubiquitin ligase FgBre1, a process crucial for H2Bub1-mediated stress-responsive transcriptional reprogramming. This process relies on the classical nuclear import receptor FgImpα, and FgNup2 actively promotes or stabilizes the FgImpα-FgBre1 interaction—specifically through a phenylalanine residue (F981) within its FG-repeat domain—to ensure their efficient transport. These findings reveal a novel and efficient regulatory mechanism whereby a specific nucleoporin directly links nuclear transport to chromatin modification to control fungal pathogenicity. They demonstrate that specific nucleoporins in *F. graminearum* can regulate the efficient nuclear transport of key regulators, thereby deepening our understanding of fungal pathogenicity mechanisms (Fig 9).

**Fig 9:**
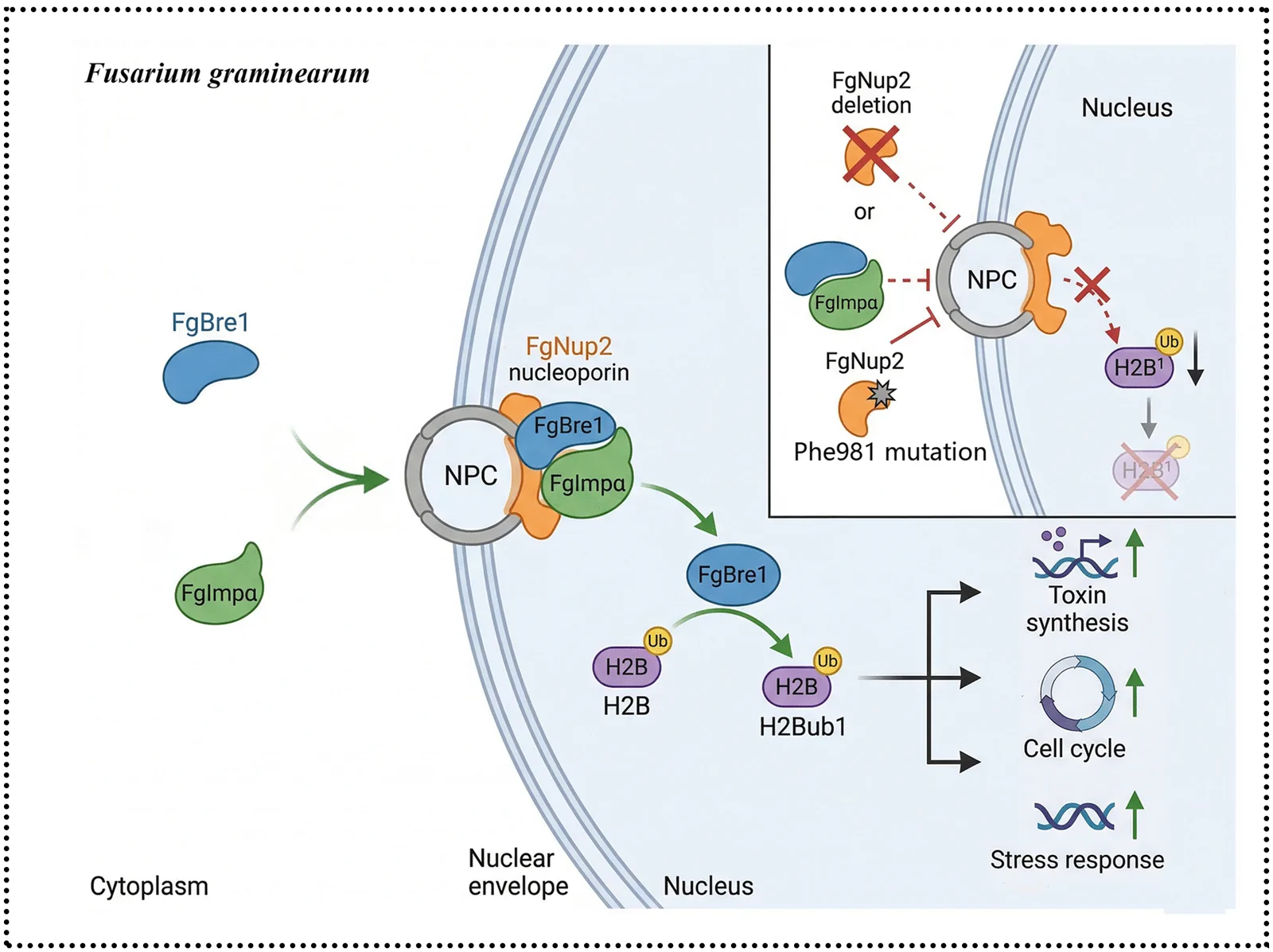
A schematic model of FgNup2, a nucleoporin in *F. graminearum*, regulating H2Bub1. FgNup2 promotes and stabilises the formation of the Fglmpa-FgBrel complex, facilitating the assembly of the FgNup2-Fglmpa-FgBrel ternary complex, which ultimately promotes the rapid nuclear transport of FgBrel under TBI-induced conditions, enhancing H2Bub1 occurrence. The occurrence of H2Bub1 can promote *F. graminearum* growth, pathogenicity, and DON toxin synthesis, which are crucial. Moreover, FgNup2 plays a critical role in this process through the key residue F981 in its FG repeat domain.

## 4 Materials and Methods

### 4.1 Fungal strains and construction of fungal deletion

The wild-type (WT) strain PH-1 (NRRL 31084) of *F. graminearum* was used as the parental strain as described previously [45]. Potato dextrose agar (PDA), minimal media (MM), complete medium (CM) were used to measure Determination of growth rates, conidiation in liquid carboxymethylcellulose (CMC) medium and trichothecene biosynthesis-inducing(TBI) medium A is used to induce *F. graminearum* to produce deoxynivalenol(DON). For the detailed recipe of the culture medium, see Table S3 in S1 Appendix.

Constructs for gene deletion of *F. graminearum* using a PEG-mediated protoplast transformation method were carried out as described previously. Briefly, fresh mycelia were treated with driselase (D9515, Sigma, MO, USA), lysozyme (S10038, Yuanye, Shanghai, China) and cellulose (S10042, Yuanye, Shanghai, China). The open reading frame (ORF) of each gene was replaced with hygromycin resistance cassette (*HPH*) [46]. Primers used to amplify the flanking sequences for each gene are listed in Table S4 in S1 Appendix. Deletion mutants were identified by PCR and Southern blot assays.

### 4.2 Phylogenetic analysis

Nucleoporin protein sequences of *F. graminearum* strain PH-1 and Saccharomyces cerevisiae strain S288C were retrieved from the NCBI database (https://www.ncbi.nlm.nih.gov/). Phylogenetic trees were constructed using MEGA-X with the maximum likelihood method based on the Jones-Taylor-Thornton substitution model. Bootstrap values were calculated from 1,000 replicates to assess branch support. The resulting tree was visualized and annotated using the iTOL web server (version 6; https://itol.embl.de/).

### 4.3 GFP and RFP fusion cassettes construction

To construct the FgNup2-GFP cassette, *FgNup2* containing native promoter region and ORF (without the stop codon) was amplified with the relevant primers in Table S4 in S1 Appendix. The resulting PCR products were co-transformed with XhoI-digested pYF11 containing a neo cassette into the yeast strain XK1–25 using the Alkali-Cation Yeast Transformation Kit (MP Biomedicals, Solon, USA) to generate the recombined FgNup2-GFP fusion vector. Subsequently, the FgNup2-GFP fusion vector was recovered from the yeast transformant using the Yeast Plasmid Kit (Solarbio, Beijing, China) and then transferred into Escherichia coli strain DH5α for amplification. Using the similar strategy, FgImpα-GFP, FgBre1-GFP and FgBre1-RFP, FgNup2–1-GFP, FgNup2–2-GFP and FgNup2–3-GFP fusion cassettes were constructed. The FgNup2-GFP cassettes was respectively transformed into *ΔFgNup2* to acquire complemented strains. Cassette pairs FgNup2-GFP + FgBre1-RFP, FgImpα-GFP + FgBre1-RFP, FgNup2–1-GFP + FgBre1-RFP, FgNup2–2-GFP + FgBre1-RFP, and FgNup2–3-GFP + FgBre1-RFP were transformed into PH-1 for Co-IP assay and co-localization assay.

### 4.4 Determination of stress sensitivity, virulence and DON production

To determine sensitivity to various stresses, 5 mm mycelial plugs of each strain taken from a 3-day-old colony edge were inoculated on PDA supplemented without/with each stress agent (0.2 g·L^−1^ Congo red, 0.01% SDS, 1 mol·L^−1^ KCl, 0.3 mol·L^−1^ CaCl_2_, 0.2 mol·L^−1^ MgCl_2_ and 0.1 mol·L^−1^ LiCl), Add the stress agent to the sterilised PDA medium cooled to about 55°C, mix well and pour into plates. Use a puncher to take a 5 mm diameter fungal disc from the colony edge and inoculate it in the centre of a PDA plate containing the stress agent, then incubated at 25°C for 3 d in the dark. Each experiment was repeated three times independently.

For evaluating virulence on wheat spikelets, a 10 μL aliquot of conidial suspension (10^5^ conidia/ml) was injected into a floret in the central section spikelet of single flowering wheat heads of susceptible wheat (Triticum aestivum) cultivar Jimai 22. There were 20 replicates for each strain. At 15 d after inoculation, infected spikelets in each inoculated wheat head were recorded. When cultivating coleoptiles, the seeds are first placed in a petri dish lined with moist filter paper in the dark at 25°C for 24 h. Then, the coleoptiles are transferred to sterile transparent plastic containers and incubated at 16°C under a 12 h light/12 h dark cycle for 48 h to induce wheat seed germination. A sterile scalpel is used to make a 2 mm incision at the tip of the coleoptile, and 5 μL of conidial suspension (10^5^ spores/mL) is directly inoculated on the wound. After inoculation, the seedlings are grown in a growth chamber at 25°C and 95% humidity for 10 d, after which the lesion length is measured. Twenty replicates were used for each strain. Each virulence experiment was repeated three times.

Take 5 strains of *F. graminearum* and cultivate them in YEPD medium at 25°C and 180 rpm in a shaking incubator for 16 h to prepare mycelia. After filtering the mycelia, rinse them with sterile water and transfer them to a conical flask containing 30 mL of TBI medium. Cultivate them in the dark at 25°C and 180 rpm for 48 h. After the cultivation is completed, filter, wash the mycelia with sterile water, and transfer them to a pre-weighted aluminum foil dish. Dry them in a 55°C oven for 48 hours. The dried mycelia are used for dry weight determination. Collect the filtrate and dilute it 100 times before directly using the DON quantitative kit Wis008 (Wise Science, Zhenjiang, China) to determine the DON toxin content.

### 4.5 Western blotting and co-immunoprecipitation (Co-IP) assays

Fresh mycelia (500 mg) from each fungus were ground into a fine powder and suspended in 1 mL of extraction buffer containing 1% protease inhibitor. After homogenising with a vortex mixer, the lysate was centrifuged at 10,000 × g for 20 minutes at 4°C to extract total protein from *F. graminearum*. The resulting proteins were separated by 10% sodium dodecyl sulfate-polyacrylamide gel electrophoresis (SDS-PAGE) and transferred to Immobilon-P transfer membrane (Millipore, Billerica, MA, USA). GFP, RFP and Flag-tagged proteins were detected with monoclonal anti-GFP (ab32146 and ab124754 Cambridge, UK) and anti-Flag (A9044, Sigma) antibodies, respectively. H2Bub1, level was detected with the antibody Cell Signalling 5546. H3 and GAPDH levels was detected by anti-H3 antibodies (Huabio EM1306 and EM1101) for the protein loading reference. For Co-IP assays, total proteins were extracted and incubated with anti-GFP agarose (KTSM1301, Shenzhen, China). Proteins eluted from agarose were detected by protein Western blotting as described above.

### 4.6 Yeast two-hybrid (Y2H) assays

To construct plasmids for Y2H analyses, cDNA was used to clone the coding sequence of each tested gene with primers in Table S4 in S1 Appendix. The cDNA fragment was inserted into the yeast GAL4-binding domain vector pGBKT7 and GAL4-activation domain vector pGADT7 (Clontech, Mountain View, CA, USA), respectively. The pairs of Y2H plasmids were transformed into the *S. cerevisiae* strain Y2H Gold with the lithium acetate/single-stranded DNA/polyethylene glycol transformation protocol. The pair of pGBKT7–53 and pGADT7 plasmids was used as positive control, and the pair of pGBKT7-Lam and pGADT7 plasmids was used as negative control. After cultured on minimal synthetic defined (SD) medium lacking Leu and Trp at 30°C for 3 d, the transformants were transferred to SD lacking His, Leu, Trp and Ade to assess protein–protein interaction.

### 4.7 GST pull-down assay

To express protein, the coding sequences of FgNup2 and FgBre1 were amplified from cDNA, and then, the fragments were inserted into pGEX-6p-1 and pET28a, respectively, to generate constructs for GST-tagged and His-tagged proteins. These plasmids were then transformed into the *E. coli* strain *BL21*. The resulting GST-tagged proteins were purified by the GST agarose beads (C600031; BBI, Shanghai, China) and eluted by reduced glutathione solution, and the His-tagged proteins were purified by Ni sepharose beads (20503ES10; Yeasen Biotechnology Co. Ltd, Shanghai, China) and eluted by imidazole. To conduct the GST pull-down assay, purified FgBre1-GST and FgNup2-His were mixed and incubated at 4°C for 2 h, and then, GST agarose beads were added and incubated at 4°C for 2 h. The resulting protein complexes were washed ten times with 1 × TBS to avoid nonspecific binding proteins and then eluted by 5 × SDS buffer and boiled for 10 min. Finally, protein samples were analyzed by Western blot using the mouse monoclonal anti-GST (LF305, EpizymeBio Co. Ltd) and the mouse monoclonal anti-His (LF307, EpizymeBio Co. Ltd) antibodies.

### 4.8 Microscopic examination

Conidia were stained with Calcofluor white to observe conidial morphology. The nuclear dye DAPI was used to determine FgNup2, FgBre1and FgImpα localization. To examine formation of DON toxisome, the Tri1-GFP fusion cassette was constructed and transformed into PH-1, ∆FgNup2, respectively. The resulting strains were cultured in TBI medium at 25°C for 1 h-48 h in a shaker at 180 rpm before microscopic examination. The following parameter sets of the confocal microscopy (Nikon, Tokyo, Japan). were used: The laser excitation wavelength was set at 488 nm for green fluorescence, at 405 nm for DAPI (blue fluorescence) and at 561 nm for RFP (red fluorescence). The intensity of fluorescence was acquired using the ImageJ software.

### 4.9 Bimolecular fluorescence complementation (BiFC) assay

The FgBre1-cYFP and FgNup2-nYFP fusion constructs were generated by cloning the related fragments into pHZ68 and pHZ65 vectors, respectively. FgBre1-cYFP and FgNup2-nYFP constructs were co-transformed into PH-1. In addition, construct pairs FgBre1-cYFP and FgNup2-nYFP and cYFP were used as negative controls. Transformants resistant to both hygromycin and zeocin were isolated and confirmed by PCR. Then the YFP signals were examined using confocal microscope (Nikon, Tokyo, Japan).

### 4.10 Affinity capture-mass spectrometry analysis

FgNup2 was tagged with GFP and transferred into the FgNup2 deletion mutant. The resulting transformant was used for protein extraction. After protein extraction, the supernatant was transferred to a sterilized tube. An aliquot of 25 μL of GFP-trap agarose beads was added to capture FgNup2-GFP interacting proteins, following the manufacturer’s instructions. After incubation at 4°C overnight, the agarose beads were washed three times with 1 mL of TBS (20 mM Tris–HCl, 500 mM NaCl, pH 7.5).

Proteins bound to the beads were then boiled with 50 μL TBS supplemented with 10 μL 10% SDS. After centrifugation at 5000g for 5 min at 4°C, the supernatant was digested with trypsin, and tryptic peptides were analyzed by Shanghai MeijiBio Technology Co., Ltd. using mass spectrometry. The false discovery rate (FDR) was used to determine relative protein confidence. The peptide-to-spectrum matches (PSMs) were quantified to determine relative protein abundance. Candidate interacting proteins were filtered based on high confidence (≤1% FDR) and reproducible presence across samples (PSM>5). Two biological replicates were conducted for the strain.

### 4.11 RNA extraction and qRT-PCR

Total RNA was isolated from hyphae collected in TBI medium using the RNAiso reagent (TaKaRa, Dalian, China). A reverse transcription kit was used for reverse transcription to generate complementary cDNA (Vazyme, Nanjing, China). The primer pairs used are shown in Table S4 in S1 Appendix. The actin gene of *F. graminearum* was used as the reference. Finally, the 2^−ΔΔCT^ method was used to analyze the data.

### 4.12 Chromatin immunoprecipitation (ChIP)-seq and ChIP-qPCR analyses

PH-1 and the *FgNup2* deletion mutant fresh mycelia were cross-linked with 1% formaldehyde for 15 min and then stopped with 125 mM glycine. The cultures were ground with liquid nitrogen and resuspended in lysis buffer (250 mM HEPES [pH 7.5], 150 mM NaCl, 1 mM EDTA, 1% Triton, 0.1% deoxycholate, 10 mM DTT) and 1% protease inhibitor. The DNA was sheared into~300 bp fragments with 20 pulses of 10 s with 20 s of resting at 35% amplitude (Qsonica*sonicator, Q125, Branson, USA). After centrifugation, the supernatant was diluted with 10 × ChIP dilution buffer (1.1% Triton X-100, 1.2 mM EDTA, 16.7 mM Tris–HCl [pH 8.0], 167 mM NaCl). Samples were pre-washed with 20 mL of protein A agarose (sc-2001, Santa Cruz, CA, USA) for 1 h at 4°C. Then immunoprecipitation was performed using a monoclonal anti-H2Bub1 (Cell Signalling 5546). A mock sample was incubated with anti-IgG antibody (MA1–10406, Invitrogen-Thermo Fisher Scientific, Waltham, MA, USA).

DNA was immunoprecipitated with ethanol after washing, eluting, reversing the cross-linking, and digesting with proteinase K. Further, ChIP-enriched DNA with a monoclonal anti-H2Bub1 antibody was sequenced on an Illumina NovaSeq 6000.

Different peak analysis was based on the fold enrichment of peaks of different experiments. A peak was determined as different peak when the odds ratio between two groups was more than 2. Using the same method, genes associated with different peaks were identified and also do GO and KEGG enrichment analysis. ChIP-enriched DNA with a monoclonal anti-GFP antibody were used for quantitative PCR analysis using SYBR green I fluorescent dye detection with the relative primers. The relative enrichment of each gene was determined by quantitative PCR and calculated by normalizing the value of the immunoprecipitated sample with that of the input.

### 4.13 Construction of a Fusion Expression Vector Containing Nuclear Localization Signal/Nuclear Export Signal Elements

In this study, the nuclear localization signal (NLS) sequence used was PKKKRKV. To enhance nuclear import efficiency, it was constructed as a triple tandem repeat (PKKKRKV-PKKKRKV-PKKKRKV). The nuclear export signal (NES) element was LQLPPLERLTL, which conforms to the conserved hydrophobic residue arrangement pattern (LxxLxL or LxxxLxL) of classical nuclear export signals [47]. The above sequences were synthesized by Sangon Biotech (Shanghai) Co., Ltd. and subsequently ligated into a eukaryotic expression vector containing a 3 × Flag tag via PCR or restriction enzyme digestion sites, allowing fusion expression of the target sequence with the 3 × Flag tag. The recombinant plasmid was verified by restriction enzyme digestion and DNA sequencing before being used in subsequent experiments.

### 4.14 Quantification and statistical analysis

Statistical analysis was performed using GraphPad Prism (v8.0) and ImageJ 1.53e software. Two-tailed Student’s t test was used for two-group comparisons. One-way ANOVA followed by Tukey post hoc test and two-way ANOVA followed by Tukey post hoc test were used for multiple comparisons.

## Supporting information

S1 File Rawgel. (PDF)

S2 File Raw data. (XLSX)

S1 Appendix S1 Table. Information on the Nups in *F. graminearum.* S2 Table. Information on the Nups in *N.crassa.* S3 Table. Culture medium formula. S4 Table. Related primer sequences. (DOCX)

S1 Fig Schematic representation of the predicted domain features of *F. graminearum* Nups. Different coloured blocks represent different conserved protein domains, and the black line represents the number of amino acids.
(TIF)

S2 Fig Analysis of *FgNups* expression levels during wheat infection from 0-7 d. (TIF)

S3 Fig Specific upregulation and knockout of FgNups genes in *F. graminearum.* (A) PCR identification of gene knockouts *ΔFgNup2*, *ΔFgNup53*, *ΔFgNup133*, *ΔFgSeh1*, *ΔFgSec13*, *ΔFgNup192*, *ΔFgNup82*, *ΔFgPom34*, *ΔFgPom152* and *ΔFgNic96*. (B) Southern blot verification of the above mutants.
(TIF)

S4 Fig Heatmap of RPKM-normalised transcripts, depicting 3807 differentially expressed genes from RNA-seq. (TIF)

S5 Fig In *ΔFgNup2* and PH-1, the transcriptional expression level of the FgBre1 gene was detected by RT-qPCR. (TIF)

S6 Fig Subcellular localization of FgBre1 under infection conditions. (A) Schematic representation of the predicted domain features of FgBre1. Different coloured blocks represent different conserved protein domains, and the black line represents the number of amino acids. (B) Fluorescence stereomicroscopy showing the localization of Bre1-GFP in PH-1 and *ΔFgNup2* during infection. (C) Confocal microscopy images depicting the subcellular distribution of Bre1-GFP in hyphae of PH-1 and *ΔFgNup2* at the infection stage.
(TIF)

S7 Fig Quality control and peak distribution of ChIP-seq data. (A) Principal component analysis of all ChIP-seq and Input samples. (B) Cumulative coverage percentage distribution chart of reads. (C) Viewing the distribution map of the reads gene region, the horizontal axis represents each region of the gene, and the vertical axis represents the enrichment intensity of the reads. ChIP data usually show binding peaks near the TSS (±2 kb of TSS) for all samples. (D) Aggregate peak density profiles across normalized gene regions for PH-1 and *ΔFgNup2*. (E) Peak length distribution density curves for PH-1 and *ΔFgNup2*.
(TIF)

S8 Fig RT-qPCR detection of transcriptional expression levels of the genes shown in panel A in *ΔFgNup2* and PH-1. (TIF)

S9 Fig Identification of key functional regions within the FG repeat sequence of FgNup2 protein. Schematic diagram of the systematic deletion mutation strategy of FgNup2–2 domain. 143-amino acid region was deleted in units of 8 consecutive amino acids, constructing a total of 18 deletion fragments. Each deletion mutant vector was transformed into the *ΔFgNup2* mutant to obtain complementary strains *Δ1–Δ18*.
(TIF)

S10 Fig Identification of key single amino acid sites in the FG repeat sequence of the FgNup2 protein. Schematic diagram of the strategy for alanine single-point mutation at amino acids 980–987 of FgNup2. Colony morphology of point mutation complementation strains on PDA plates after 72 h of growth.
(TIF)

S11 Fig (A) Colony morphology of wild-type PH-1 and FgNup2^F981A^ point mutant strains after 72 h of cultivation on PDA, CM, and MM media. (B) Statistical analysis of lesion lengths of PH-1 and FgNup2^F981A^ in the wheat coleoptile inoculation test. (C) Measurement of DON toxin production by the two strains under toxin-inducing conditions. The data come from three independent repeated experiments, and significant differences are indicated by * (****P ≤ 0.0001). (D) 6 h after induction, the protein distribution of FgBre1 in the nuclear and cytoplasmic fractions of the FgNup2^F981A^ strain was detected by Western Blot. (E) The overall level of H2Bub1 modification in the FgNup2^F981A^ strain was detected by Western Blot. (F) Co-IP experiments were used to detect the interaction between FgBre1 and FgNup2 in PH-1 and *ΔFgNup2* strains.
(TIF)
