# Supplementary material for: FgNup2 regulates nuclear import of the histone H2B monoubiquitination enzyme by stabilizing the FgImpα-FgBre1 complex to mediate pathogenicity in Fusarium graminearum
Source: PLoS Pathog. 2026 Sep 15;22(9):e1014577. doi: 10.1371/journal.ppat.1014577 (PMC13619055; doi:10.1371/journal.ppat.1014577)
Supplement: Supplementary file 1 [file ppat.1014577.s001.pdf]

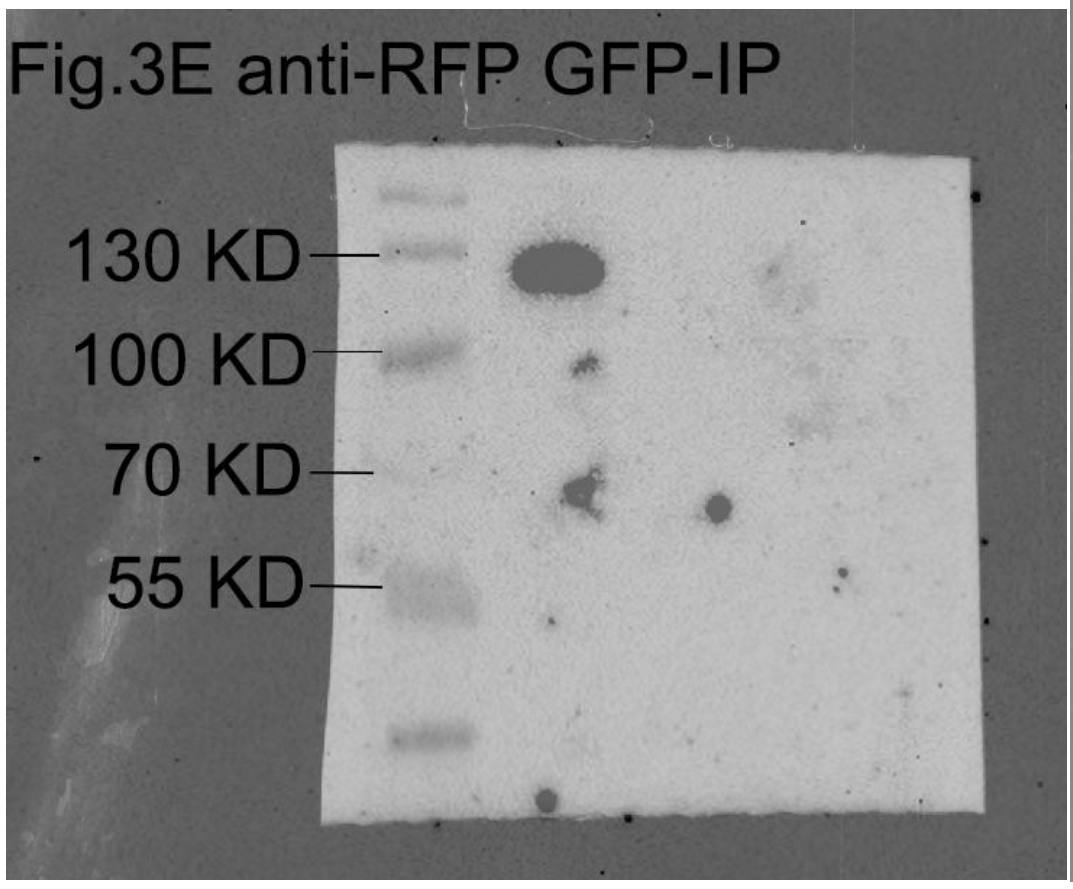
Fig.3E anti-RFP GFP-IP
130 KD
100 KD
70 KD
55 KD

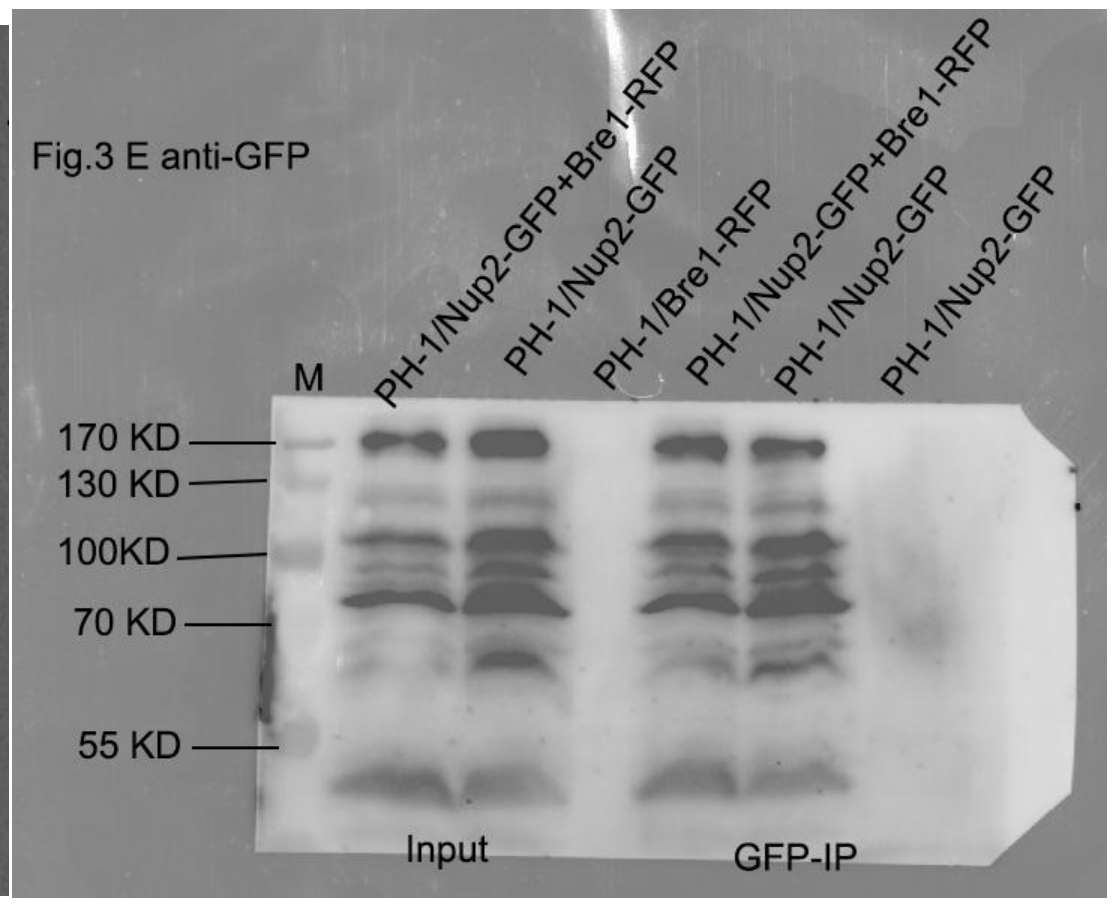
Fig.3 E anti-GFP
M
PH-1/Nup2-GFP+Bre1-RFP
PH-1/Nup2-GFP
PH-1/Bre1-RFP
PH-1/Nup2-GFP+Bre1-RFP
PH-1/Nup2-GFP
PH-1/Nup2-GFP
170 KD
130 KD
100KD
70 KD
55 KD
Input
GFP-IP

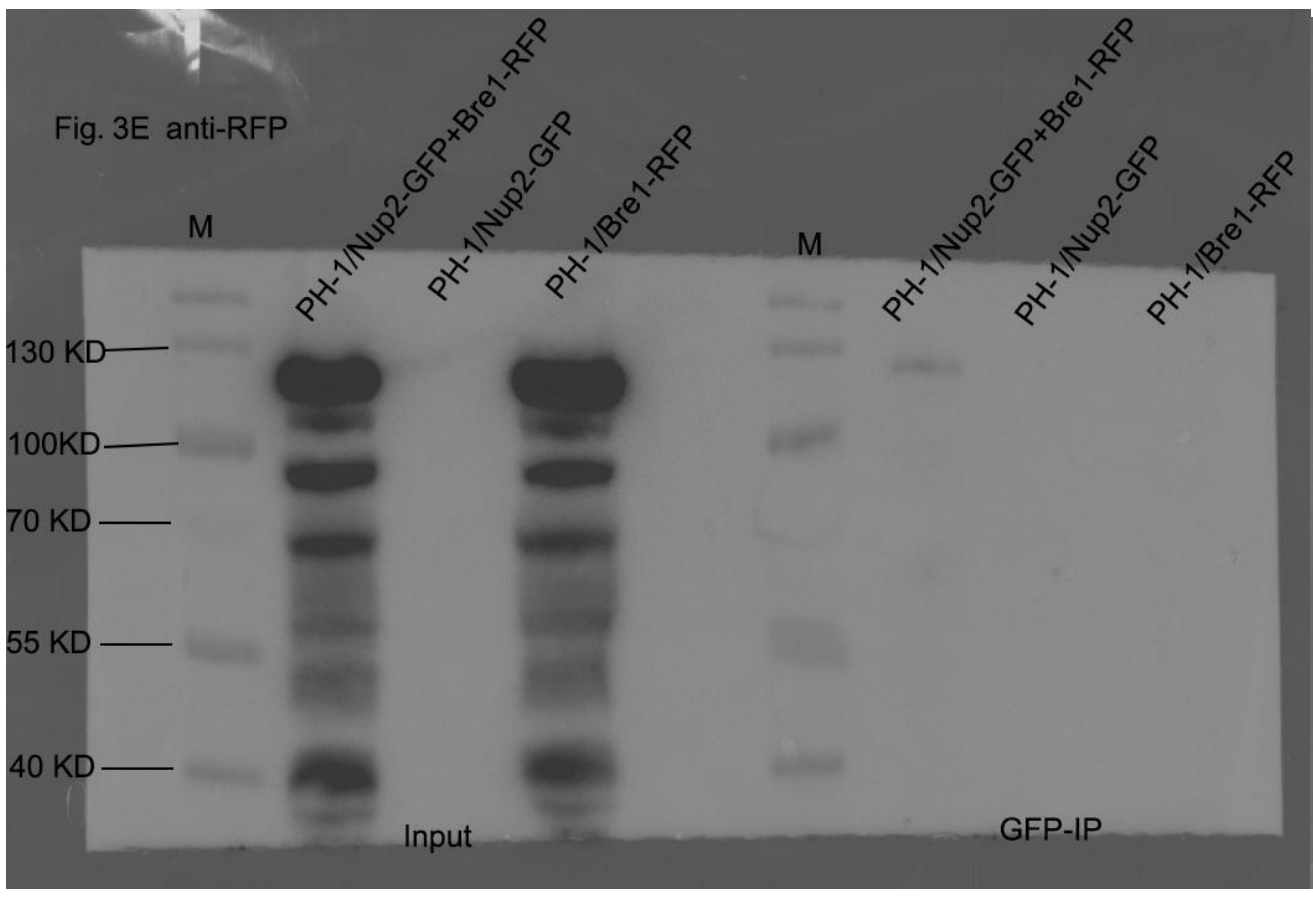
Fig. 3E anti-RFP
M
PH-1/Nup2-GFP+Bre1-RFP
PH-1/Nup2-GFP
PH-1/Bre1-RFP
M
PH-1/Nup2-GFP+Bre1-RFP
PH-1/Nup2-GFP
PH-1/Bre1-RFP
130 KD
100KD
70 KD
55 KD
40 KD
Input
GFP-IP

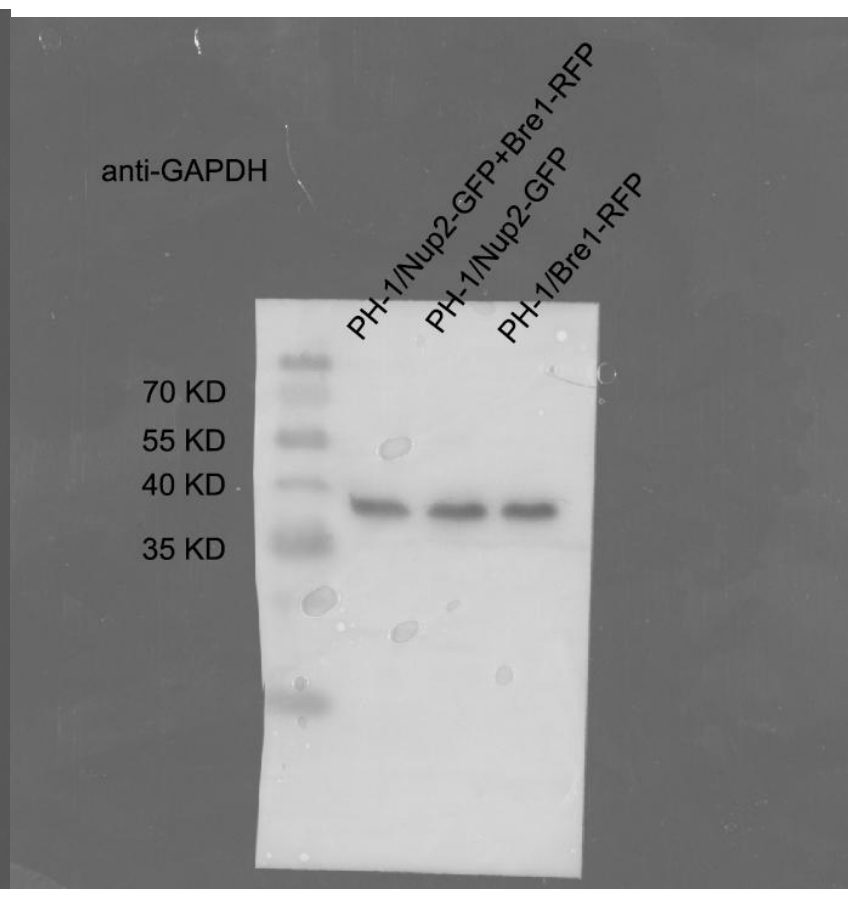
anti-GAPDH
PH-1/Nup2-GFP+Bre1-RFP
PH-1/Nup2-GFP
PH-1/Bre1-RFP
70 KD
55 KD
40 KD
35 KD

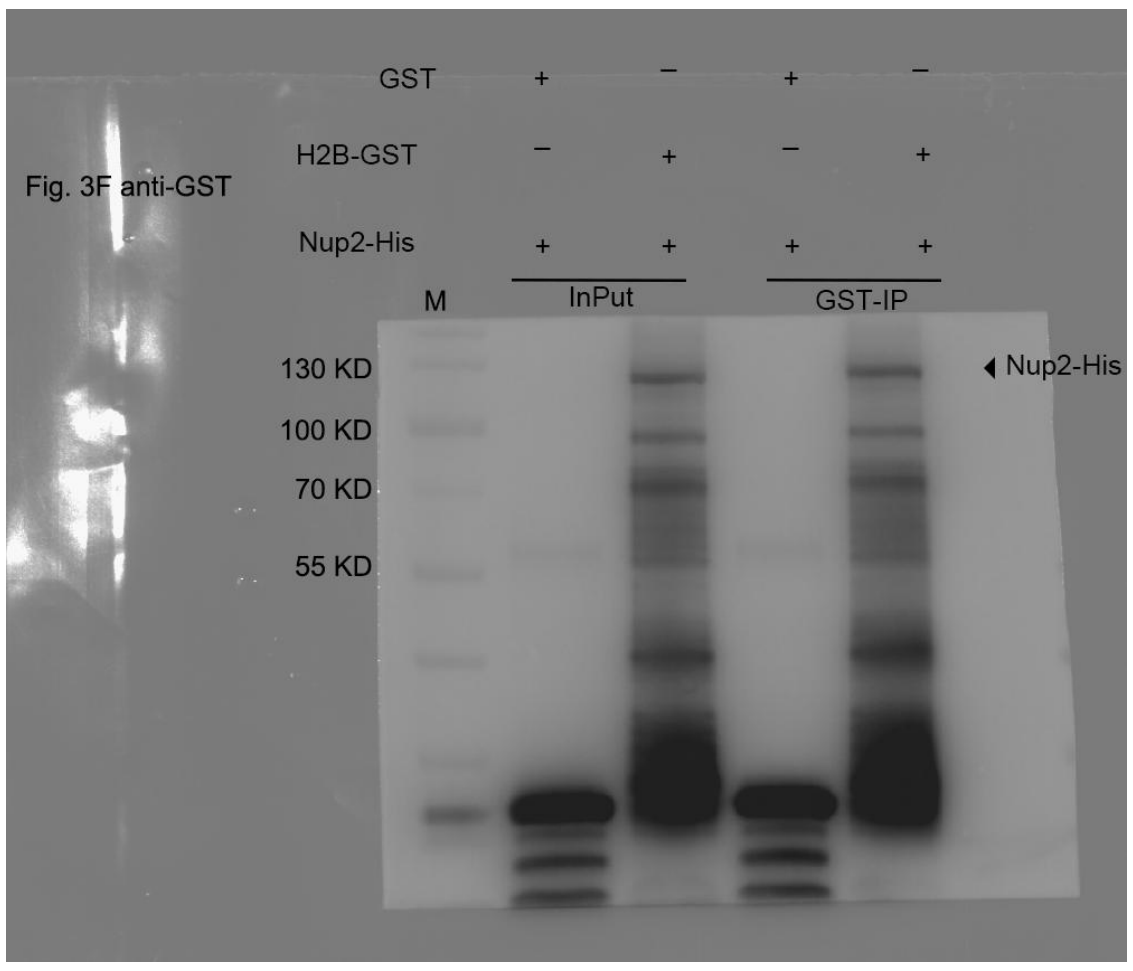
Fig. 3F anti-GST
GST + − + −
H2B-GST − + − +
Nup2-His + + + +
M
InPut
GST-IP
130 KD
100 KD
70 KD
55 KD
Nup2-His

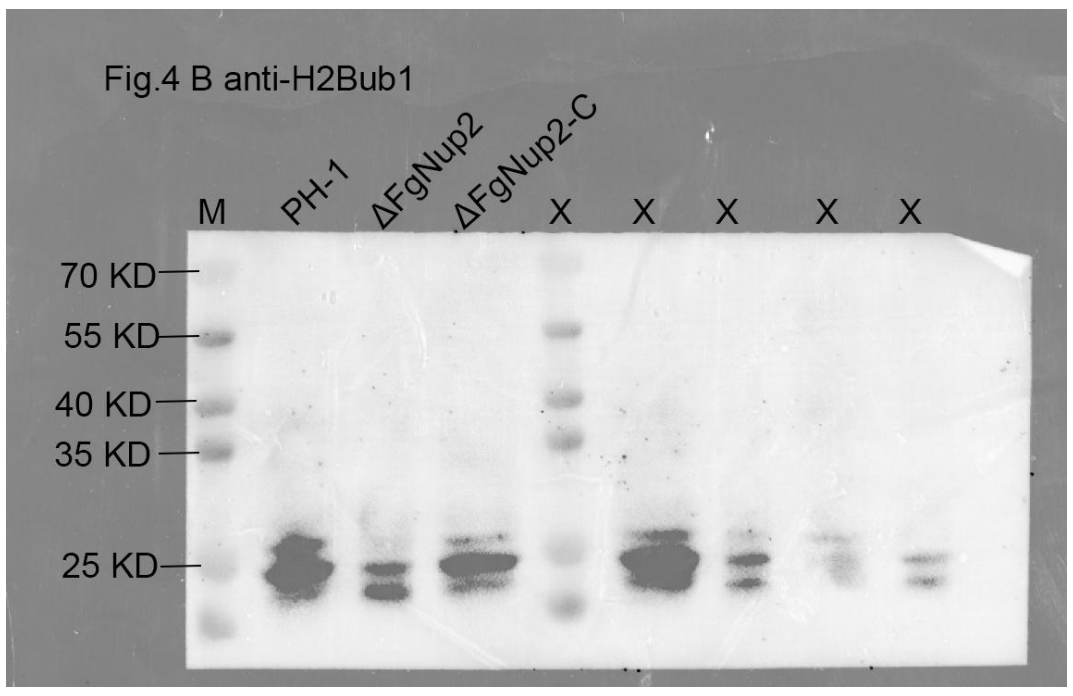
Fig.4 B anti-H2Bub1
M
PH-1
ΔFgNup2
ΔFgNup2-C
X
X
X
X
X
70 KD
55 KD
40 KD
35 KD
25 KD

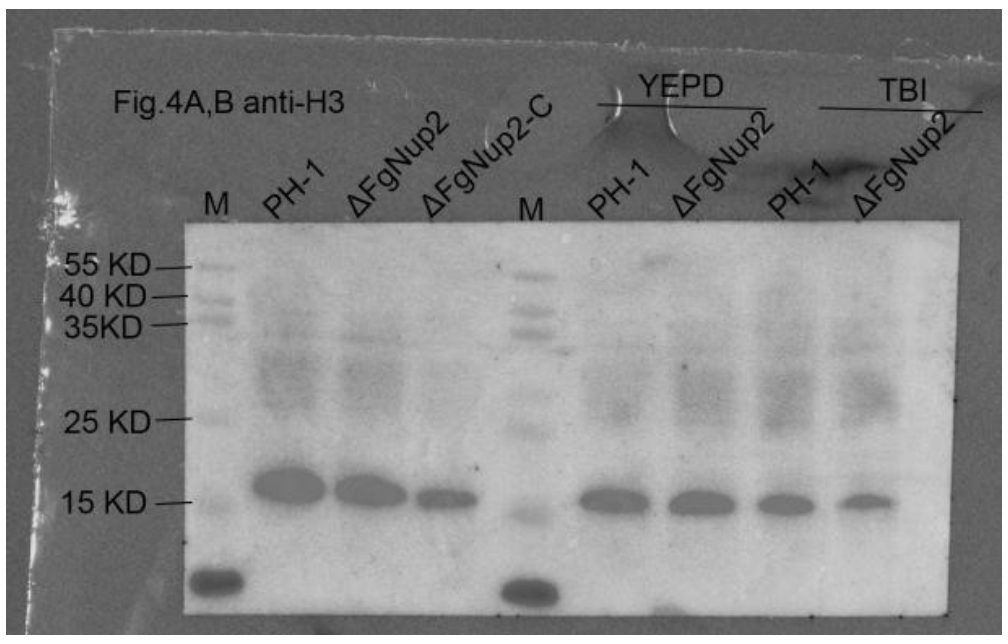
Fig.4A,B anti-H3
YEPD
TBI
M
PH-1
ΔFgNup2
ΔFgNup2-C
M
PH-1
ΔFgNup2
PH-1
ΔFgNup2
55 KD
40 KD
35KD
25 KD
15 KD

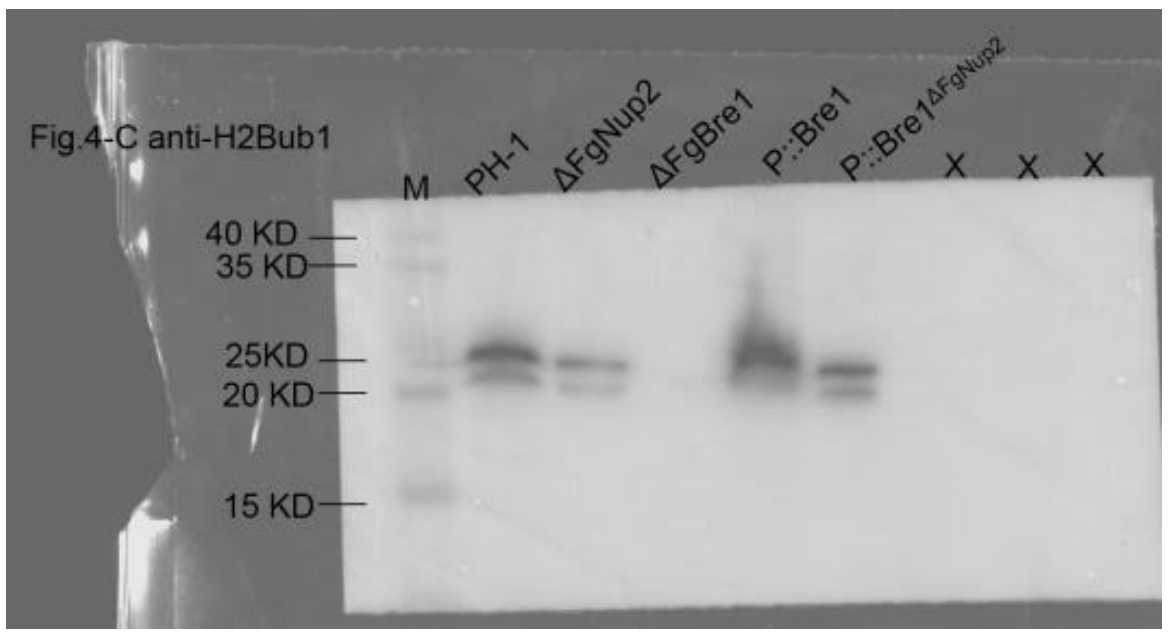
Fig.4-C anti-H2Bub1
M
PH-1
ΔFgNup2
ΔFgBre1
P::Bre1
P::Bre1ΔFgNup2
X
X
X
40 KD
35 KD
25KD
20 KD
15 KD

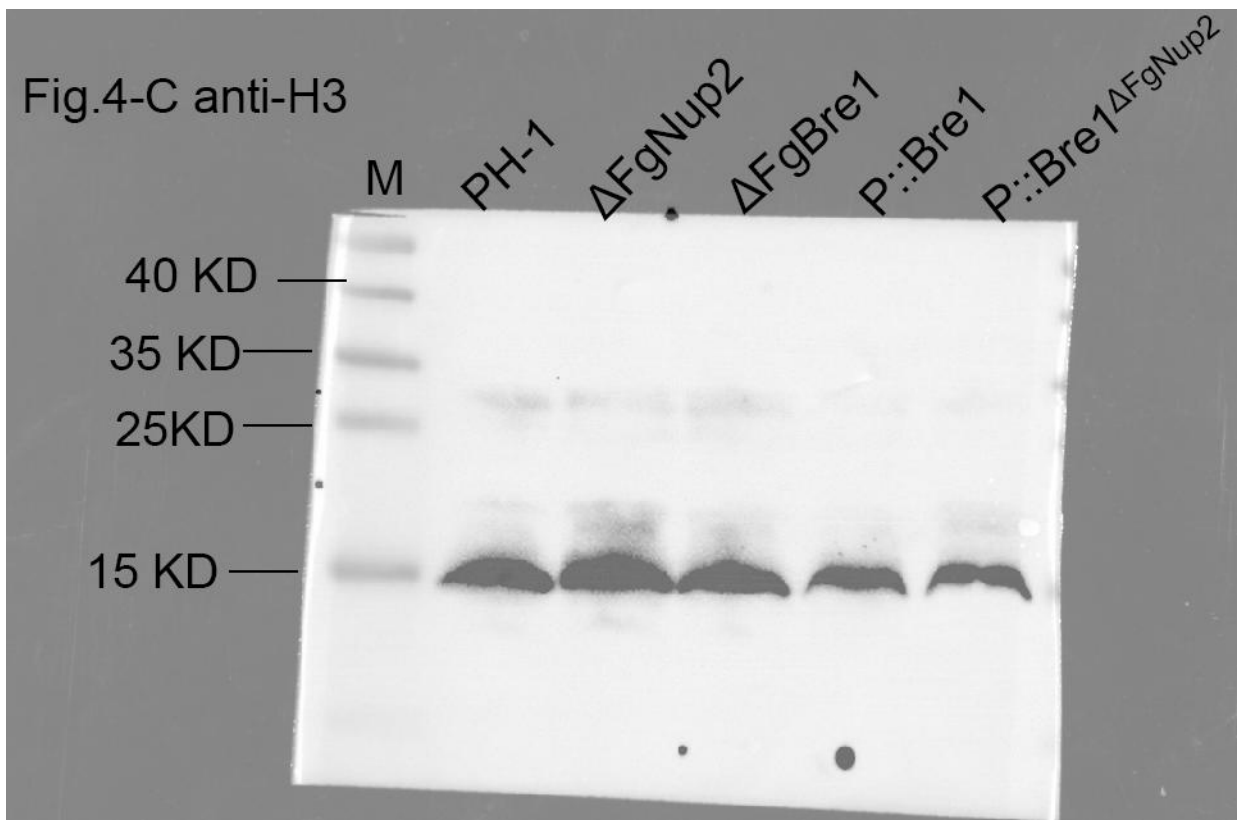
Fig.4-C anti-H3
M
PH-1
ΔFgNup2
ΔFgBre1
P::Bre1
P::Bre1ΔFgNup2
40 KD
35 KD
25KD
15 KD

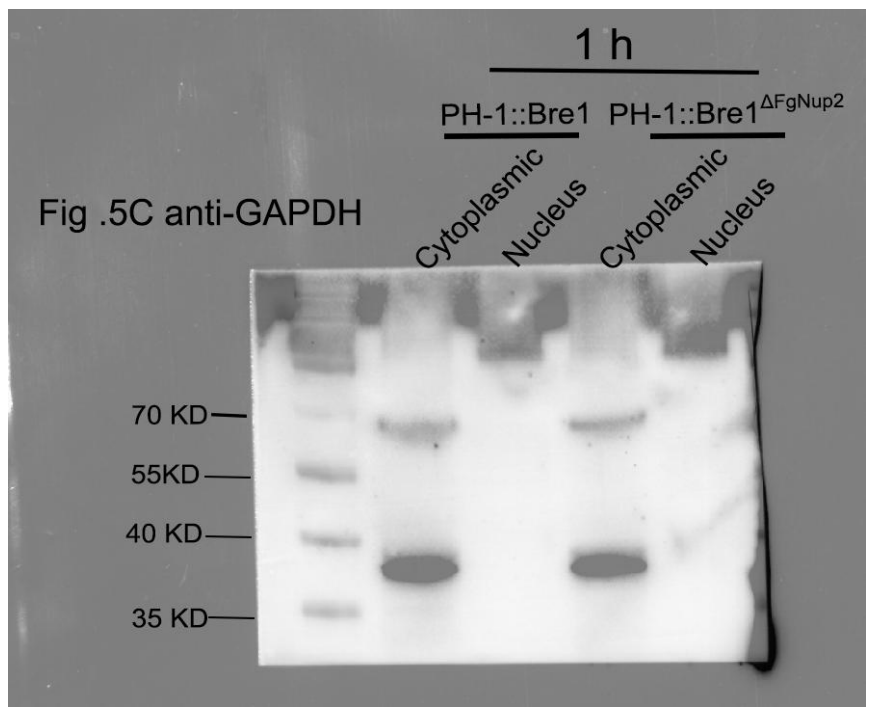
1 h
PH-1::Bre1
PH-1::Bre1ΔFgNup2
Fig .5C anti-GAPDH
Cytoplasmic
Nucleus
Cytoplasmic
Nucleus
70 KD
55KD
40 KD
35 KD

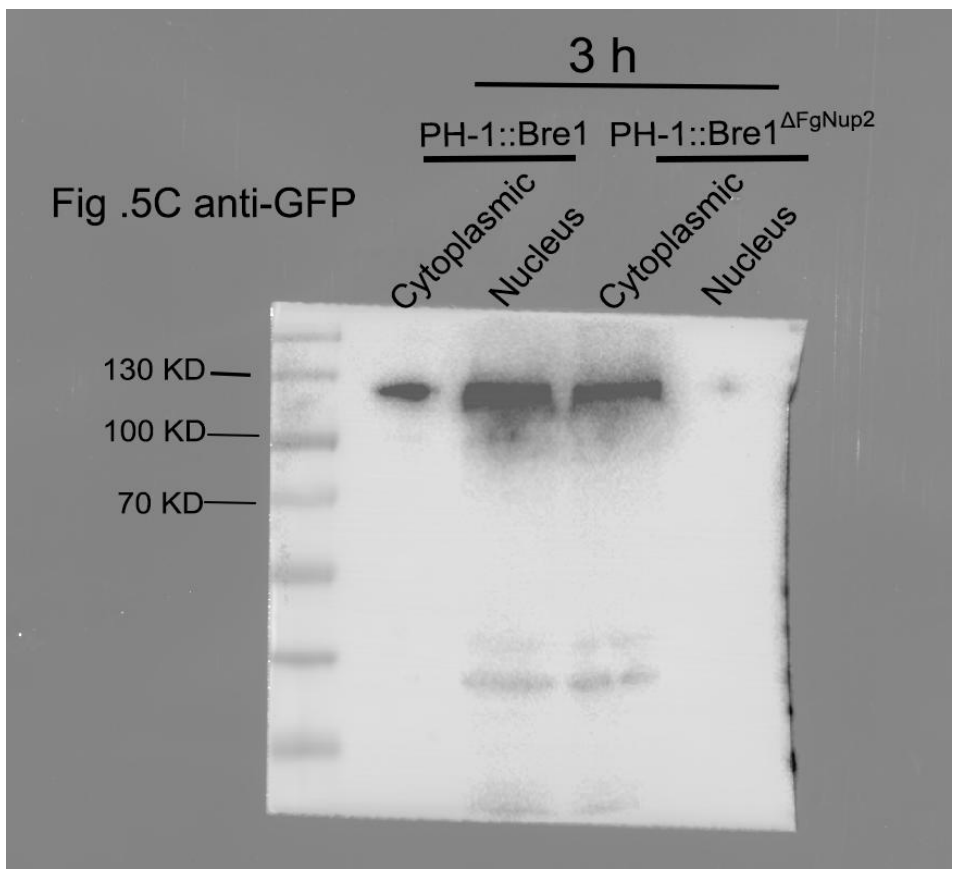
3 h
PH-1::Bre1
PH-1::Bre1ΔFgNup2
Fig .5C anti-GFP
Cytoplasmic
Nucleus
Cytoplasmic
Nucleus
130 KD
100 KD
70 KD

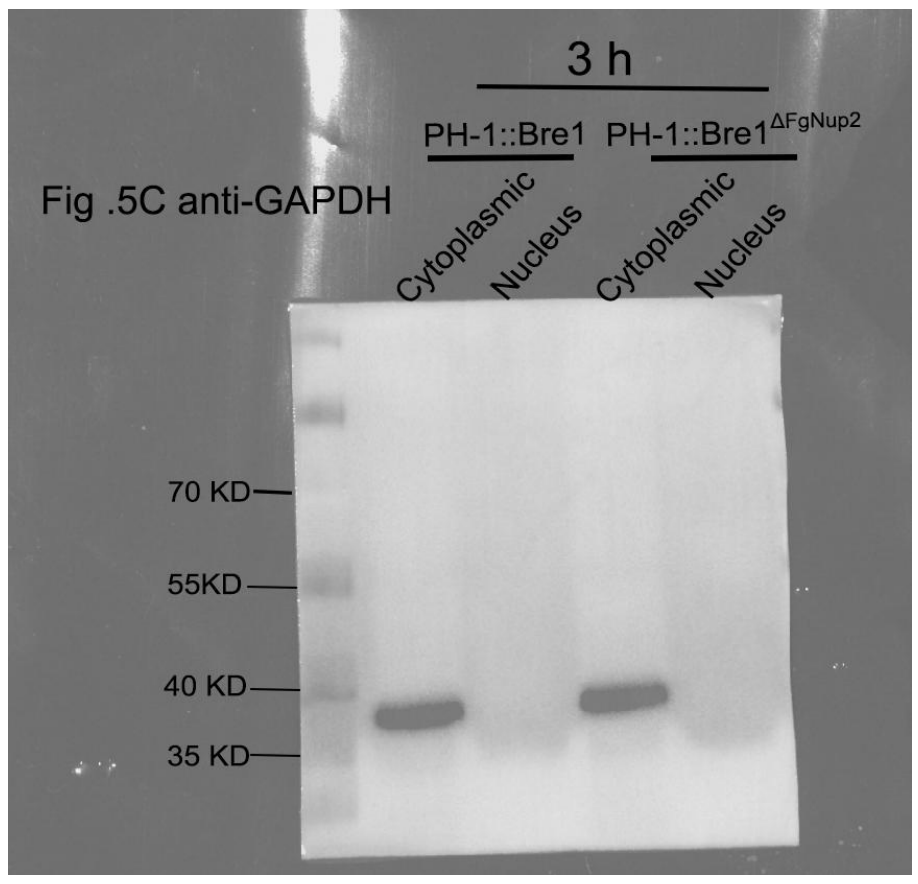
3 h
PH-1::Bre1
PH-1::Bre1ΔFgNup2
Fig .5C anti-GAPDH
Cytoplasmic
Nucleus
Cytoplasmic
Nucleus
70 KD
55KD
40 KD
35 KD

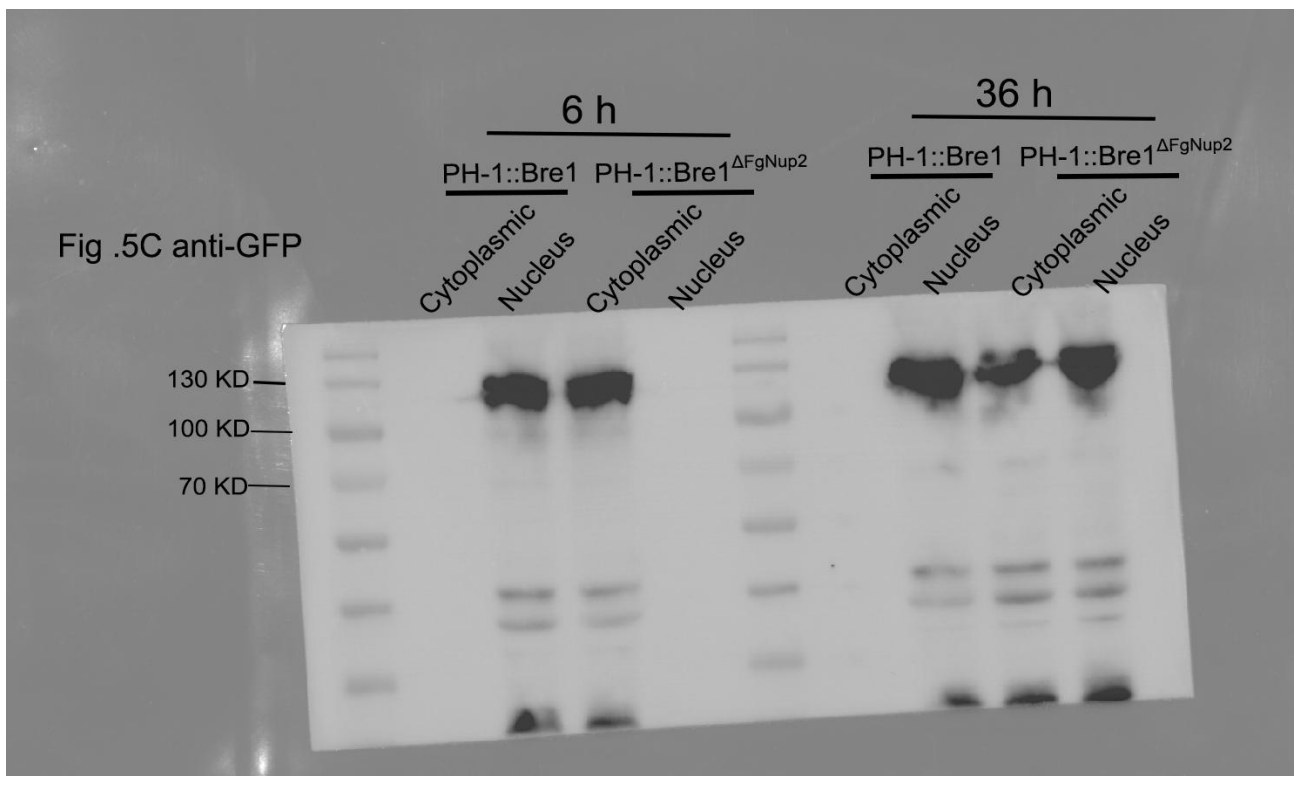
6 h
36 h
PH-1::Bre1
PH-1::Bre1ΔFgNup2
PH-1::Bre1
PH-1::Bre1ΔFgNup2
Fig .5C anti-GFP
Cytoplasmic
Nucleus
Cytoplasmic
Nucleus
Cytoplasmic
Nucleus
Cytoplasmic
Nucleus
130 KD
100 KD
70 KD

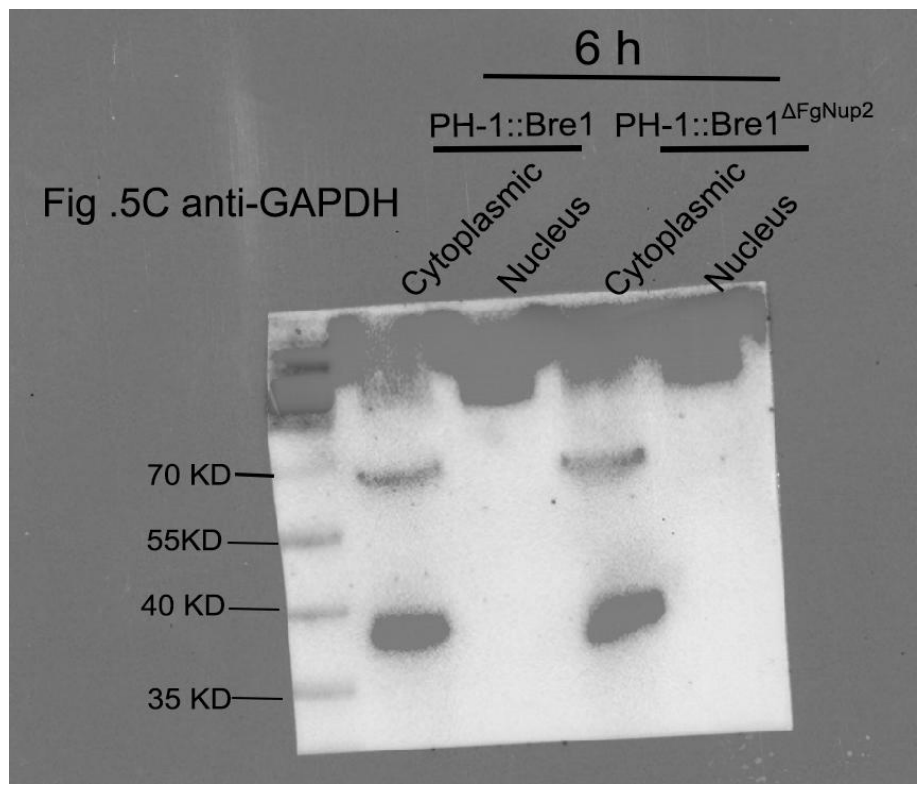
6 h
PH-1::Bre1
PH-1::Bre1ΔFgNup2
Fig .5C anti-GAPDH
Cytoplasmic
Nucleus
Cytoplasmic
Nucleus
70 KD
55KD
40 KD
35 KD

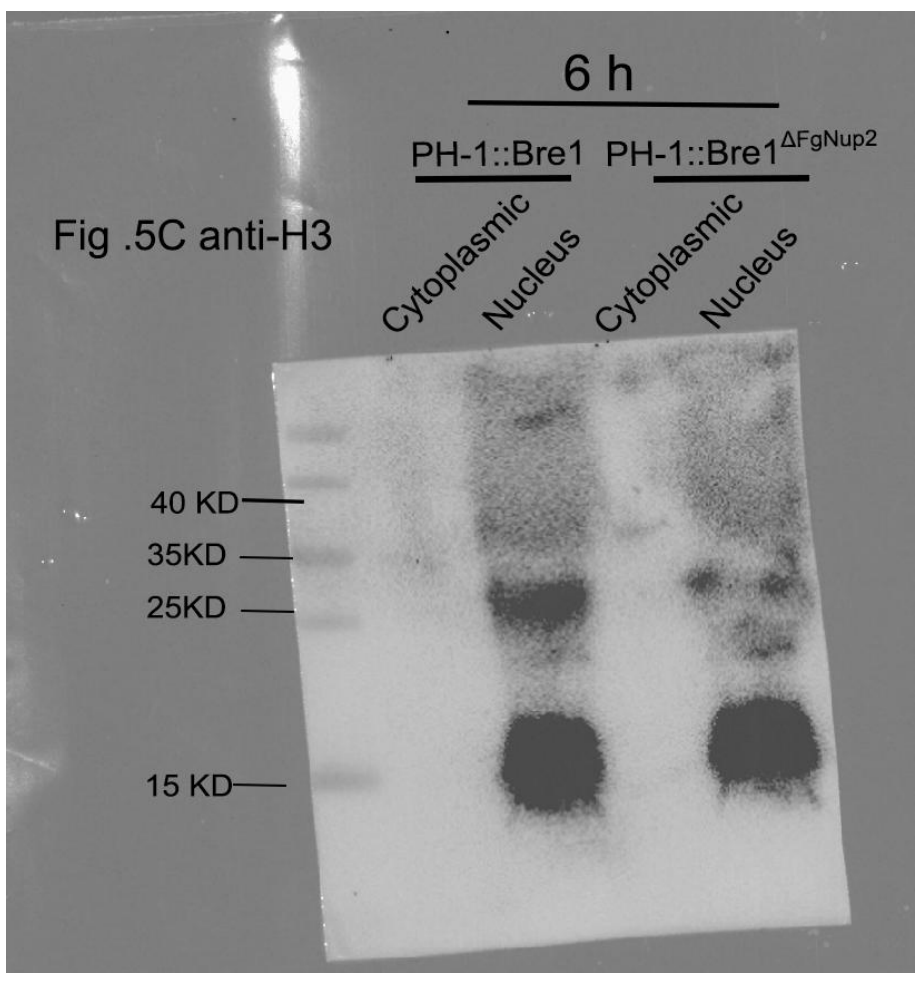
6 h
PH-1::Bre1
PH-1::Bre1ΔFgNup2
Fig .5C anti-H3
Cytoplasmic
Nucleus
Cytoplasmic
Nucleus
40 KD
35KD
25KD
15 KD

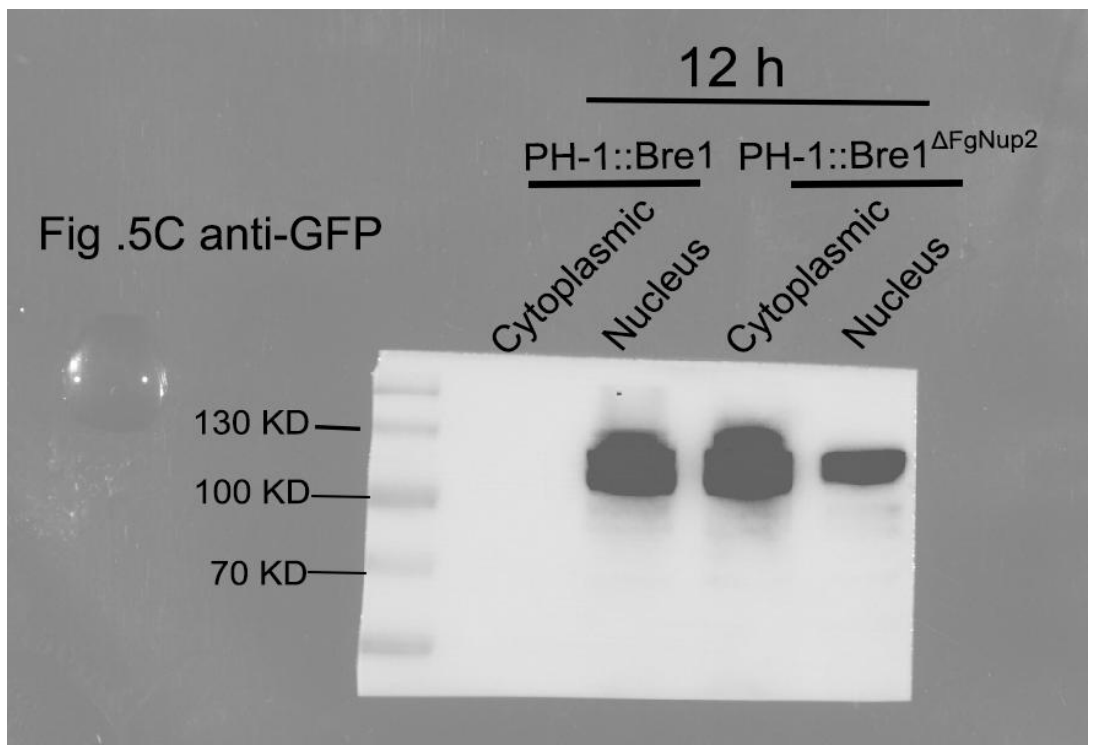
12 h
PH-1::Bre1
PH-1::Bre1ΔFgNup2
Fig .5C anti-GFP
Cytoplasmic
Nucleus
Cytoplasmic
Nucleus
130 KD
100 KD
70 KD

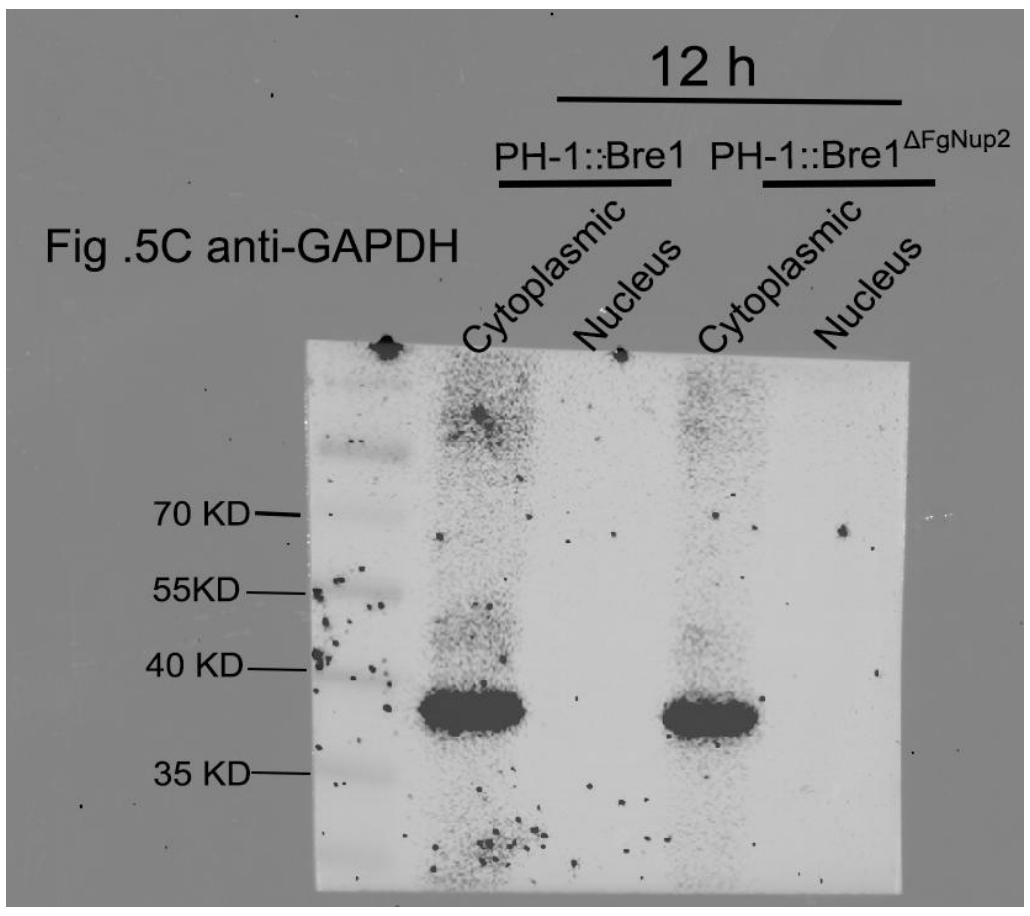

12 h
PH-1::Bre1
PH-1::Bre1$^{\Delta FgNup2}$
Fig .5C anti-GAPDH
Cytoplasmic
Nucleus
Cytoplasmic
Nucleus
70 KD
55KD
40 KD
35 KD

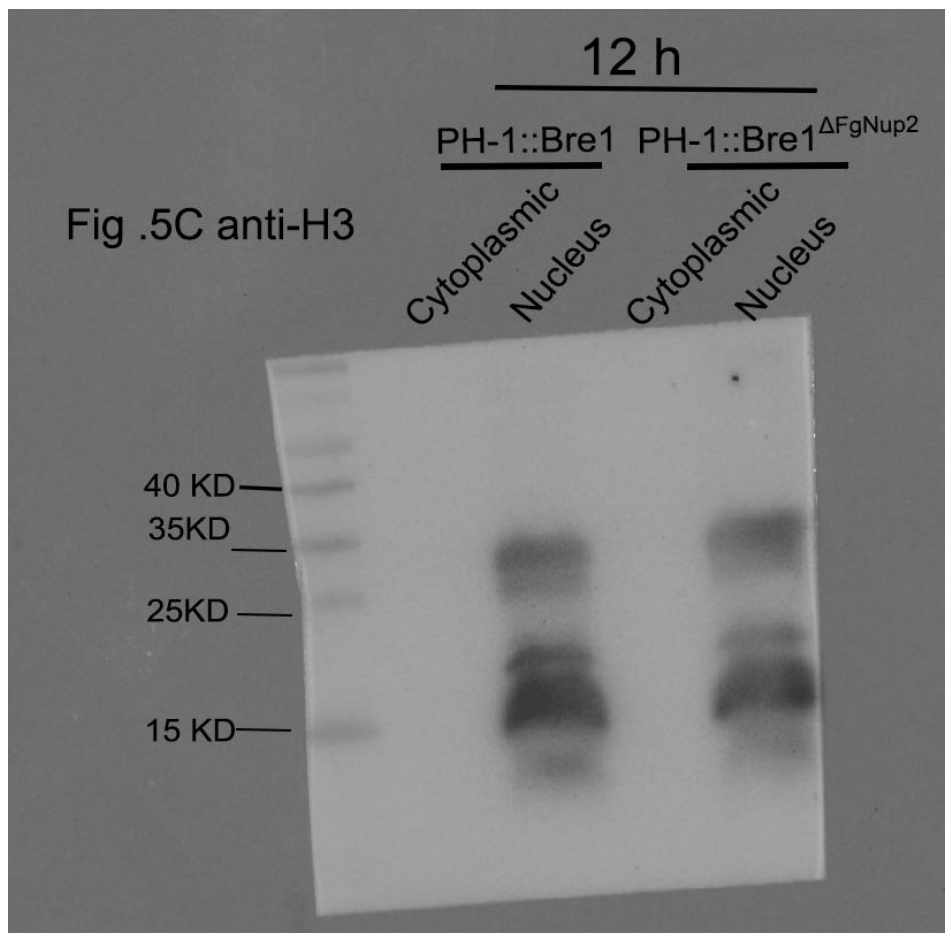

12 h
PH-1::Bre1
PH-1::Bre1$^{\Delta FgNup2}$
Fig .5C anti-H3
Cytoplasmic
Nucleus
Cytoplasmic
Nucleus
40 KD
35KD
25KD
15 KD

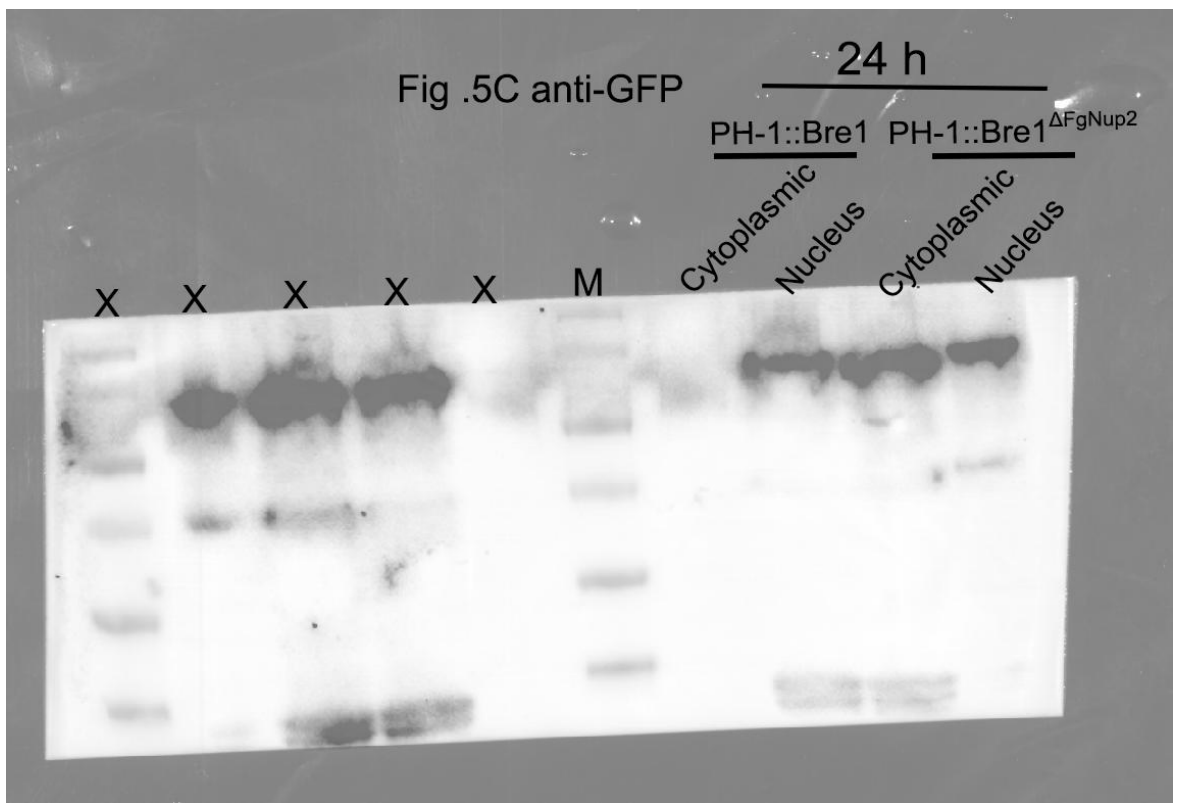

Fig .5C anti-GFP
24 h
PH-1::Bre1
PH-1::Bre1$^{\Delta FgNup2}$
X
X
X
X
X
M
Cytoplasmic
Nucleus
Cytoplasmic
Nucleus

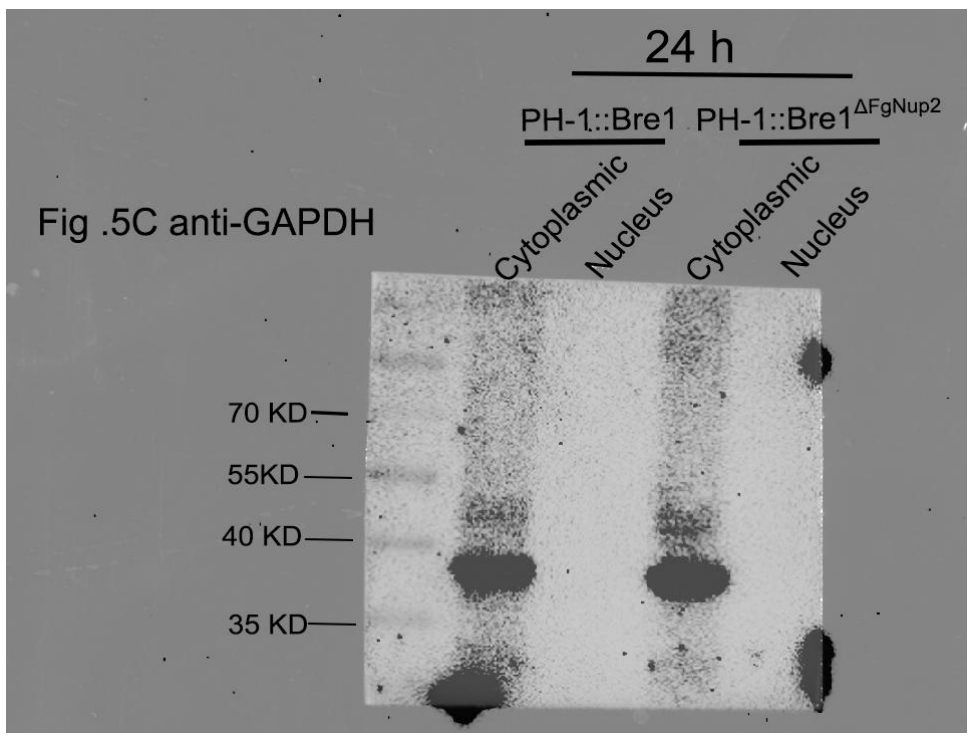

24 h
PH-1::Bre1
PH-1::Bre1$^{\Delta FgNup2}$
Fig .5C anti-GAPDH
Cytoplasmic
Nucleus
Cytoplasmic
Nucleus
70 KD
55KD
40 KD
35 KD

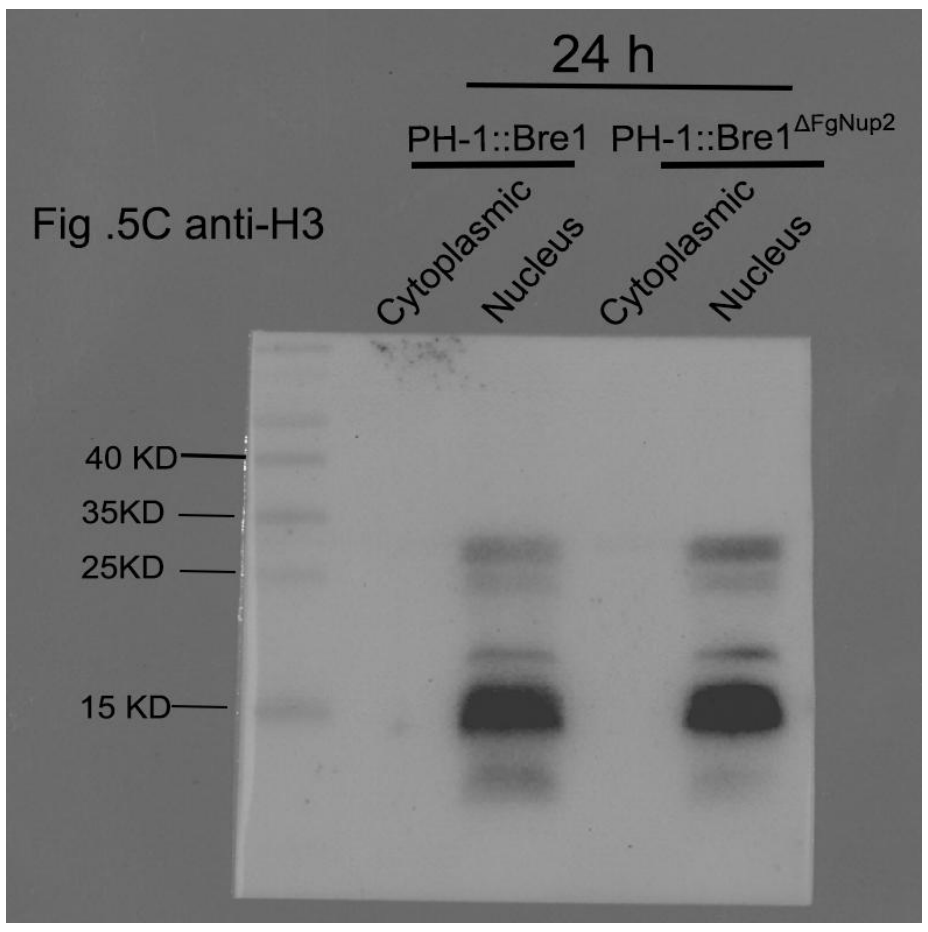

24 h
PH-1::Bre1
PH-1::Bre1$^{\Delta FgNup2}$
Fig .5C anti-H3
Cytoplasmic
Nucleus
Cytoplasmic
Nucleus
40 KD
35KD
25KD
15 KD

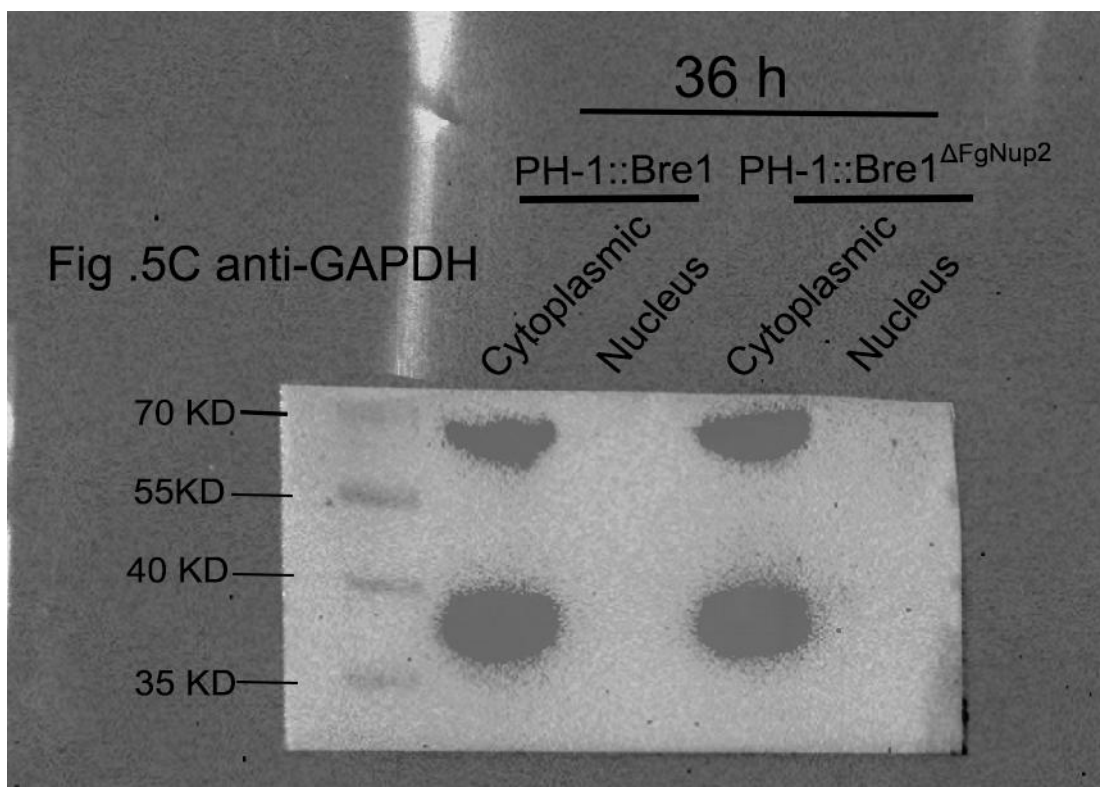

36 h
PH-1::Bre1
PH-1::Bre1$^{\Delta FgNup2}$
Fig .5C anti-GAPDH
Cytoplasmic
Nucleus
Cytoplasmic
Nucleus
70 KD
55KD
40 KD
35 KD

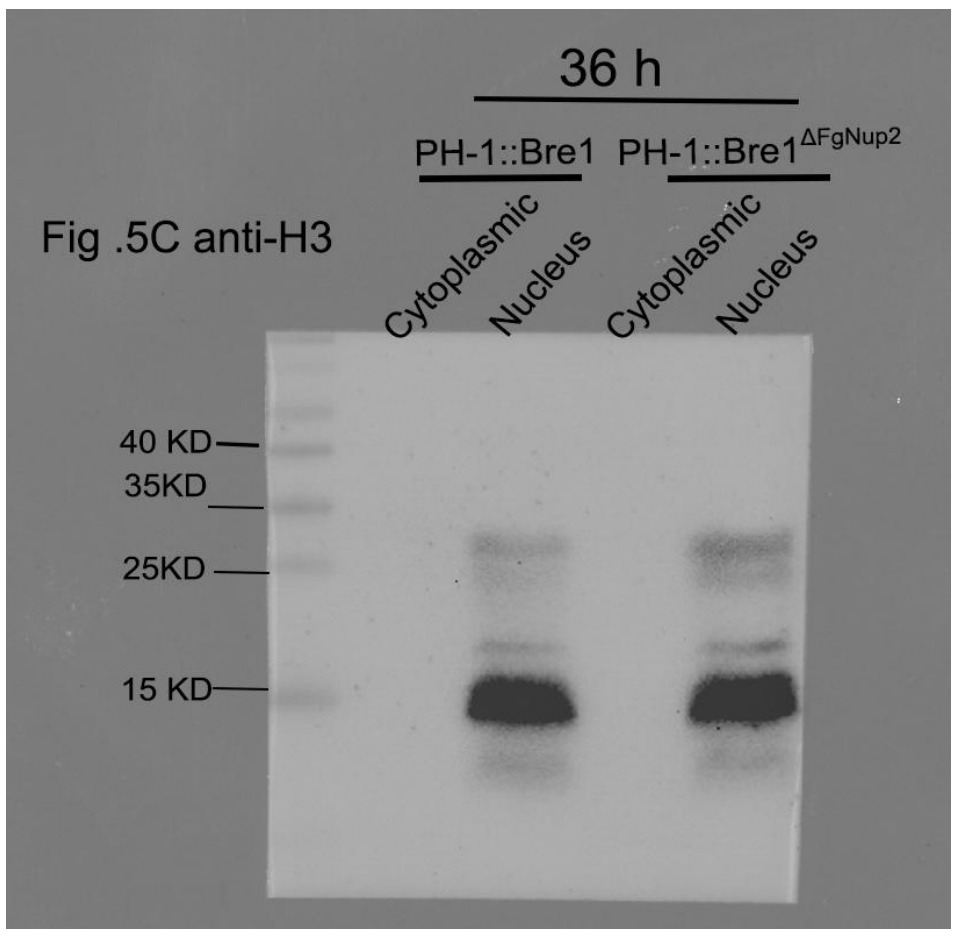
36 h
PH-1::Bre1
PH-1::Bre1ΔFgNup2
Fig .5C anti-H3
Cytoplasmic
Nucleus
Cytoplasmic
Nucleus
40 KD
35KD
25KD
15 KD

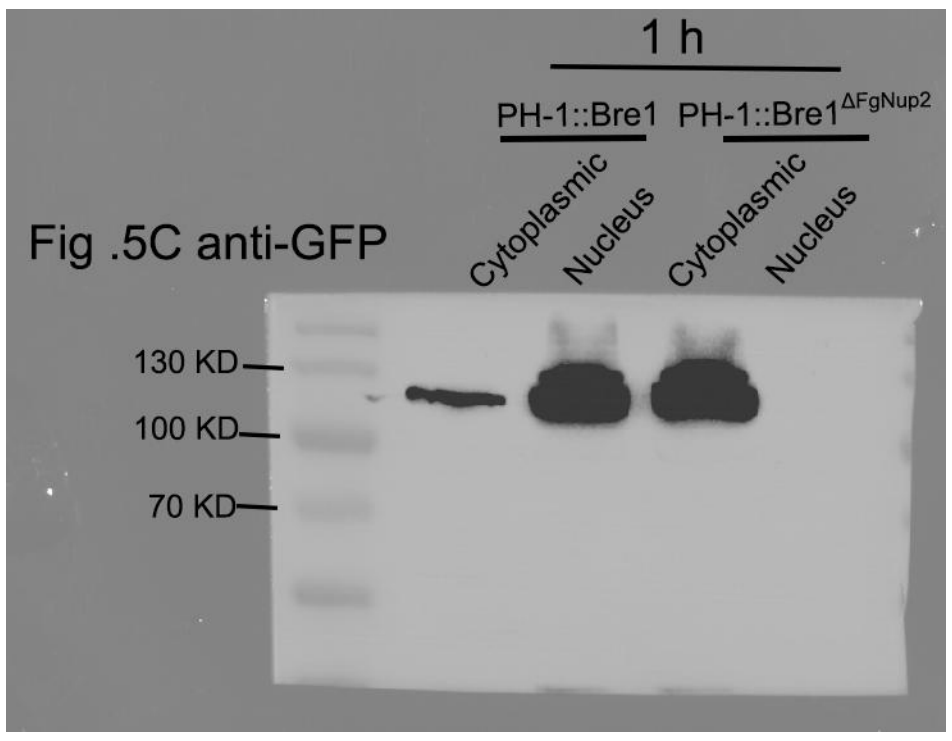
1 h
PH-1::Bre1
PH-1::Bre1ΔFgNup2
Fig .5C anti-GFP
Cytoplasmic
Nucleus
Cytoplasmic
Nucleus
130 KD
100 KD
70 KD

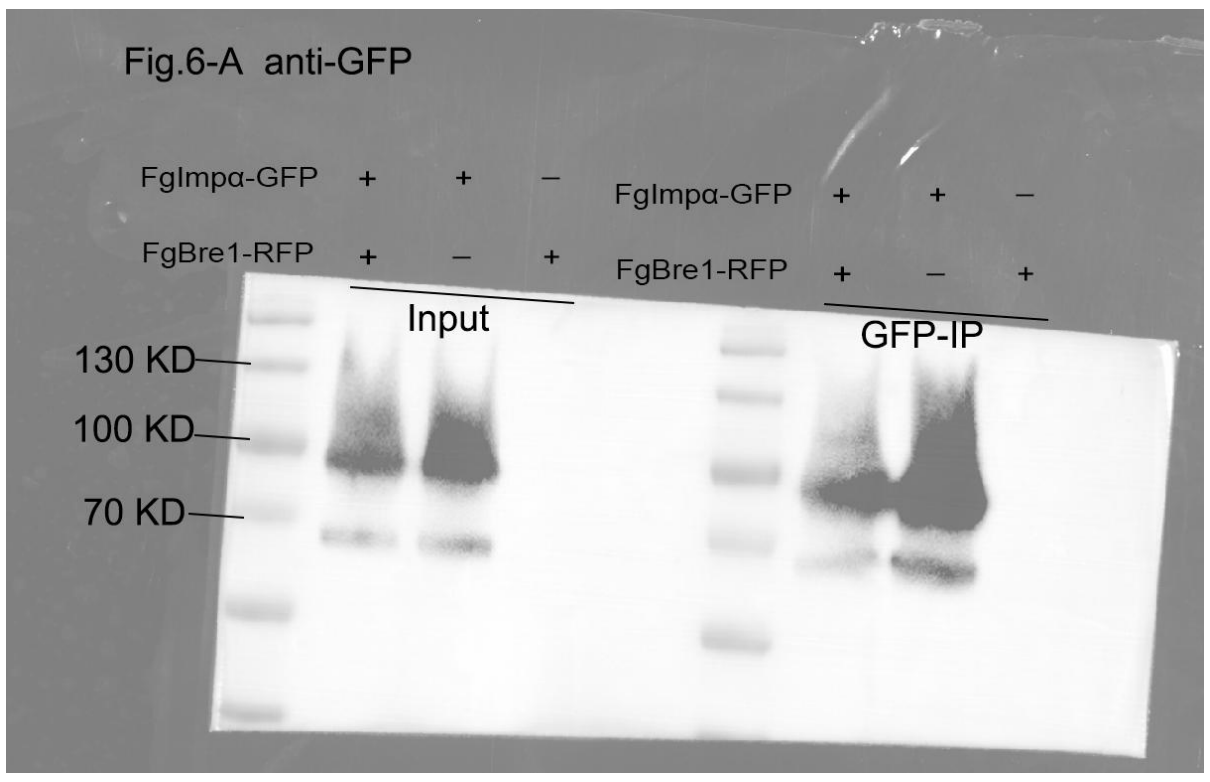
Fig.6-A anti-GFP
FgImpα-GFP + + –
FgBre1-RFP + – +
FgImpα-GFP + + –
FgBre1-RFP + – +
Input
GFP-IP
130 KD
100 KD
70 KD

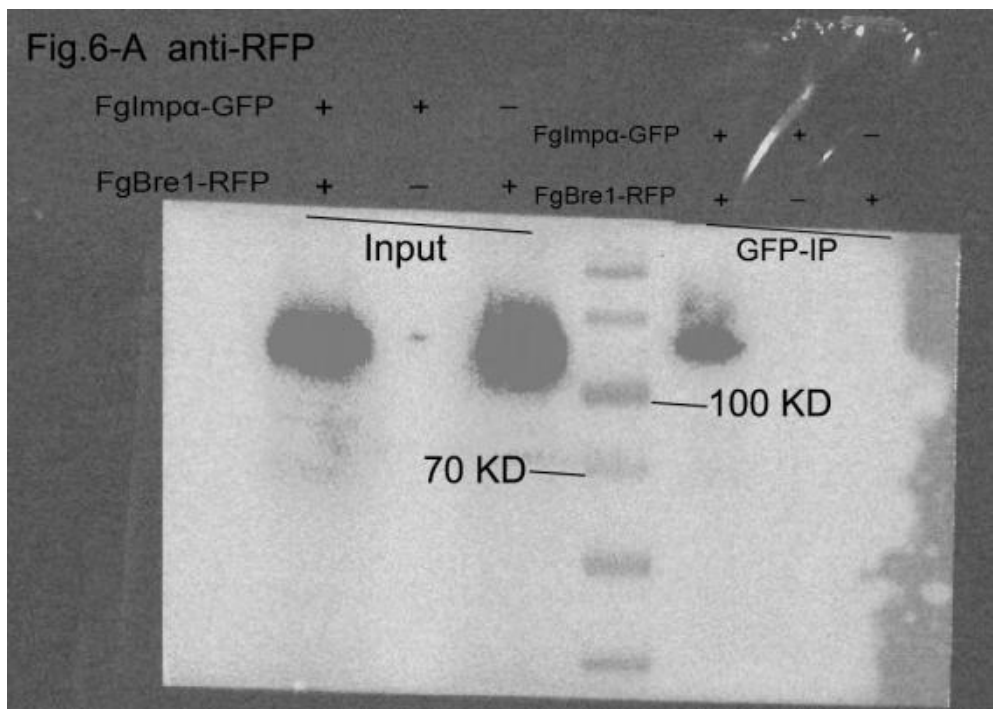
Fig.6-A anti-RFP
FgImpα-GFP + + –
FgBre1-RFP + – +
FgImpα-GFP + + –
FgBre1-RFP + – +
Input
GFP-IP
100 KD
70 KD

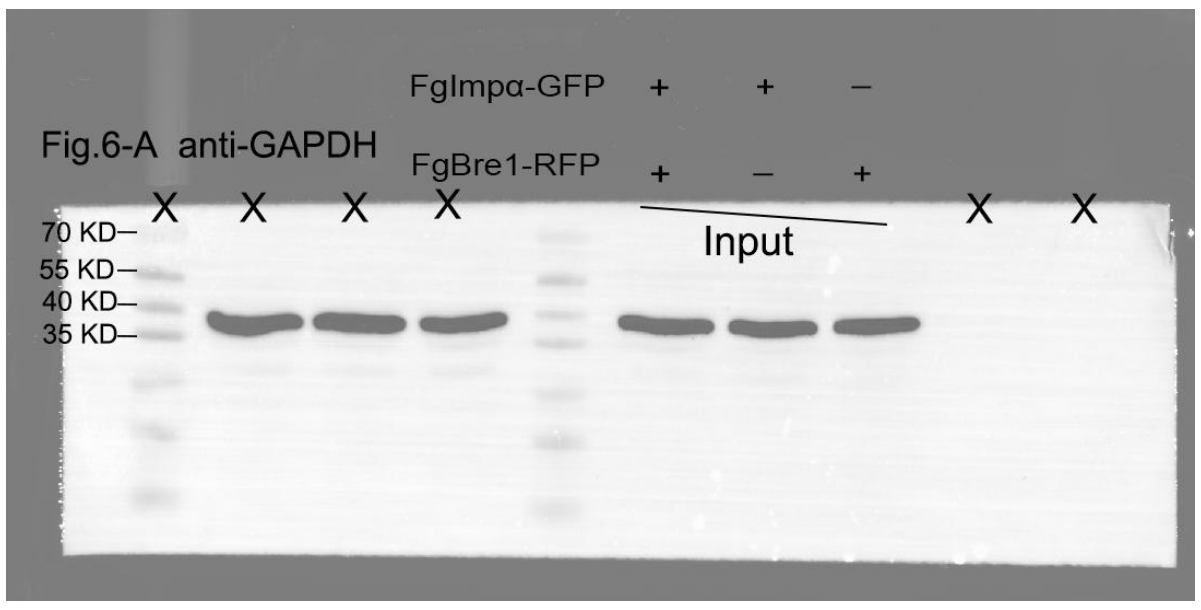
FgImpα-GFP + + –
Fig.6-A anti-GAPDH
FgBre1-RFP + – +
X X X X X X
70 KD
55 KD
40 KD
35 KD
Input

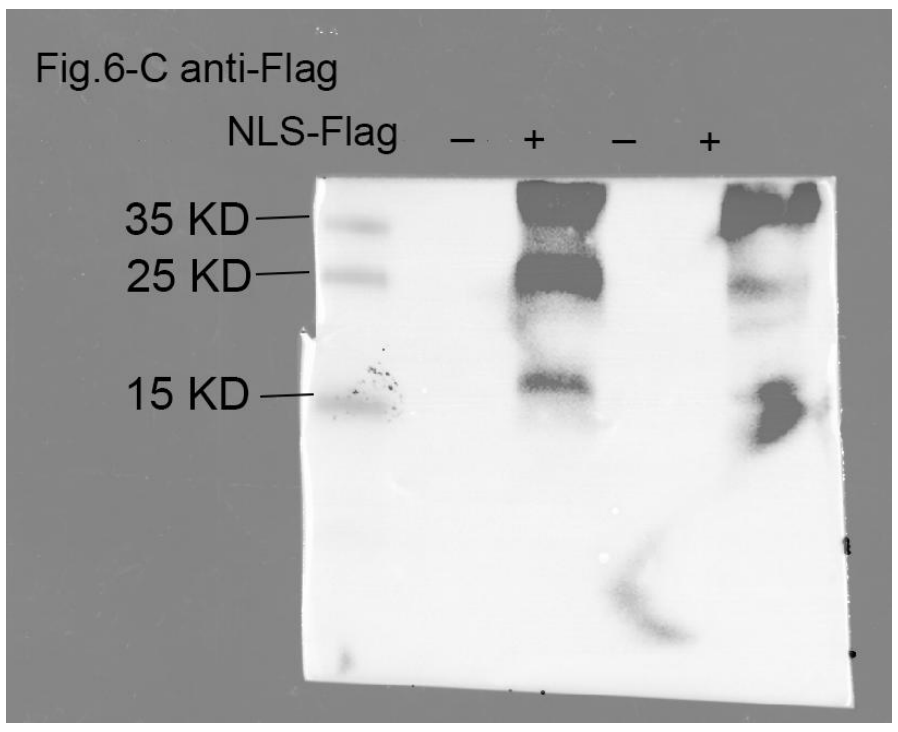
Fig.6-C anti-Flag
NLS-Flag – + – +
35 KD
25 KD
15 KD

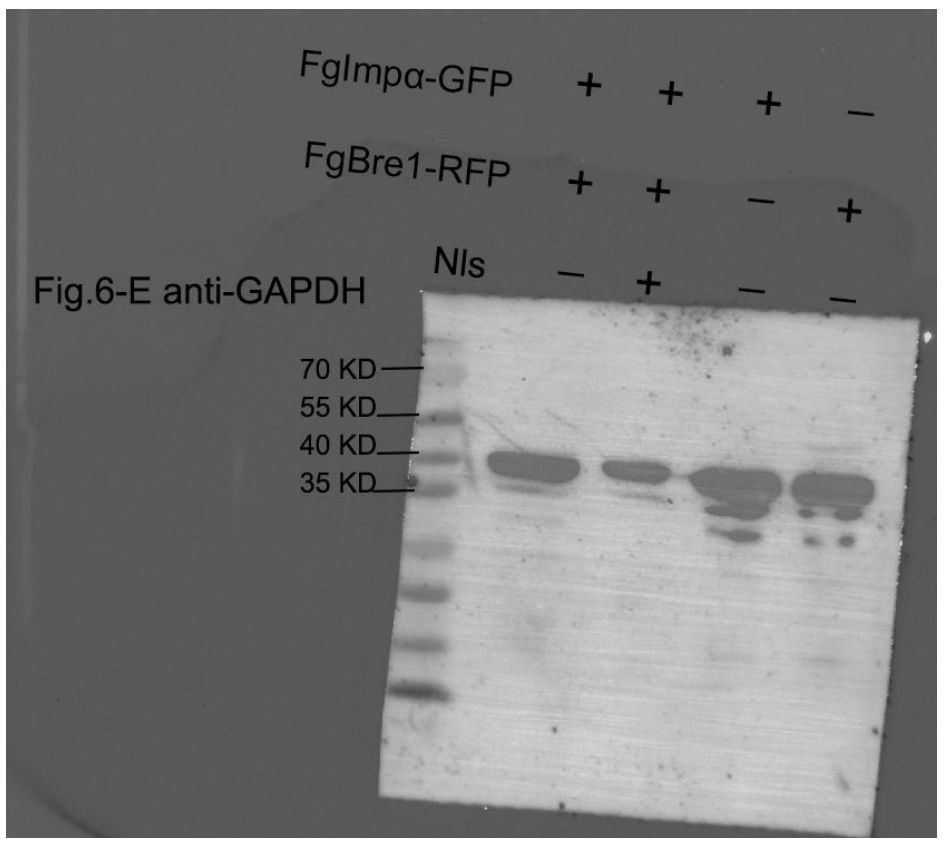
FgImpα-GFP + + + –
FgBre1-RFP + + – +
Fig.6-E anti-GAPDH
Nls – + – –
70 KD
55 KD
40 KD
35 KD

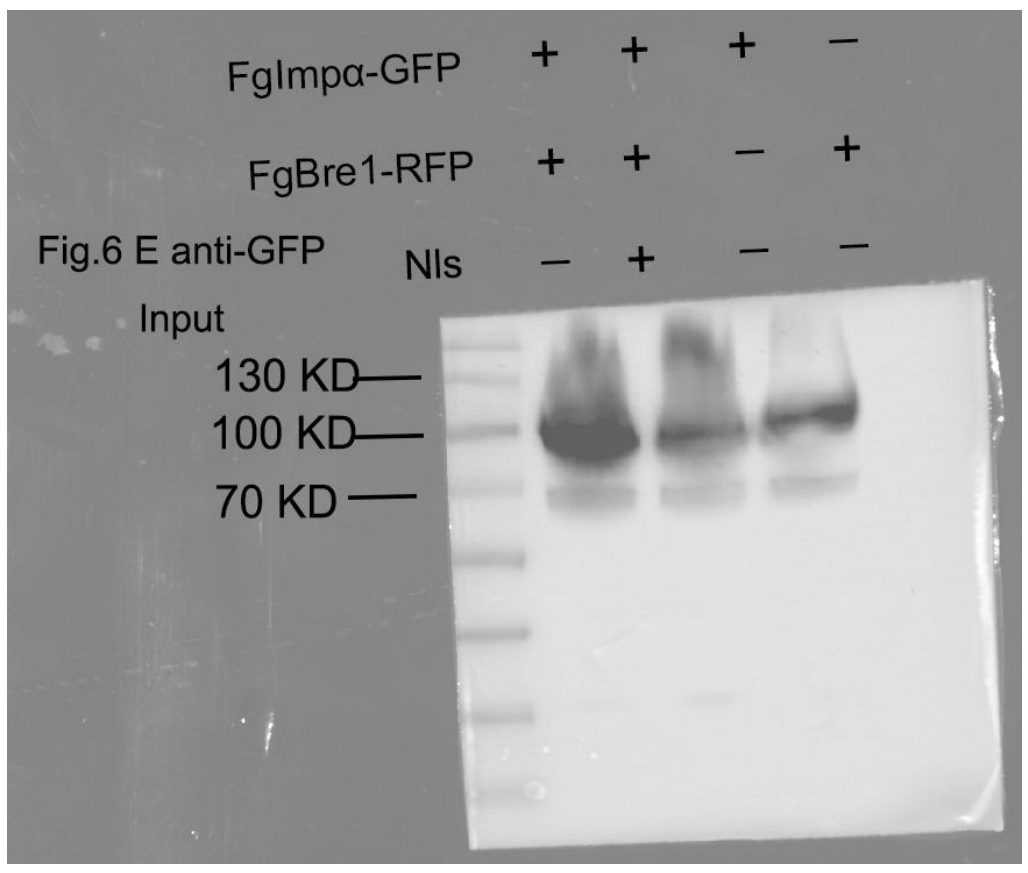
FgImpα-GFP + + + –
FgBre1-RFP + + – +
Fig.6 E anti-GFP
Nls – + – –
Input
130 KD
100 KD
70 KD

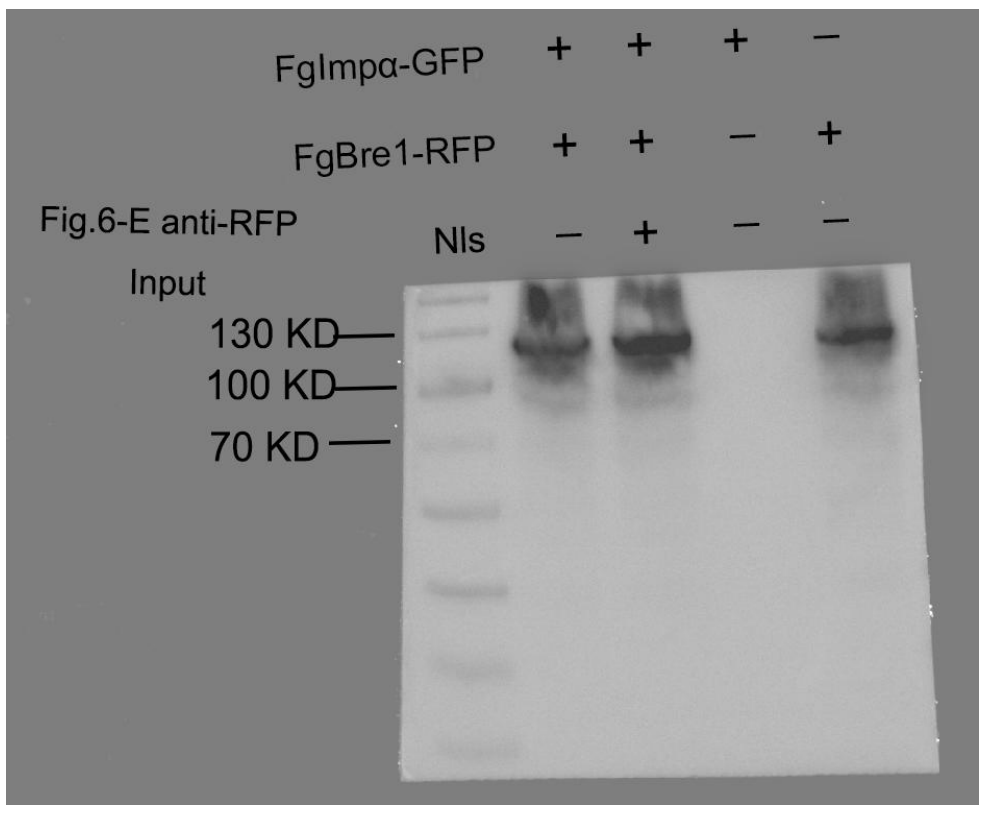
FgImpα-GFP + + + –
FgBre1-RFP + + – +
Fig.6-E anti-RFP
Input
Nls – + – –
130 KD
100 KD
70 KD

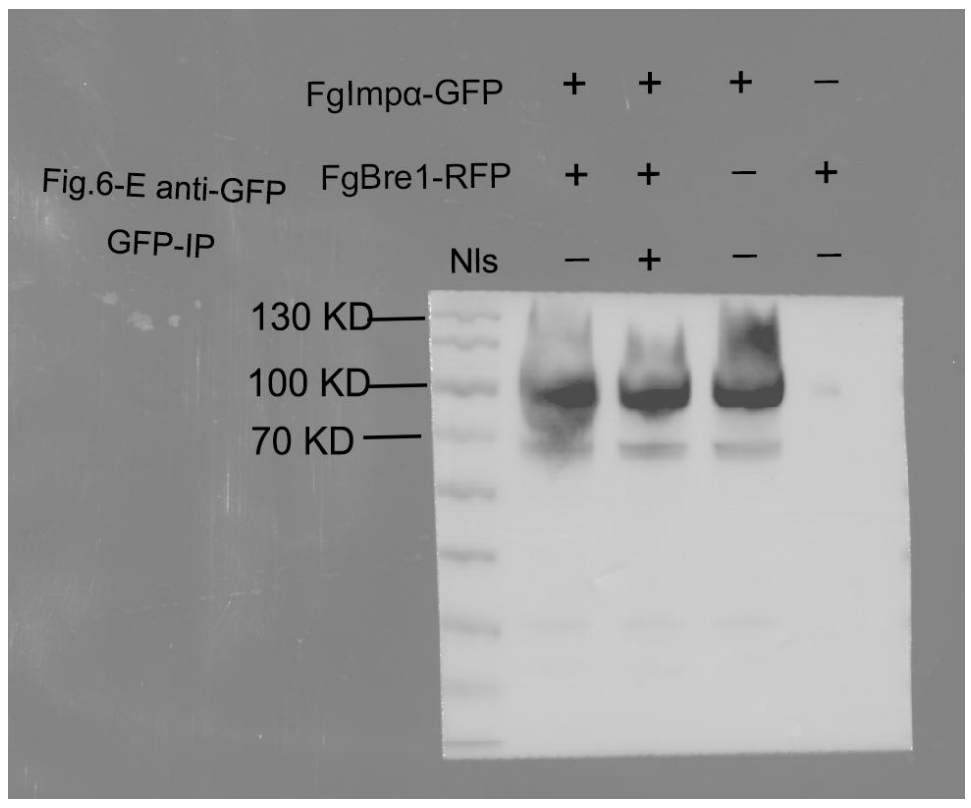
FgImpα-GFP + + + –
Fig.6-E anti-GFP
GFP-IP
FgBre1-RFP + + – +
Nls – + – –
130 KD
100 KD
70 KD

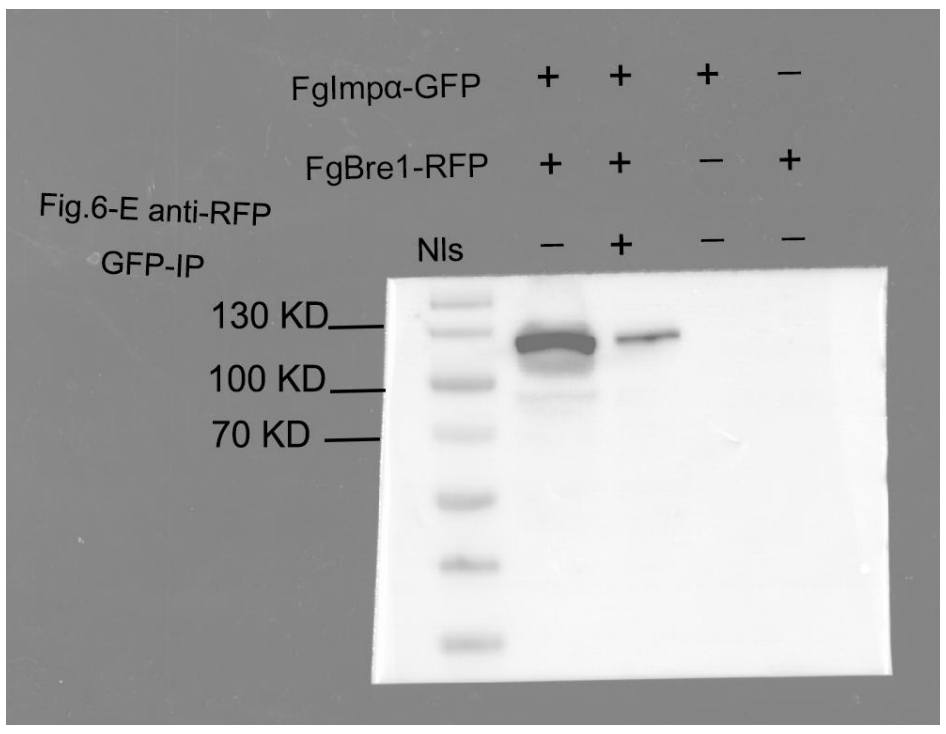
FgImpα-GFP + + + –
FgBre1-RFP + + – +
Fig.6-E anti-RFP
GFP-IP
Nls – + – –
130 KD
100 KD
70 KD

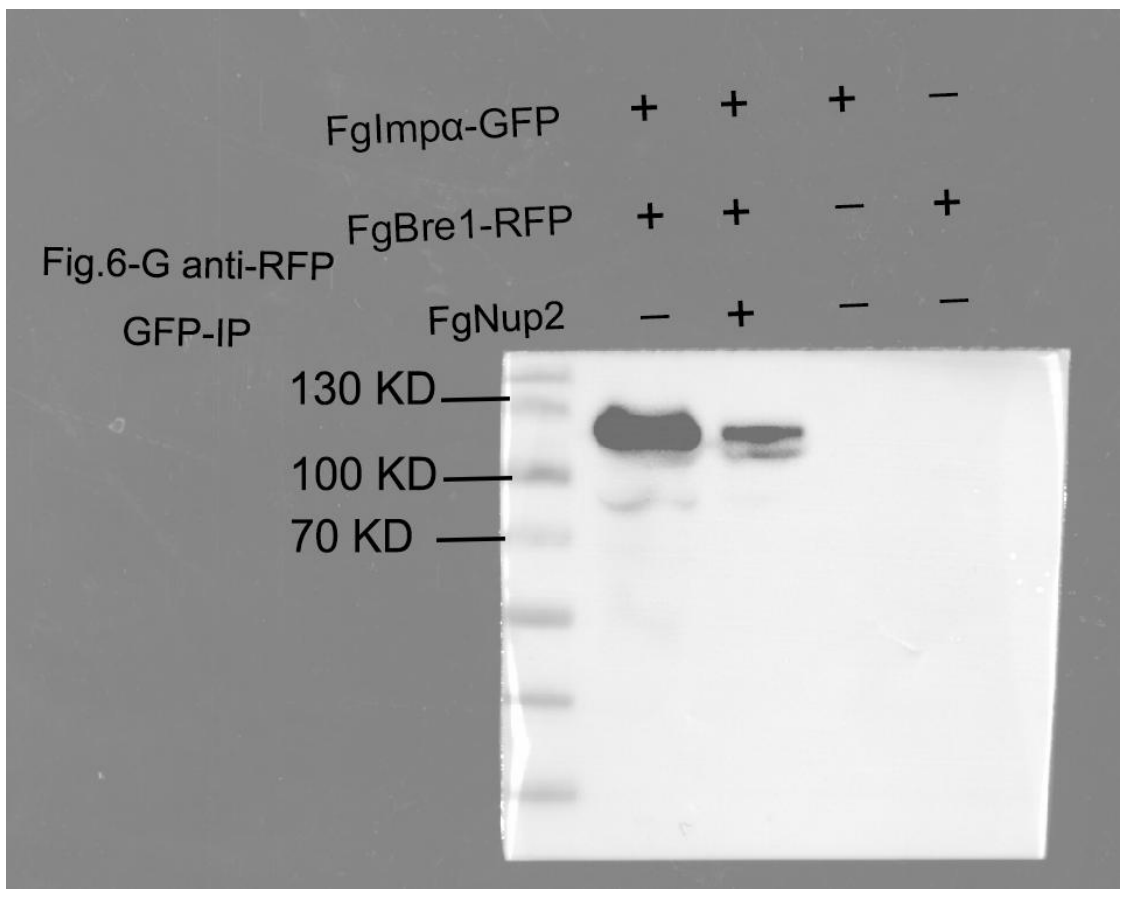
FgImpα-GFP + + + –
Fig.6-G anti-RFP
GFP-IP
FgBre1-RFP + + – +
FgNup2 – + – –
130 KD
100 KD
70 KD

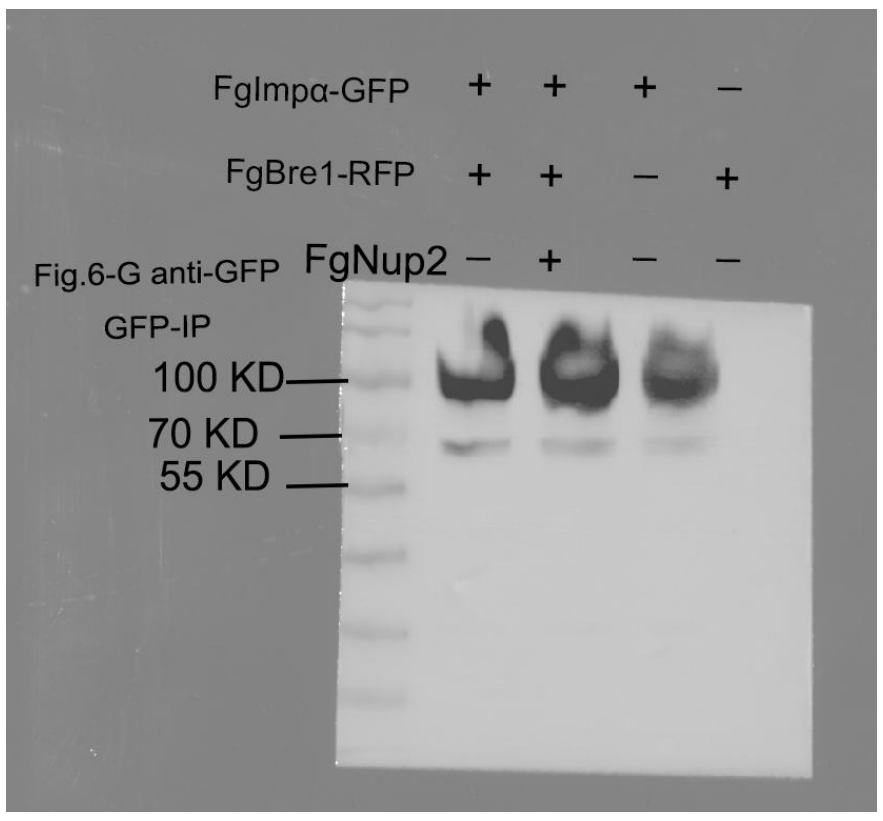
FgImpα-GFP + + + –
FgBre1-RFP + + – +
Fig.6-G anti-GFP
GFP-IP
FgNup2 – + – –
100 KD
70 KD
55 KD

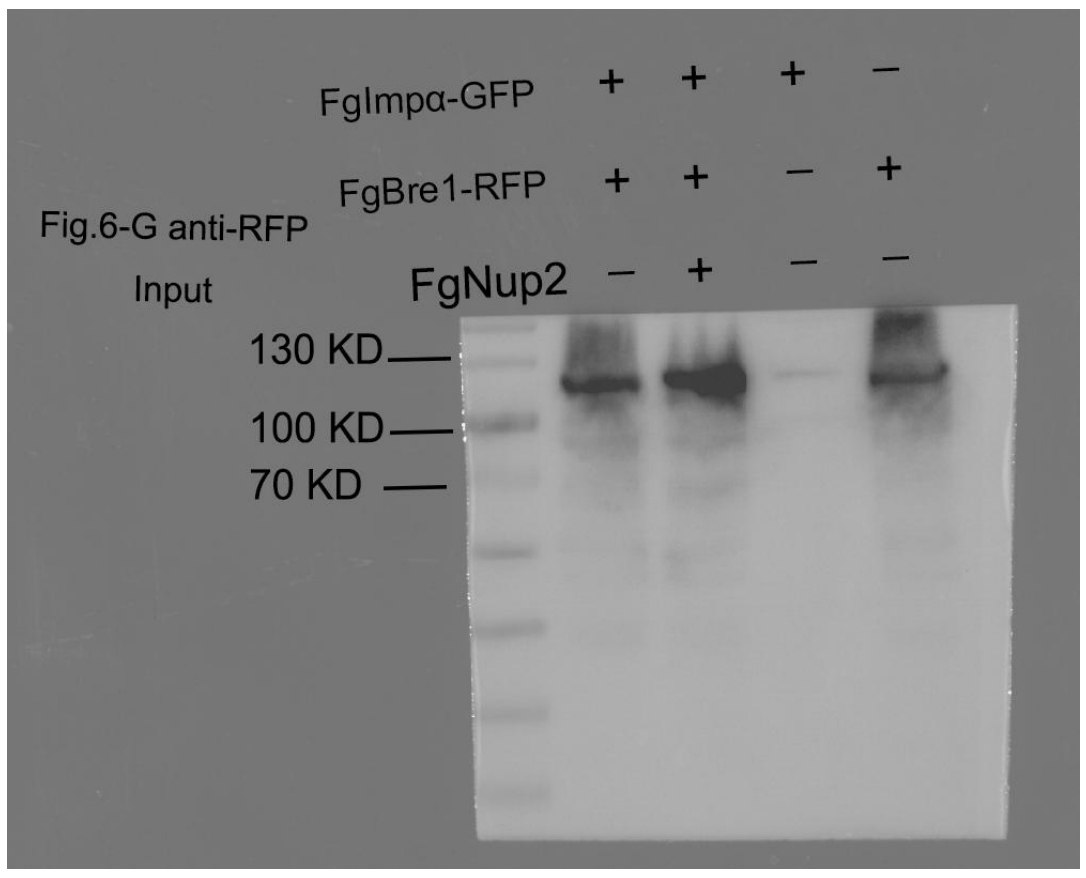
FgImpα-GFP + + + –
FgBre1-RFP + + – +
Fig.6-G anti-RFP
Input
FgNup2 – + – –
130 KD
100 KD
70 KD

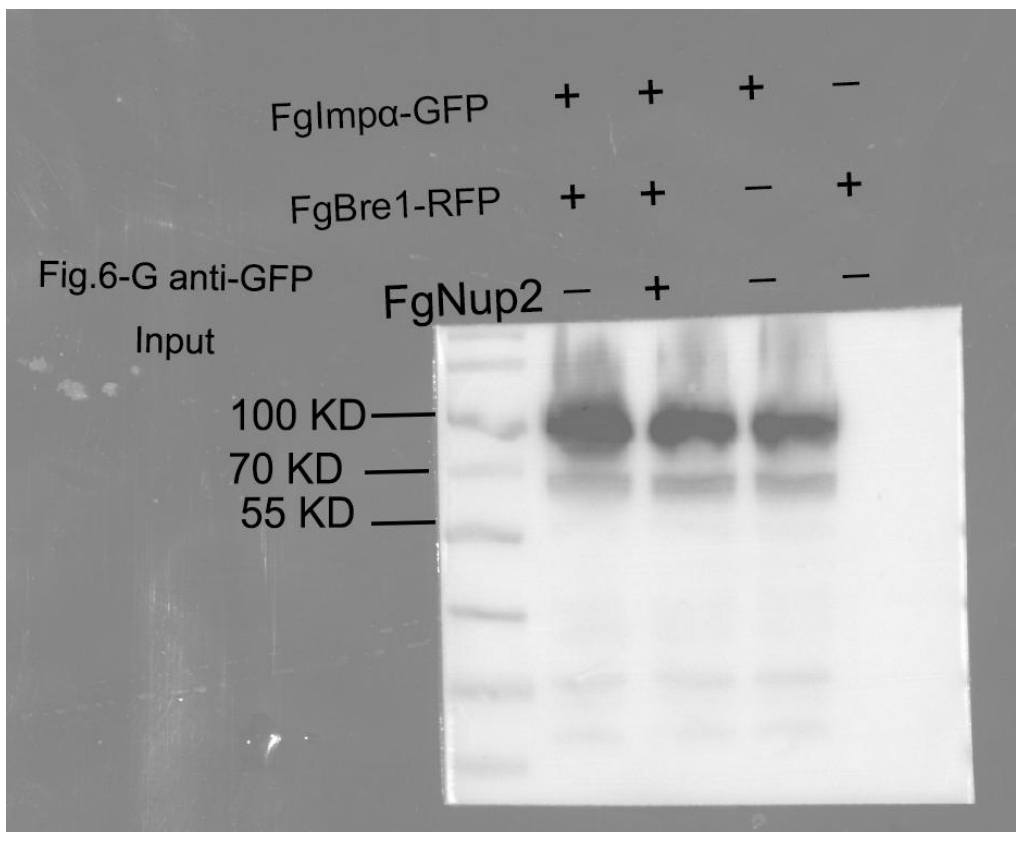
FgImpα-GFP + + + –
FgBre1-RFP + + – +
Fig.6-G anti-GFP
Input
FgNup2 – + – –
100 KD
70 KD
55 KD

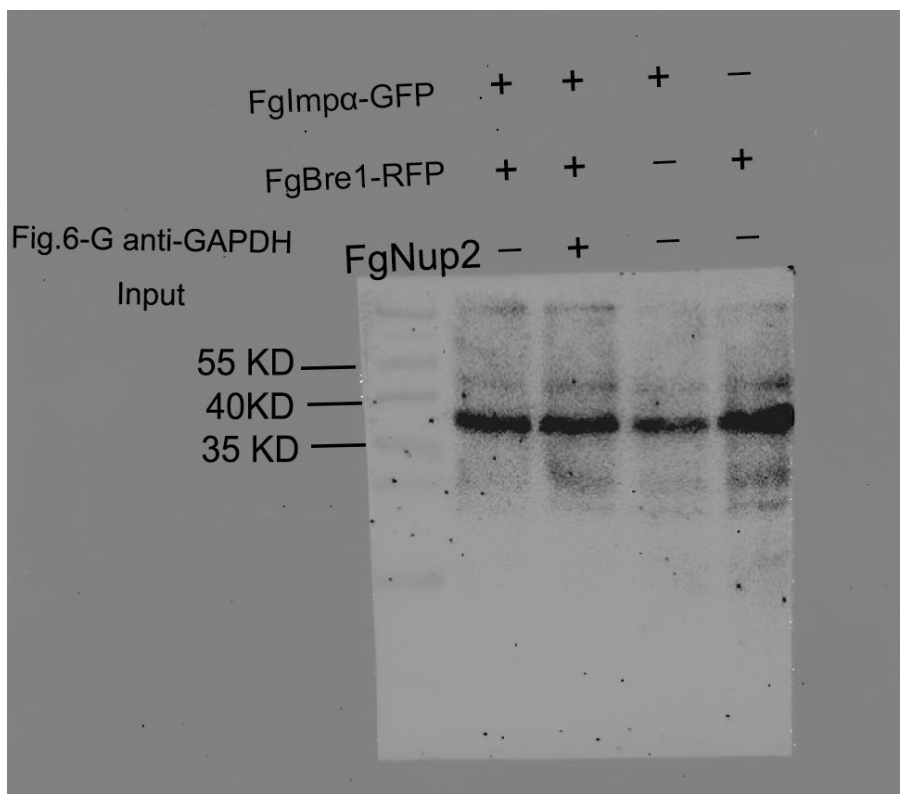
FgImpα-GFP + + + –
FgBre1-RFP + + – +
Fig.6-G anti-GAPDH
Input
FgNup2 – + – –
55 KD
40KD
35 KD

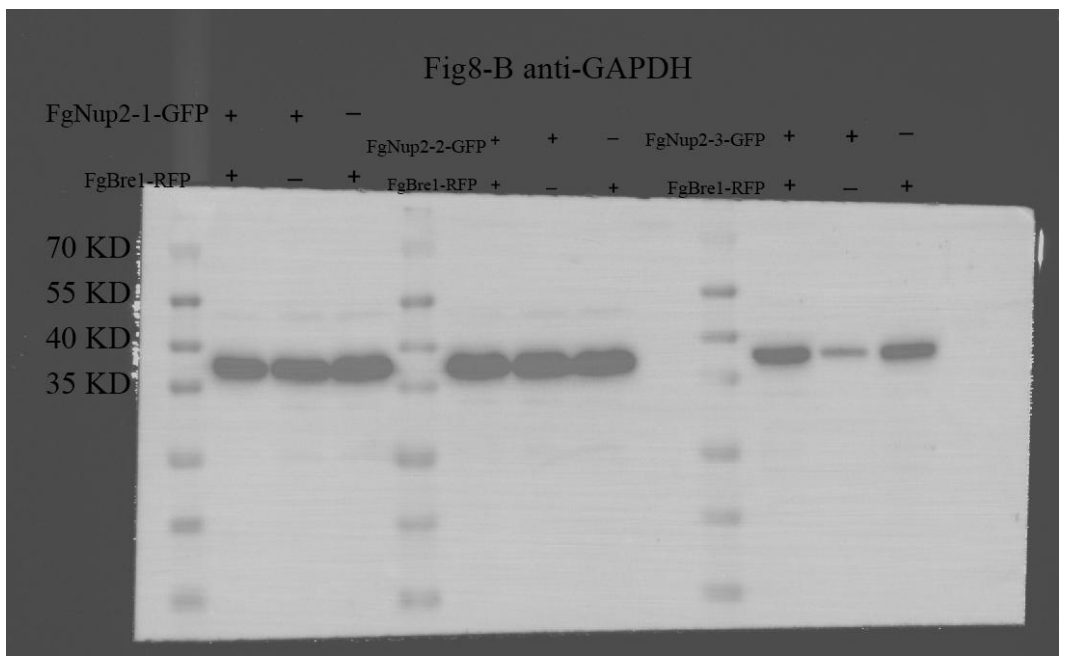
Fig8-B anti-GAPDH
FgNup2-1-GFP + + −
FgBre1-RFP + − +
FgNup2-2-GFP + + −
FgBre1-RFP + − +
FgNup2-3-GFP + + −
FgBre1-RFP + − +
70 KD
55 KD
40 KD
35 KD

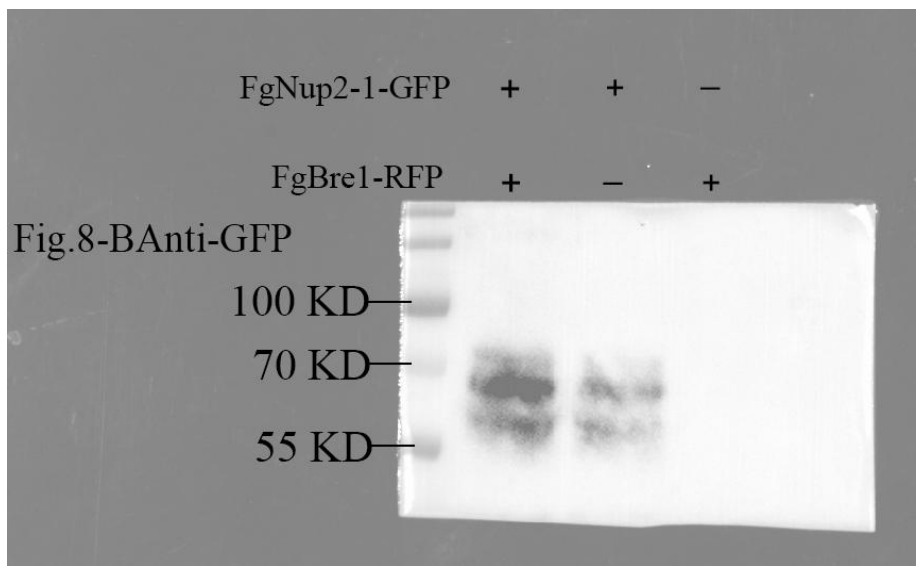
FgNup2-1-GFP + + −
FgBre1-RFP + − +
Fig.8-BAnti-GFP
100 KD
70 KD
55 KD

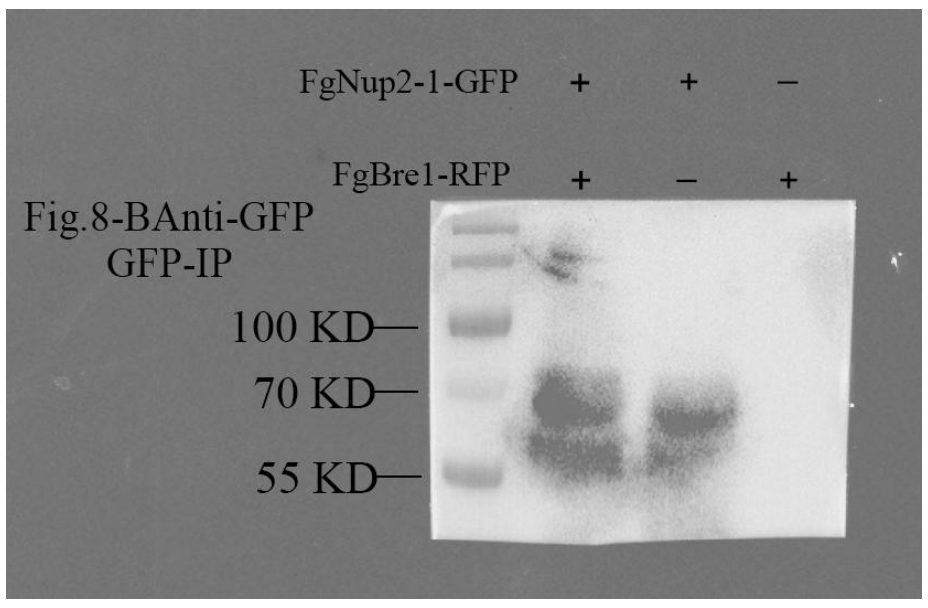
FgNup2-1-GFP + + −
FgBre1-RFP + − +
Fig.8-BAnti-GFP
GFP-IP
100 KD
70 KD
55 KD

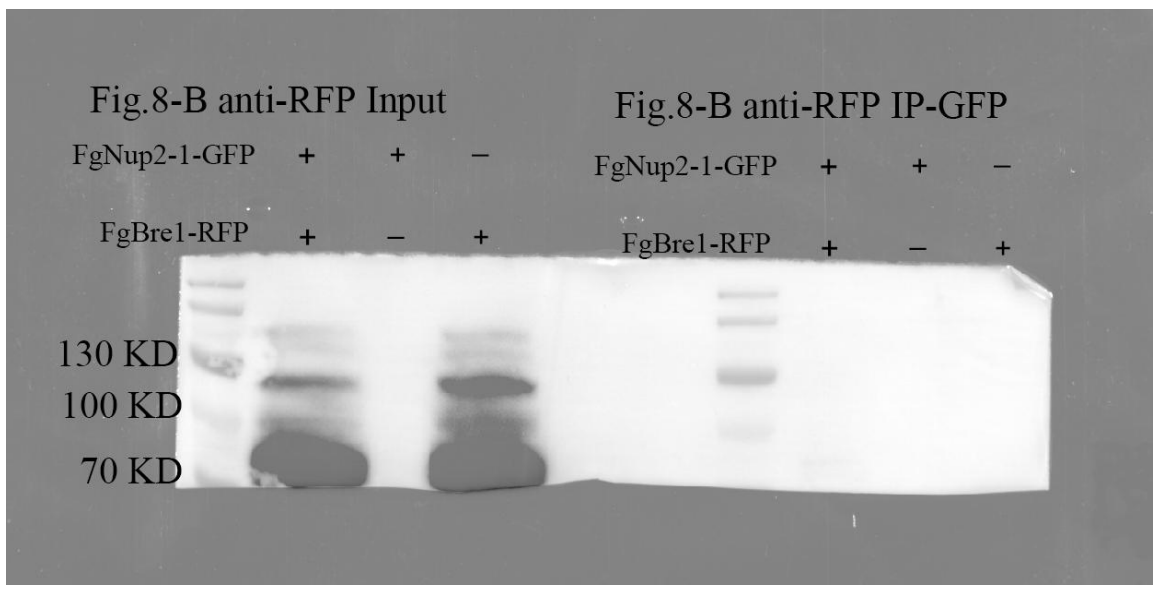
Fig.8-B anti-RFP Input
Fig.8-B anti-RFP IP-GFP
FgNup2-1-GFP + + −
FgBre1-RFP + − +
FgNup2-1-GFP + + −
FgBre1-RFP + − +
130 KD
100 KD
70 KD

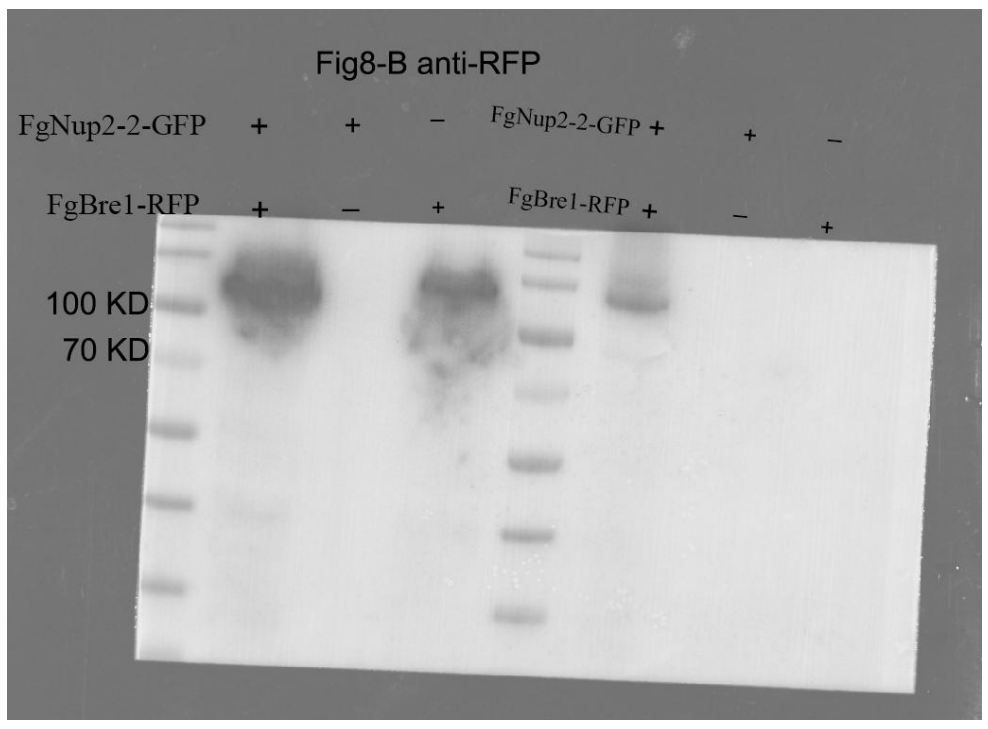
Fig8-B anti-RFP
FgNup2-2-GFP + + −
FgBre1-RFP + − +
FgNup2-2-GFP + + −
FgBre1-RFP + − +
100 KD
70 KD

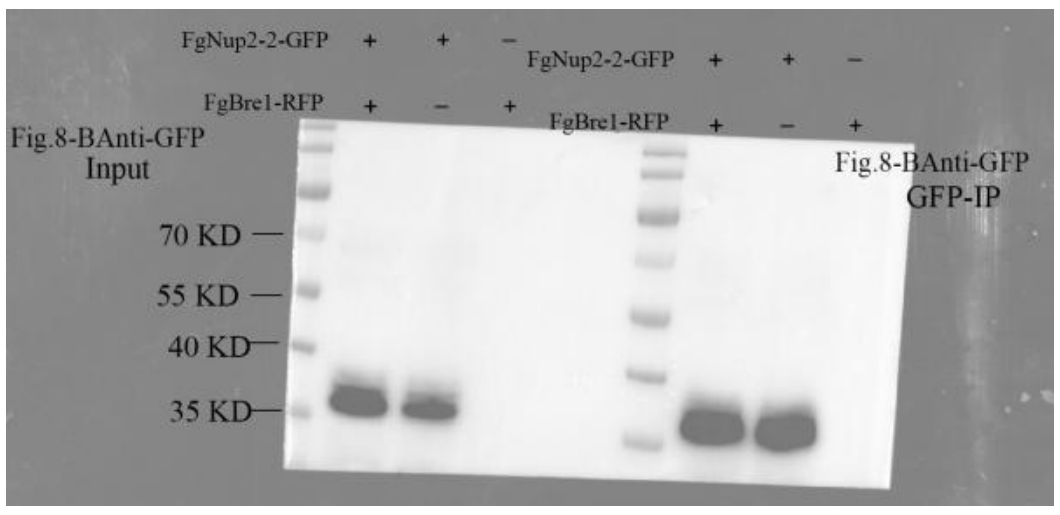
FgNup2-2-GFP + + −
FgBre1-RFP + − +
FgNup2-2-GFP + + −
FgBre1-RFP + − +
Fig.8-BAnti-GFP
Input
Fig.8-BAnti-GFP
GFP-IP
70 KD
55 KD
40 KD
35 KD

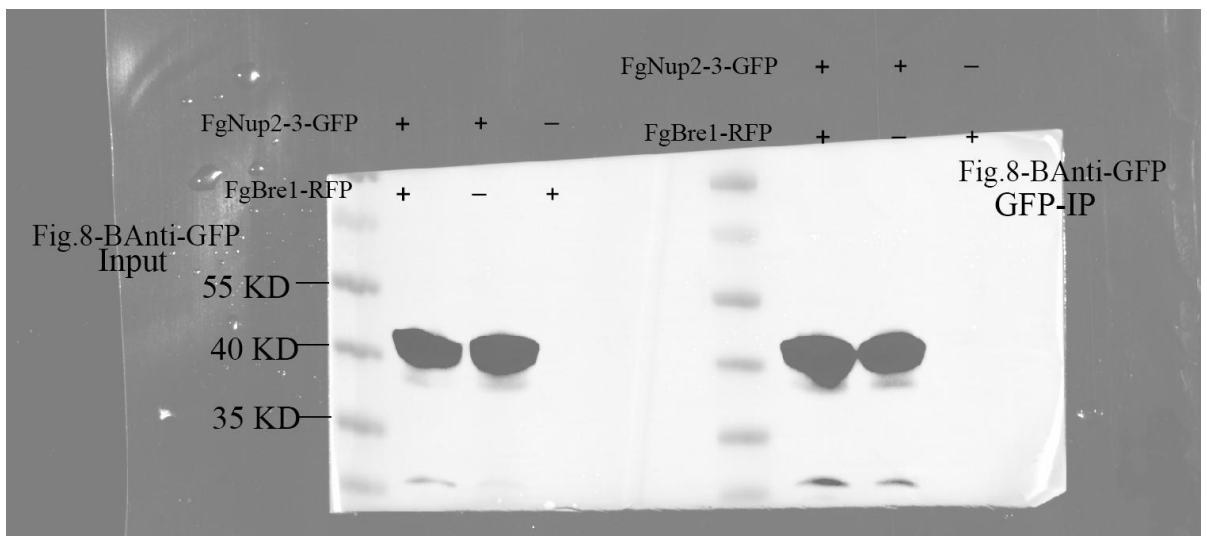
FgNup2-3-GFP + + −
FgBre1-RFP + − +
FgNup2-3-GFP + + −
FgBre1-RFP + − +
Fig.8-BAnti-GFP
Input
Fig.8-BAnti-GFP
GFP-IP
55 KD
40 KD
35 KD

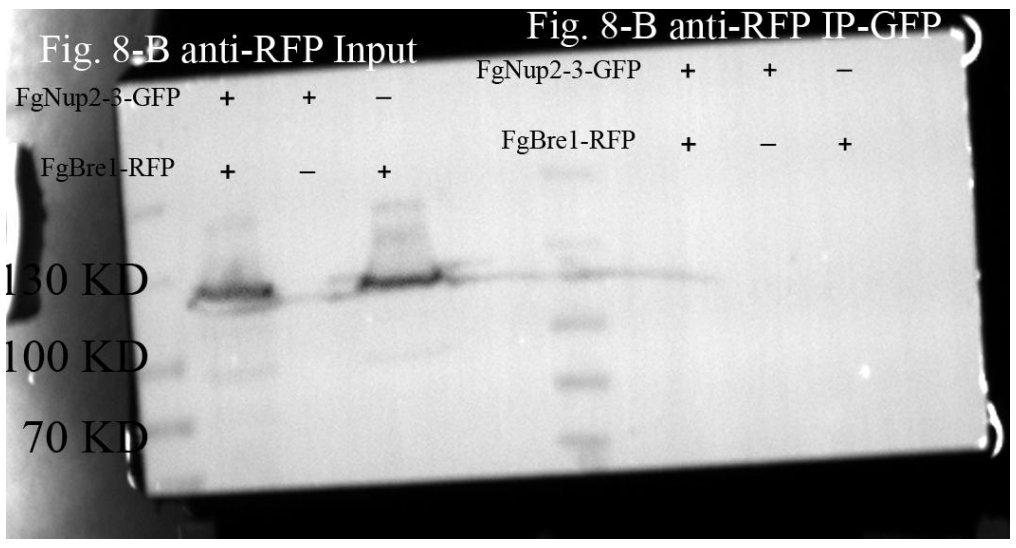
Fig. 8-B anti-RFP Input
Fig. 8-B anti-RFP IP-GFP
FgNup2-3-GFP + + −
FgBre1-RFP + − +
FgNup2-3-GFP + + −
FgBre1-RFP + − +
130 KD
100 KD
70 KD

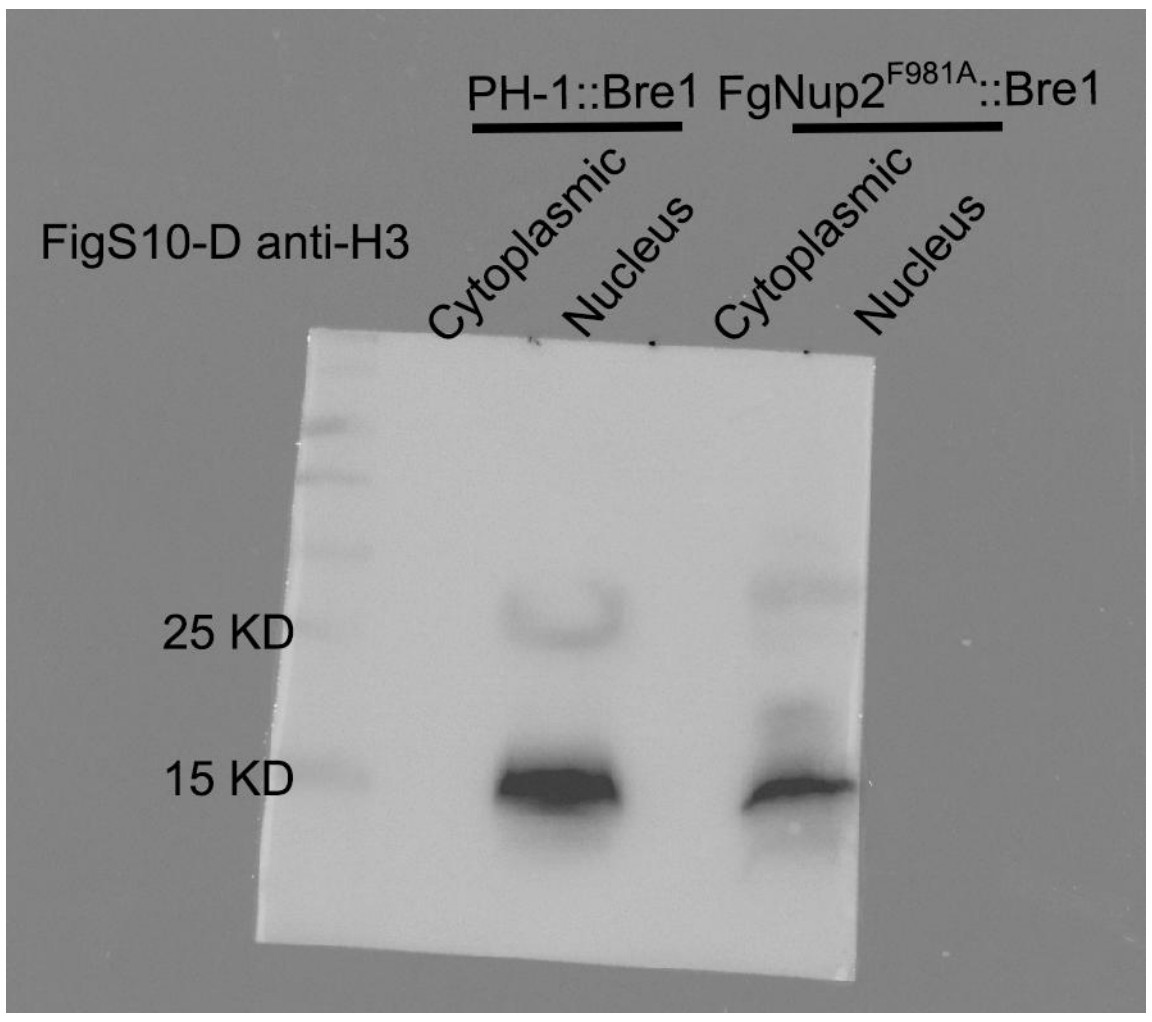
PH-1::Bre1 FgNup2F981A::Bre1
FigS10-D anti-H3
Cytoplasmic
Nucleus
Cytoplasmic
Nucleus
25 KD
15 KD

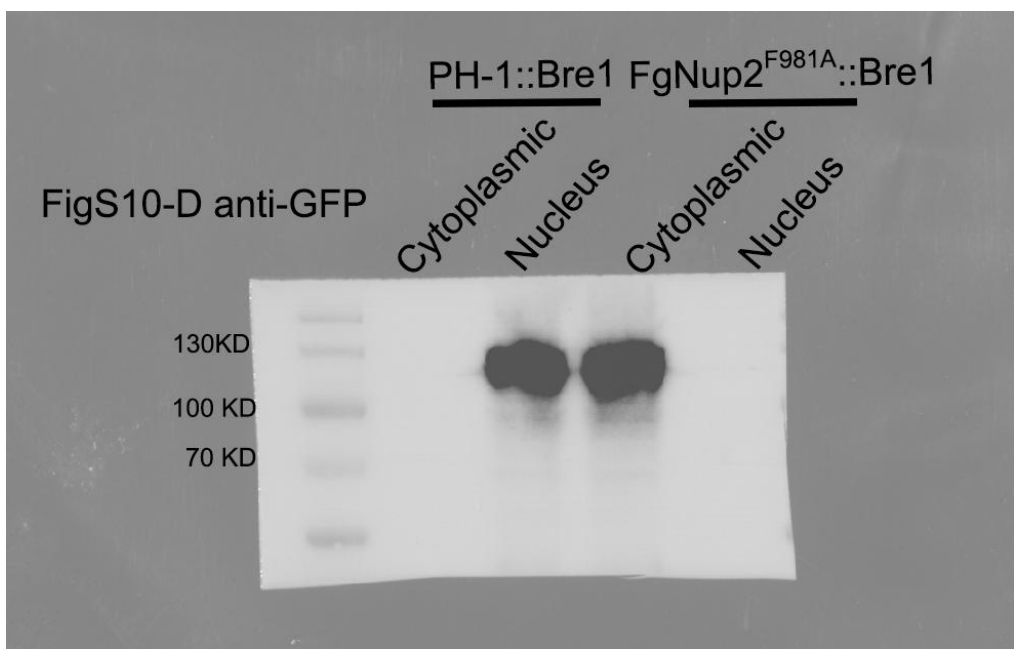
PH-1::Bre1 FgNup2F981A::Bre1
FigS10-D anti-GFP
Cytoplasmic
Nucleus
Cytoplasmic
Nucleus
130KD
100 KD
70 KD

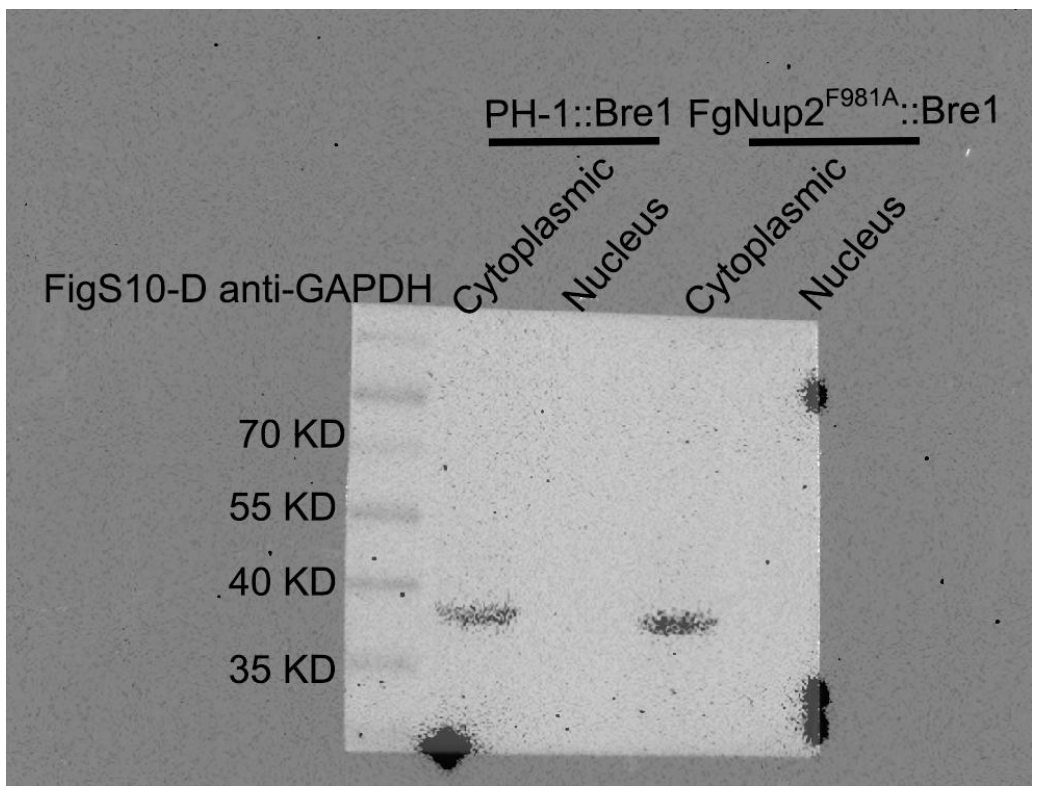
PH-1::Bre1
FgNup2F981A::Bre1
FigS10-D anti-GAPDH
Cytoplasmic
Nucleus
Cytoplasmic
Nucleus
70 KD
55 KD
40 KD
35 KD

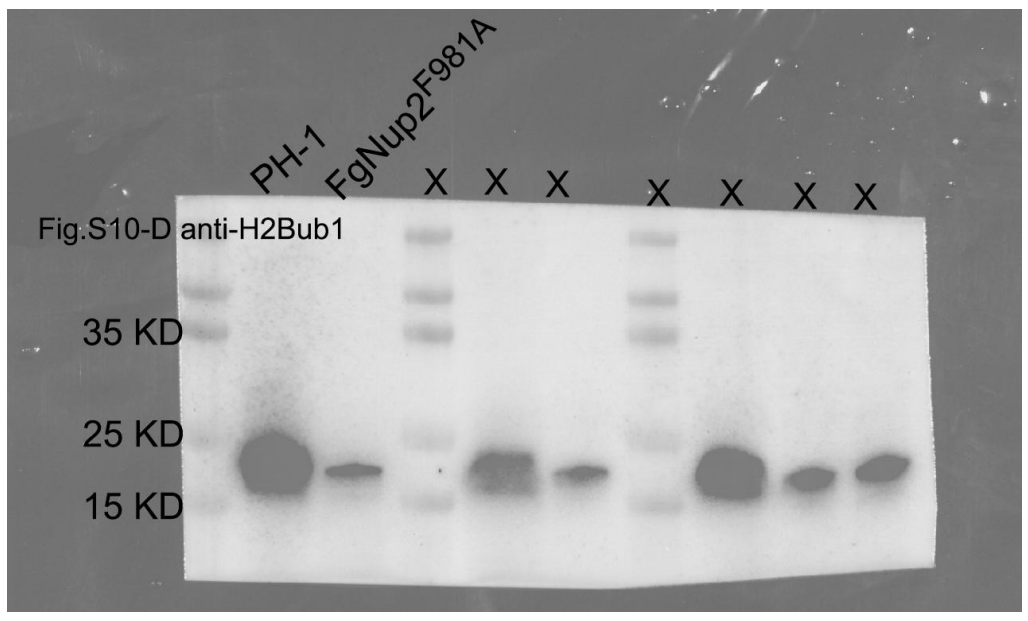
PH-1
FgNup2F981A
X X X X X X X
Fig.S10-D anti-H2Bub1
35 KD
25 KD
15 KD

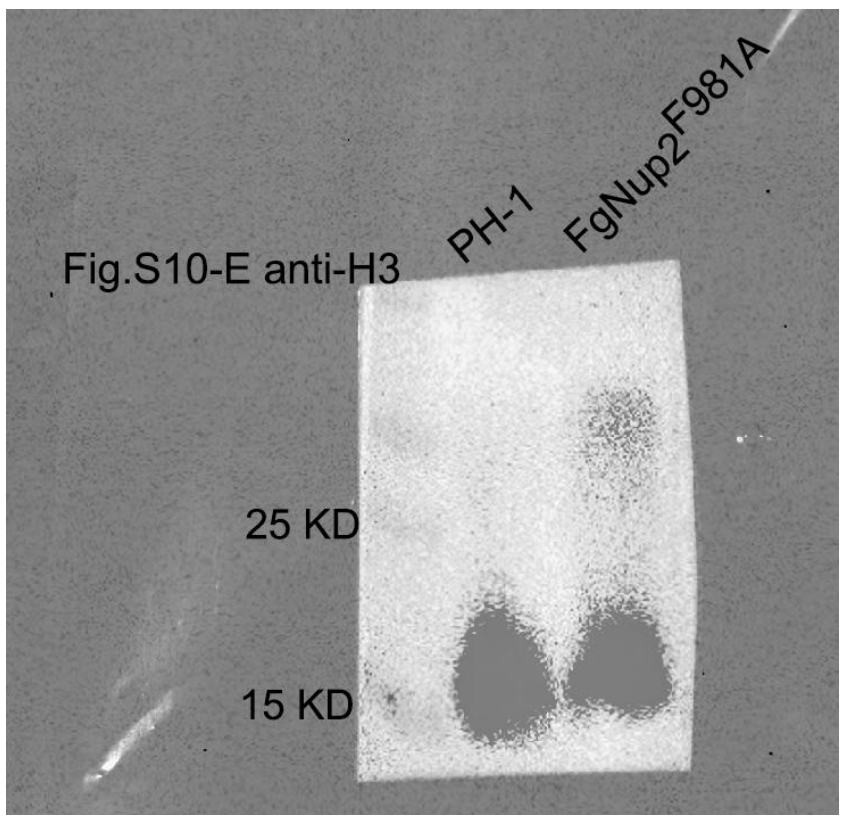
PH-1
FgNup2F981A
Fig.S10-E anti-H3
25 KD
15 KD

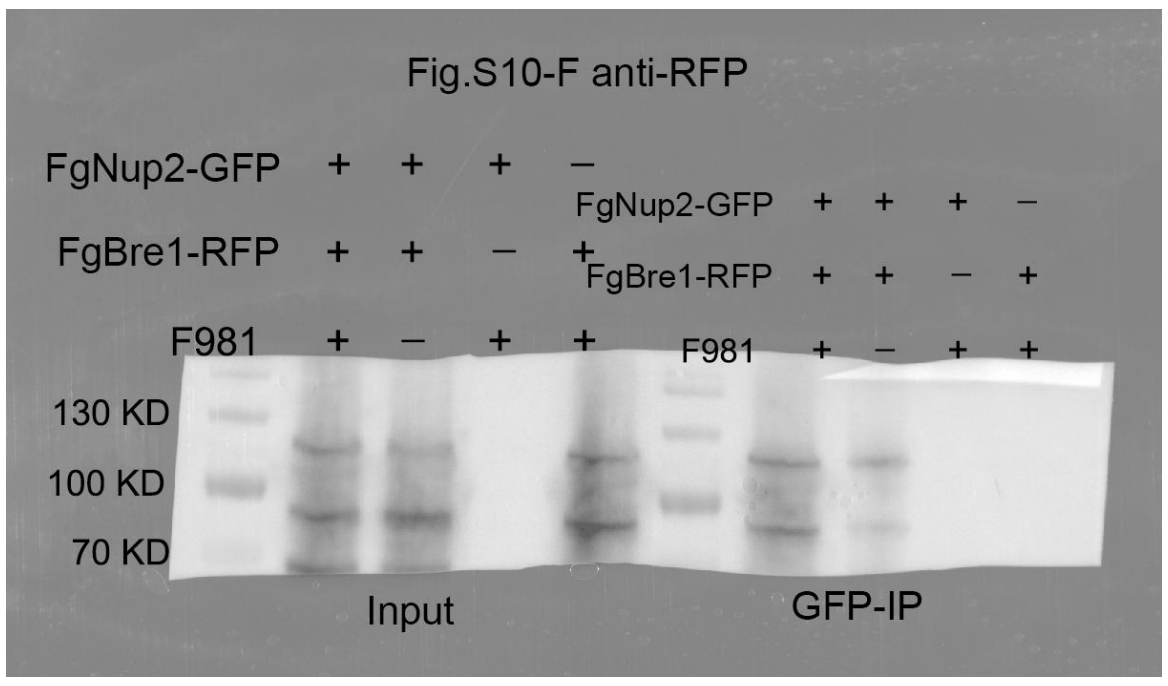
Fig.S10-F anti-RFP
FgNup2-GFP + + + –
FgBre1-RFP + + – +
F981 + – + +
FgNup2-GFP + + + –
FgBre1-RFP + + – +
F981 + – + +
130 KD
100 KD
70 KD
Input
GFP-IP

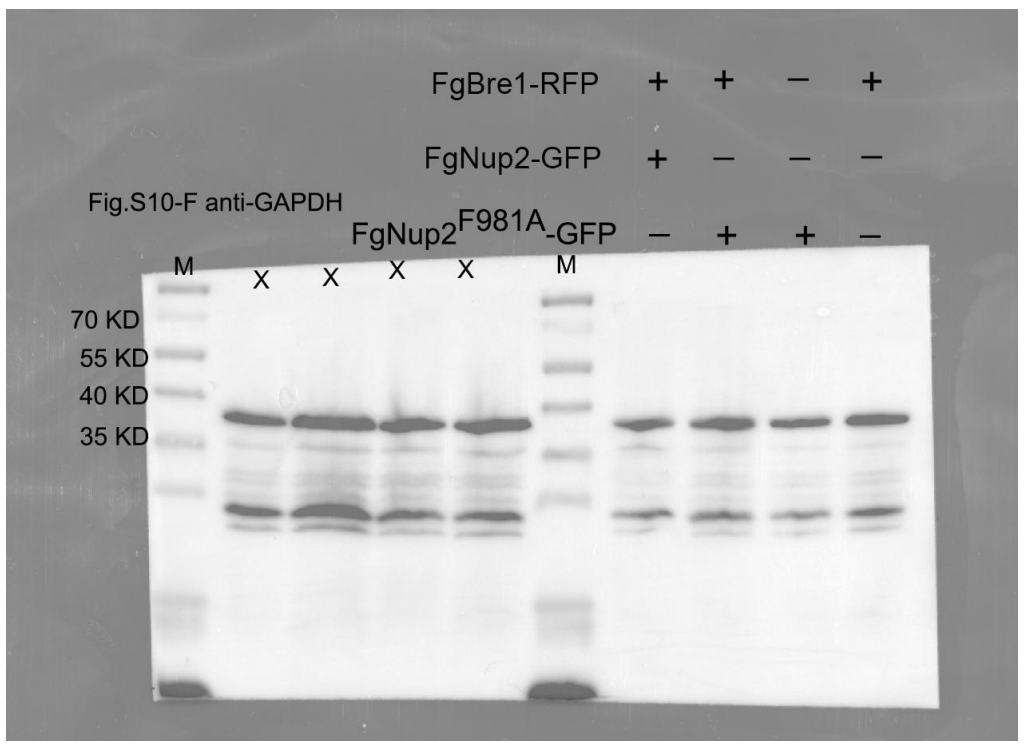
FgBre1-RFP + + – +
FgNup2-GFP + – – –
Fig.S10-F anti-GAPDH
FgNup2F981A-GFP – + + –
M X X X X M
70 KD
55 KD
40 KD
35 KD

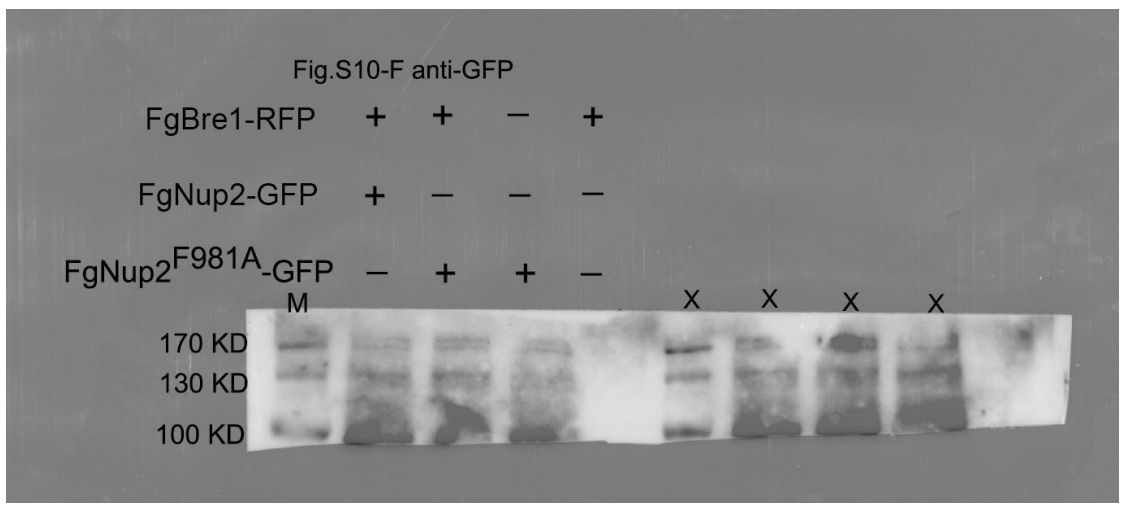
Fig.S10-F anti-GFP
FgBre1-RFP + + – +
FgNup2-GFP + – – –
FgNup2F981A-GFP – + + –
M
X X X X
170 KD
130 KD
100 KD
