# Supplementary material for: FgNup2 regulates nuclear import of the histone H2B monoubiquitination enzyme by stabilizing the FgImpα-FgBre1 complex to mediate pathogenicity in Fusarium graminearum
Source: PLoS Pathog. 2026 Sep 15;22(9):e1014577. doi: 10.1371/journal.ppat.1014577 (PMC13619055; doi:10.1371/journal.ppat.1014577)
Supplement: Supplementary file 2 [file ppat.1014577.s003.docx]

Tab.S1 Information on the Nups in *F. graminearum*

|  | Sub-NPC | Gene | Gene ID | | Location | | Amino acid (aa) | |
| --- | --- | --- | --- | --- | --- | --- | --- | --- |
|  | Central  Nups | Nup49 | FGSG_04165 | | 7151312-7153334 (+) | | 473 | |
|  |  | Nup57 | FGSG_09546 | | 6169803-6171385 (+) | | 327 | |
|  | Inner ring Nups | Nup170 | FGSG_05671 | | 3054116-3059023 (+) | | 1402 | |
|  |  | Nup188 | FGSG_05542 | | 2624356- 2630287 (+) | | 1818 | |
|  |  | Nup53 | FGSG_06402 | | 5272425-5276029 (+) | | 1257 | |
|  |  | Nup192 | FGSG_05518 | | 2532627-2538103 (−) | | 1664 | |
| Outer ring Nups | | Nup84 | | FGSG_01323 | | 4386752-4390179 (+) | 907 |  |
|  |  | Nup85 | | FGSG_10199 | | 10174999-10178746 (−) | 1081 |  |
|  |  | Nup160 | | FGSG_01156 | | 3777486-3781269 (−) | 1162 |  |
|  |  | Nup133 | | FGSG_09965 | | 9352521-9357598 (+) | 1381 |  |
|  |  | Seh1 | | FGSG_06185 | | 4596464-4598061 (−) | 443 |  |
|  |  | Sec13 | | FGSG_09271 | | 7104807- 7106441 (−) | 305 |  |
| Link Nups | | Nic96 | | FGSG_01429 | | 4733855-4737864 (−) | 1166 |  |
|  |  | Nup82 | | FGSG_05487 | | 2445630-2448875 (−) | 890 |  |
| Nuclear basket Nups | | Nup1 | | FGSG_06845 | | 1428042-1430579 (+) | 709 |  |
|  |  | Nup2 | | FGSG_09994 | | 9469650-9473996 (−) | 1208 |  |
|  |  | Mlp1 | | FGSG_05337 | | 1930271-1937051 (−) | 2066 |  |
|  |  | Mlp2 | | FGSG_16982 | | 2397525-2406203 (−) | 2398 |  |
| Transmembrane  Nups | | Pom34 | | FGSG_16813 | | 3118310-3119619 (+) | 357 |  |
|  |  | Pom152 | | FGSG_06361 | | 5137426-5141708 (−) | 1260 |  |
|  |  | Pom33 | | FGSG_04983 | | 802419 - 803784 (−) | 282 |  |
|  |  | Ndc1 | | FGSG_10865 | | 5916951-5919198 (+) | 620 |  |
| Cytoplas-mic Nups | | Nup42 | | FGSG_06067 | | 4197745-4200292 (−) | 643 |  |
|  |  | Nup159 | | FGSG_00366 | | 1183601-1188384 (−) | 1465 |  |

Tab.S2 Information on the Nups in *N.crassa*

| Nups name | *N.crassa* Gene ID | *N.crassa* Protein ID | Amino acid (aa) |
| --- | --- | --- | --- |
| Nup49 | NCU07655 | Q7SAX7 | 622 |
| Nup57 | NCU02473 | Q7SG91 | 253 |
| Nup170 | NCU04463 | Q7RYE4 | 1411 |
| Nup188 | NCU00199 | Q7RXM6 | 1858 |
| Nup192 | NCU00273 | Q7RZU0 | 1762 |
| Nup53 | NCU02025 | Q1K924 | 438 |
| Nup84 | NCU09814 | Q7SDI7 | 941 |
| Nup85 | NCU06649 | Q7S7I2 | 1010 |
| Nup160 | NCU02742 | Q7SGA8 | 1283 |
| Nup133 | NCU04007 | Q7RZJ5 | 1370 |
| Sec13 | NCU04063 | Q7RZF5 | 304 |
| Seh1 | NCU04376 | V5INM0 | 489 |
| Nic96 | NCU01702 | Q7RVQ2 | 1161 |
| Nup82 | NCU00209 | Q7RXL7 | 888 |
| Nsp1 | NCU02808 | Q7SEK6 | 682 |
| Nup2 | NCU06644 | Q1K6W4 | 1384 |
| Mlp1 | NCU04059 | Q7RZF8 | 2111 |
| Mlp2 | NCU00551 | Q7SF97 | 2418 |
| Ndc1 | NCU06776 | [Q1K8Z4](http://www.uniprot.org/uniprot/Q1K8Z4) | 632 |
| Pom33 | NCU06968 | Q7SAI1 | 286 |
| Pom34 | NCU06674 | Q1K6Y6 | 274 |
| Pom152 | NCU10747 | A7UWA2 | 1274 |
| Nup42 | NCU06894 | Q7S3F9 | 602 |
| Nup159 | NCU04047 | Q7RZG9 | 502 |

Tab.S3 Culture medium formula

| Medium | Detailed preparation protocol |
| --- | --- |
| Potato Dextrose Agar(**PDA)** | Wash and peel 200 g potatoes, cut into small pieces, Boil in 1 L distilled water for 30 min until soft, filter through cheesecloth, collect filtrate and adjust volume to 1 L, add 20 g glucose and 15–20 g agar, autoclave at 121°C for 20 min |
| Complete Medium(**CM)** | 10 g D-glucose, 1 g Casamino acid, 1 g Yeast extract, 2 g Peptone, 50 mL Nitrate salts, 1mL 1000×Trace elements, 1mL 1000×Vitamin solution, adjust volume to 1 L, adjust pH to 6.5, add agar if needed. Autoclave at 121°C for 20 min |
| Minimal Medium(**MM)** | 1 g KH₂PO₄, 0.5 g KCl, 2 g NaNO₃, 0.5 g MgSO₄·7H₂O, 0.01 g FeSO₄·7H₂O, 30 g Sucrose, 200 μL Trace elements(5 g ZnSO₄·7H₂O, 0.25 g CuSO₄·5H₂O, 1 g Fe(NH_4_)_2_(SO_4_)_2_·6H_2_O), adjust volume to 1 L, adjust pH to 6.5, add agar if needed, autoclave at 121°C for 20 min |
| Trichothecene Biosynthesis-inducing (**TBI)** | 1 g KH₂PO₄, 0.5 g KCl, 1.5 g Putrescine dihydrochloride, 0.5 g MgSO₄·7H₂O, 0.01 g FeSO₄·7H₂O, 30 g Sucrose, 200 μL Trace elements(5g Citric acid, 50 mg MnSO₄·H₂O, 0.25 g CuSO₄·5H₂O, 50 mg H_3_BO_3_, 50 mg Na_2_MoO_4_·6H_2_O), adjust volume to 1 L, adjust pH to 4.5, autoclave at 121°C for 20 min |
| Carboxymethylcellulose(**CMC)** | 15 g Carboxumethyl cellulose, 1 g NH_4_NO_3_,1g Yeast extract, 0.5 g MgSO₄·7H₂O, 1 g KH₂PO₄ adjust volume to 1 L, autoclave at 121°C for 20 min |

Tab.S4 Related primer sequences

| Primer name | Sequence (5’-3’) |
| --- | --- |
| FgNup2-up-F | CAGTATTCAGCAAGGAAGGA |
| FgNup2-up-R | CAAAATAGGCATTGATGTGTTGACCTCC  TGCGTGATGGTGGACGAAAG |
| FgNup2-down-F | CTCGTCCGAGGGCAAAGGAATAGAGTG  GCACACTATAACGTGCTTAC |
| FgNup2-down-R | ATCTATCAGGATAGCTCTGG |
| FgNup2-ID-F | CAGCTTCTCTAGCTGCATCT |
| FgNup2-ID-R | CTTGTAGTACCTAGGTAGTC |
| FgNup2-GFP-F | ACTCACTATAGGGCGAATTGGGTACTCAAATTGGTTCAGTATTCAGCAAGGAAGGA |
| FgNup2-GFP-R | CACCACCCCGGTGAACAGCTCCTCGCCCTTGCTCACCTTCTTATTATGAACCTTGT |
| FgNup2-GFP-ID-F | AGCAGGTCAACTTGGCCGAG |
| FgNup2-GFP-ID-R | GTCAGCTTGCCGTAGGTGGCA |
| HPH-F | GGAGGTCAACACATCAATGCCTATT |
| HPH-R | CTACTCTATTCCTTTGCCCT |
| FgBre1-up-F | CAAAATAGGCATTGATGTGTTGACCTCCCTTGGCGAGTGTA |
| FgBre1-up-R | CTATAGGGCGAATTGGGTACTCAAATTGGTTGTGAGTAGGCCTCATAGCGAC |
| FgBre1-down-F | CTCGTCCGAGGGCAAAGGAATAGAGTAGTCCGTCAAGCATTGCTCATC |
| FgBre1-down-R | CCAATGGAGCTGCACTGCGT |
| FgBre1-ID-F | TGAGGCTAGACCGACACT |
| FgNic96-up-F | GCATATCTATGACTTCCTCA |
| FgNic96-up-R | CAAAATAGGCATTGATGTGTTGACCTCCGGATGCTTGGAGGTGAAGCT |
| FgNic96-down-F | CTCGTCCGAGGGCAAAGGAATAGAGTAGCTCCTGGTGTTGGTTGTAGT |
| FgNic96-down -R | GAGTTACTATGGGTATCCAC |
| FgNic96-ID-F | GGCCGATACGAAGTAACTAC |
| FgNic96-ID-R | GCTCTTGATCATGTGATATC |

ContinueTab.S4 Related primer sequences

| Primer name | Sequence (5’-3’) |
| --- | --- |
| FgNup82-up-F | CACCGTCTCGTCTTACCACT |
| FgNup82-up-R | CAAAATAGGCATTGATGTGTTGACCTCCTGGCGAAGAATAGAGGTGTC |
| FgNup82-down-F | CTCGTCCGAGGGCAAAGGAATAGAGTAGCGATGTCATGTAACGACCAT |
| FgNup82-down-R | CAGAGATCGAGAAGCTCATG |
| FgNup82-ID-F | GACACCTCTATTCTTCGCCA |
| FgNup82-ID-R | TGTATGGTCGCACCGAGCAC |
| FgNup53-up-F | CATCCAATGCGCCGAGAAGA |
| FgNup53-up-R | CAAAATAGGCATTGATGTGTTGACCTCCCCGGTTCCGTTGGAGCTTA |
| FgNup53-down-F | CTCGTCCGAGGGCAAAGGAATAGAGTAGAGGATTCAGAAATCCGGCCA |
| FgNup53-down-R | AAGCCCAGAGCAGAGAGATA |
| FgNup53-ID-F | GTTGAGGCTCTCTCTATAAG |
| FgNup53-ID-R | GTGGCGGAATCAGATATTGA |
| FgNup133-up-F | GATATATCTTGTGTAAGTGC |
| FgNup133-up-R | CAAAATAGGCATTGATGTGTTGACCTCCGCGTGCATCGGTAGCCAATC |
| FgNup133-down-F | CTCGTCCGAGGGCAAAGGAATAGAGTAGATGTTTATAAATGTCTTGCC |
| FgNup133-down-R | TCGAGAAGCTTGAGCTGCAG |
| FgNup133-ID-F | GCAATGCCAGCTCAGCACAG |
| FgNup133-ID-R | AATGAAACAAGACTGCAGCC |
| FgNup192-up-F | CGCTCTCAGAGAAGAGCAGT |
| FgNup192-up-R | CAAAATAGGCATTGATGTGTTGACCTCCCGGTTCAGGACTGTCGCATG |
| FgNup82-down-F | CTCGTCCGAGGGCAAAGGAATAGAGTAGGACACCTACGATAAGAAGAG |
| FgNup192-down-R | AGTTGAAGCTTATGACTGCC |
| FgNup192-ID-F | AGTGGCAGAAAGCCACTCAC |
| FgNup192-ID-R | AGTTGAAGCTTATGACTGCC |

Continue Tab.S4 Related primer sequences

| Primer name | Sequence (5’-3’) |
| --- | --- |
| FgSeh1-up-F | GTGTGAGGCATTGTGAAGTG |
| FgSeh1-up-R | CAAAATAGGCATTGATGTGTTGACCTCCGTTGTTGACATAGCTCCACA |
| FgSeh1-down-F | CTCGTCCGAGGGCAAAGGAATAGAGTAGGATACAGTCTCAACTTCTTG |
| FgSeh1-down-R | ATGAATTGTGCATGACTTCC |
| FgSeh1-ID-F | GAGGTCATCGAAATCGTGGT |
| FgSeh1-ID-R | AACTGCCGGTCACAACTCGG |
| FgSec13-up-F | AGGTGAGAAAGCGGTGTTGG |
| FgSec13-up-R | CAAAATAGGCATTGATGTGTTGACCTCCTGGTGACAGTTGGCCAAGCT |
| FgSec13-down-F | CTCGTCCGAGGGCAAAGGAATAGAGTAGTAAGCAATGATGAACTAGGT |
| FgSec13-down-R | GAACTCGGCTGATGAACTGC |
| FgSec13-ID-F | AGCGGTAAGTGGGGGTTGAC |
| FgSec13-ID-R | GCAGGCCTGCTACATTCAGG |
| FgPom33-up-F | TCATCAGTAATGACACTGG |
| FgPom33-up-R | CAAAATAGGCATTGATGTGTTGACCTCCAGCGTAGGTTGGTTGAAGA |
| FgPom33-down-F | CTCGTCCGAGGGCAAAGGAATAGAGTAGGATTGGTCATGGAATTGGTA |
| FgPom33-down-R | TGTTCTTGAGTGAGAGTACC |
| FgPom33-ID-F | GCCTATCCTCTCTAAAGTGC |
| FgPom33-ID-R | ATCGCATTGCCTCAGTCTG |
| FgPom152-up-F | CTGCTGATCCGAGCCTGATT |
| FgPom152-up-R | CAAAATAGGCATTGATGTGTTGACCTCCCCAAGTCGCAGCCGATGAAT |
| FgPom152-down-F | CTCGTCCGAGGGCAAAGGAATAGAGTAGGATAGGTAATGAATGGATGGA |
| FgPom152-down-R | GAATAGTTGCCAATCACTTC |
| FgPom152-ID-F | GTATCCACTGTAGCTCCAG |
| FgPom152-ID-R | ACATAAGTATTCAACCTTAG |

Continue Tab.S4 Related primer sequences

| Primer name | Sequence (5’-3’) |
| --- | --- |
| FgBre1-ID-F | GATATCTCGCTGCGCAAGT |
| RT- Tri1-F | GATGTTCCTTCTCGACAGCGT |
| RT- Tri1-R | CACTGGTCGAAGATAGCTGG |
| RT- Tri3-F | TGTTACGATCAATGGCTTGG |
| RT- Tri3-R | TCCTCGTTGTAGTTTGCATCA |
| RT- Tri4-F | ACGTGTGGCTACTCAGGAGA |
| RT- Tri4-R | TGGAATTGCCTTGGGGTA |
| RT- Tri5-F | ATGGCGGATCTATCTATTCAC |
| RT- Tri5-R | CCATTCATACGACGAAGGAAT |
| RT- Tri6-F | AAATGCCCATTCCCTAGTTG |
| RT- Tri6-R | ATCTCGCATGTTATCCACCCT |
| RT- Tri7-F | TACCGTCGTCTTCAAAACCA |
| RT- Tri7-R | ACGCCAATGGTGTTCACAAA |
| RT- Tri8-F | ATATAACGGTACCCCCAGATG |
| RT- Tri8-R | TGTTTGTAGGACACTTCCGGT |
| RT- Tri9-F | CCGCTAAACTGATCGACTCAT |
| RT- Tri9-R | CCCATATGGTAGCGCATAAA |
| RT- Tri10-F | TCCCAACCTTTCAGAGGTTCA |
| RT- Tri10-R | TGATCCGTCAAGTCTTCCCAT |
| RT- Tri11-F | TGAGAACGACATGTGGGCAAT |
| RT- Tri11-R | AGGCTTGTTCCATGCAAGAT |
| RT- Tri12-F | ACGAACAGCACTGCTACGGT |
| RT- Tri12-R | TTCCTGCTTGTGACTCCAAT |
| RT- Tri13-F | ACGCAGATCCTGGGATATCA |
| RT- Tri13-R | CAGCCCAGTATTTTGCCAAA |
| RT- Tri14-F | AACTCCCGTTGTGATCAAGCA |
| RT- Tri14-R | AACAGTAATGTTGGCACCGT |
| RT- Tri101-F | CATCACGCCTTCGTTCAGAAC |
| RT- Tri101-R | CATCAAGCTCTCAACAGGCT |
| RT- Actin-F | ATCCACGTCACCACTTTCAA |
| RT- Actin -R | TGCCTTGAGATCCACATTTG |
| 68-ID-R | CGTCCATGCCGAGAGTGATC |

Continue Tab.S4 Related primer sequences

| Primer name | Sequence (5’-3’) |
| --- | --- |
| FgBre1-AD-R | TATCGATGCCCACCCGGGTGGAATTCTCAATGATGAACGGGCATAACGT |
| FgBre1-68-F | TTTCGTAGGAACCCAATCTTCAAAATGGAGGATAGAAAGCGGCC |
| FgBre1-68-R | GTTCGGGATCTTGCAGGCCGGGCGATGATGAACGGGCATAACGT |
| FgBre1-RP27-GFP-F | TTTCGTAGGAACCCAATCTTCAAAATGGAGGATAGAAAGCGGCC |
| FgBre1-RP27-GFP-F | CACCACCCCGGTGAACAGCTCCTCGCCCTTGCTCACATGATGAACGGGCATAACGT |
| FgBre1-GST-F | CCAAAATCGGATCTGGTTCCGCGTGGGATCCATGGAGGATAGAAAGCGGCC |
| FgBre1-GST-R | CTCGAGTCGACCCGGGAATTCTCAATGATGAACGGGCATAACGT |
| ID-GST-R | CCTGACGGGCTTGTCTGCTC |
| FgNup2-65-F | TTTCGTAGGAACCCAATCTTCAAAATGGTTACCTTCTCTATCCC |
| FgNup2-65-R | GCTCACCATCGTGGCGATGGAGCGCTTCTTATTATGAACCTTGT |
| FgNup2-His-F | TGGACAGCAAATGGGTCGCGGATCCATGGTTACCTTCTCTATCC |
| FgNup2-His-R | GTGGTGCTCGAGTGCGGCCGCAAGCTTTTACTTCTTATTATGAACCT |
| FgImp-α-GFP-F | ACTCACTATAGGGCGAATTGGGTACTCAAATTGGTTGAAGTGAATTAACGCGACC |
| FgImp-α-GFP-R | CACCACCCCGGTGAACAGCTCCTCGCCCTTGCTCACCATGTCCATGGCGTCGTTAC |
| ID-Imp-α-GFP-F | ACCTGGTTGCTCAAGGCTGC |
| FgNup2-BD-F | TGATCTCAGAGGAGGACCTGCATATGATGGTTACCTTCTCTATCC |

Continue Tab.S4 Related primer sequences

| Primer name | Sequence (5’-3’) |
| --- | --- |
| FgNup2-BD-R | GCAGGTCGACGGATCCCCGGGAATTCTTACTTCTTATTATGAACCTTGT |
| FgNup2-BD-1-F | TGATCTCAGAGGAGGACCTGCATATGATGGTTACCTTCTCTATCC |
| FgNup2-BD-1-R | GCAGGTCGACGGATCCCCGGGAATTCTTAGGCAGCAGCATTGTCC |
| FgNup2-BD-2-F | GATCTCAGAGGAGGACCTGCATATGATGGCACCCGGTAACAATG |
| FgNup2-BD-2-R | GCAGGTCGACGGATCCCCGGGAATTCTTAGCCTCCGGCGGTTCC |
| FgNup2-BD-3-F | TGATCTCAGAGGAGGACCTGCATATGATGGAGCAGGACGAAGATG |
| FgNup2-BD-3-R | GCAGGTCGACGGATCCCCGGGAATTCTTACTTCTTATTATGAACCTTG |
| NLS-68-F | TTTCGTAGGAACCCAATCTTCAAAATGGACTACAAAGACCATG |
| NLS-68-R | GTTCGGGATCTTGCAGGCCGGGCGTAAGGTAAGTCTTTCTAG |

Fig. S1 Schematic representation of the predicted domain features of *F. graminearum* Nups. Different coloured blocks represent different conserved protein domains, and the black line represents the number of amino acids.

Fig. S2 Analysis of *FgNups* expression levels during wheat infection from 0-7 d

Fig. S3 Specific upregulation and knockout of FgNups genes in *F. graminearum*. (A) PCR identification of gene knockouts *ΔFgNup2*, *ΔFgNup53*, *ΔFgNup133*, *ΔFgSeh1*, *ΔFgSec13*, *ΔFgNup192*, *ΔFgNup82*, *ΔFgPom34*, *ΔFgPom152* and *ΔFgNic96*. (B) Southern blot verification of the above mutants.

Fig S4 Heatmap of RPKM-normalised transcripts, depicting 3807 differentially expressed genes from RNA-seq

Fig. S5 In *ΔFgNup2* and PH-1, the transcriptional expression level of the *FgBre1* gene was detected by RT-qPCR

Fig. S6 Subcellular localization of FgBre1 under infection conditions. (A) Schematic representation of the predicted domain features of FgBre1. Different coloured blocks represent different conserved protein domains, and the black line represents the number of amino acids. (B) Fluorescence stereomicroscopy showing the localization of Bre1-GFP in PH-1 and *ΔFgNup2* during infection. (C) Confocal microscopy images depicting the subcellular distribution of Bre1-GFP in hyphae of PH-1 and *ΔFgNup2* at the infection stage.

Fig. S7 Quality control and peak distribution of ChIP-seq data. (A) Principal component analysis of all ChIP-seq and Input samples. (B) Cumulative coverage percentage distribution chart of reads. (C) Viewing the distribution map of the reads gene region, the horizontal axis represents each region of the gene, and the vertical axis represents the enrichment intensity of the reads. ChIP data usually show binding peaks near the TSS (±2 kb of TSS) for all samples. (D) Aggregate peak density profiles across normalized gene regions for PH-1 and *ΔFgNup2*. (E) Peak length distribution density curves for PH-1 and *ΔFgNup2*.

Fig S8 RT-qPCR detection of transcriptional expression levels of the genes shown in panel A in *ΔFgNup2* and PH-1

Fig. S9 Identification of key functional regions within the FG repeat sequence of FgNup2 protein. Schematic diagram of the systematic deletion mutation strategy of FgNup2-2 domain. 143-amino acid region was deleted in units of 8 consecutive amino acids, constructing a total of 18 deletion fragments. Each deletion mutant vector was transformed into the *ΔFgNup2* mutant to obtain complementary strains *Δ1–Δ18*.

Fig. S10 Identification of key single amino acid sites in the FG repeat sequence of the FgNup2 protein. Schematic diagram of the strategy for alanine single-point mutation at amino acids 980-987 of FgNup2. Colony morphology of point mutation complementation strains on PDA plates after 72 h of growth.

Fig S11 (A) Colony morphology of wild-type PH-1 and FgNup2^F981A^ point mutant strains after 72 h of cultivation on PDA, CM, and MM media. (B) Statistical analysis of lesion lengths of PH-1 and FgNup2^F981A^ in the wheat coleoptile inoculation test. (C) Measurement of DON toxin production by the two strains under toxin-inducing conditions. The data come from three independent repeated experiments, and significant differences are indicated by * (****P≤0.0001). (D) 6 h after induction, the protein distribution of FgBre1 in the nuclear and cytoplasmic fractions of the FgNup2^F981A^ strain was detected by Western Blot. (E) The overall level of H2Bub1 modification in the FgNup2^F981A^ strain was detected by Western Blot. (F) Co-IP experiments were used to detect the interaction between FgBre1 and FgNup2 in PH-1 and *ΔFgNup2* strains.
